# Genome-guided discovery of tropansamycins: antimicrobial pentaketide ansamycins

**DOI:** 10.1016/j.engmic.2026.100263

**Published:** 2026-02-12

**Authors:** Haotian Wang, Run Jiao, Liran Ma, Yaoyao Li, Yuemao Shen, Haoxin Wang

**Affiliations:** a State Key Laboratory of Microbial Technology, Shandong University, Qingdao 266237, China; b Key Laboratory of Chemical Biology of Ministry of Education, School of Pharmaceutical Sciences, Cheeloo College of Medicine, Shandong University, Jinan 250012, China

**Keywords:** Ansamycin, *Streptomyces*, Genome mining, Antibacterial activity

## Abstract

Although ansamycins are clinically important macrolactams, their pentaketide subset exhibits limited biological activities. Genome mining of rhizosphere-derived *Streptomyces* sp. LR53 uncovered a cryptic pentaketide ansamycin gene cluster (*tpm*). Activation via 3-amino-5-hydroxybenzoic acid (AHBA) feeding, promoter replacement, and deletion of the cytochrome P450 (CYP450) gene *tpm16* enabled the discovery of seven novel metabolites, tropansamycins A–G (**1**–**7**), establishing the seventh distinct pentaketide ansamycin scaffold. Notably, congeners **5** and **6**, predominantly accumulated in the Δtpm16 mutant, exhibited potent activity against gram-negative pathogenic bacterium *Xanthomonas oryzae* and gram-positive bacterium *Staphylococcus aureus*, with MICs ranging from 2 to 8 µg/mL and were toxic to the producer strain cultivated on LB agar medium. This reveals that the CYP450 enzyme Tpm16 functions as a modification enzyme modulating the bioactivity profile of pathway intermediates. These findings not only expand the structural diversity of ansamycins but also highlight the potential of these pathogen-active ansamycins as promising leads.

## Highlights

• A “cryptic” pentaketide ansamycin gene cluster was activated.
• Seven novel pentaketide ansamycins were isolated from *Streptomyces*.
• Compounds **5** and **6** were active against *Xanthomonas oryzae* and *Staphylococcus aureus*.
• CYP450 enzyme Tpm16 modulates the bioactivity of pathway intermediates.

## Graphical abstract

**Figure:**
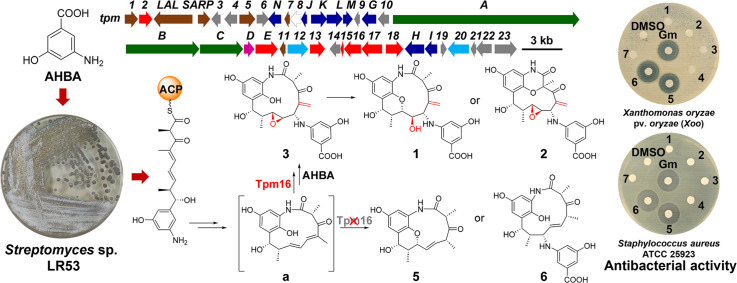

## 1 Introduction

Ansamycins are a prominent family of macrolactam natural products from actinomycetes and have yielded several clinically important drugs [[1], [2], [3]]. Representative members include rifamycins, first-line anti-tuberculosis agents [3], maytansinoids, potent anti-tumor agents [4], and geldanamycin, a prototypical Hsp90 inhibitor [5,6]. All ansamycins are assembled by type I polyketide synthases (PKSs) that employ the rare starter unit 3-amino-5-hydroxybenzoic acid (AHBA) and are released by a dedicated amide synthase. Genome-guided mining has greatly expanded the structural repertoire of the family over the past decade [[7], [8], [9], [10], [11], [12], [13], [14]], revealing an emerging subset of pentaketide ansamycins. The reported pentaketide ansamycins can be categorized into the six scaffolds based on the differences in extender units: Q1047s/cebulactams/goondansamycins/shengliangmycins [7,[14], [15], [16]], catellatolactams [8], aminoansamycins [9], microansamycins [10], juanlimycins [12], and tetrapetalones [17] (Fig. S1). Despite their structural novelty, these compounds have shown only moderate antioxidant activity and lack antimicrobial properties [7,17].

**Fig. 1:**
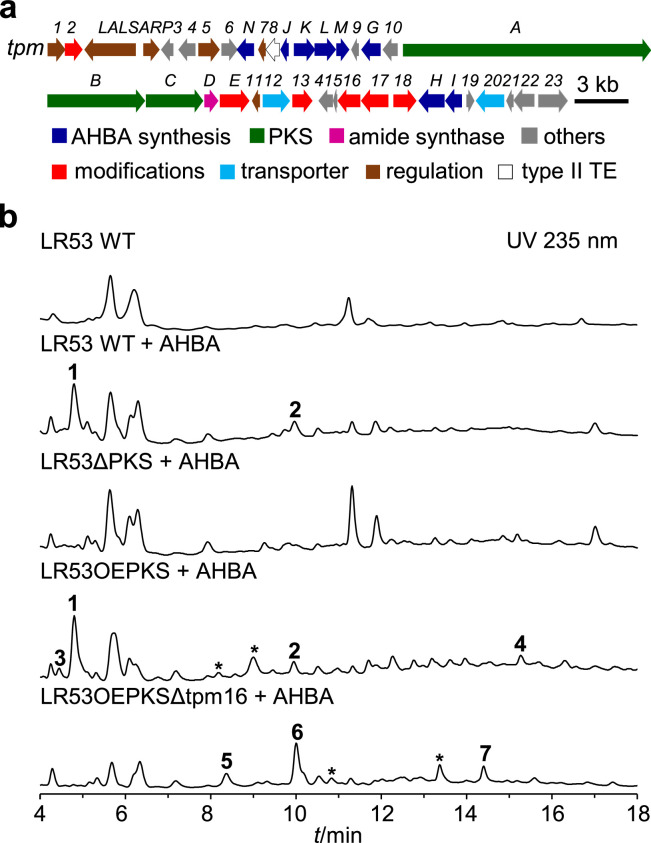
(A) Gene organization of the *tpm* cluster in *Streptomyces* sp. LR53. (B) HPLC profiles of the metabolites of LR53 WT, LR53ΔPKS, LR53OEPKS, and LR53OEPKSΔtpm16 supplemented with AHBA (200 mg/L). Peaks corresponding to uncharacterized tropansamycin analogues are marked with asterisks.

During a genome survey of rhizosphere-derived actinomycetes, we detected a “cryptic” ansamycin gene cluster *tpm* in *Streptomyces* sp. LR53 (Fig. 1a, Table S1), an isolate from the soil collected from Xishuangbanna Tropical Botanical Garden, Yunnan, China. Here we report activation of the *tpm* pathway via precursor (AHBA) directed feeding, promoter engineering, and subsequent gene-deletion experiments. This led to the discovery of seven new pentaketide ansamycins, tropansamycins A–G (**1**–**7**), representing the seventh distinct pentaketide ansamycin scaffold. Notably, deletion of the CYP450 gene *tpm16* led to the accumulation of two congeners (**5** and **6**), which displayed potent activity against plant pathogens and the producer strain itself. This finding suggests that Tpm16 acts as a modification enzyme modulating the bioactivity profile of pathway intermediates. Based on these results, we also proposed a plausible biosynthetic pathway for tropansamycins.

## 2 Materials and Methods

### 2.1 General

Culture conditions and instruments are detailed in the Supplementary Materials. Strain and plasmid details are provided in Table S2, while primers are listed in Table S3.

### 2.2 Generation of the PKS gene tpmA disrupted and overexpressed mutants

To generate the *tpmA* disruption mutant, homologous arms were amplified from *Streptomyces* sp. LR53 genomic DNA using the ΔPKS-F/R primer pairs (Table S3), then digested with *Bgl*II/*Eco*RI and ligated into pOJ260, creating pOJ260-ΔPKS. This construct was transformed into *E. coli* ET12567/pUZ8002 and conjugated with strain LR53. Single-crossover exconjugants were selected on apramycin-containing SFM agar plates and verified using PCR with verΔPKS-F/R primers (Table S3, Fig. S2a). A suicide plasmid with a melanin reporter (for black pigment) was constructed to facilitate double-crossover screening. Specifically, the melanin reporter cassette *ermE*p*-*melC1C2* was amplified from pMEL [18] using PCR and the EmelC-F/R primer pair, and then seamlessly assembled via Gibson assembly with a PCR product amplified from pOJ260 using primers POJ260m-F/R, resulting in pSPRm. For the *tpmA* overexpression mutant, homologous arms were amplified from strain LR53 genomic DNA using OEPKSup-F/R and OEPKSdn-F/R primer pairs (Table S3), and the *kasO*p* fragment was amplified from pUC-*kasO*p*-T [19] using PCR with kas-F/R primers and digested with *Mfe*I/*Nde*I. These three fragments were assembled into the *Hin*dIII/*Nco*I site of the pSPRm vector using Gibson assembly, generating the pSPRm-OEPKS construct. Following transformation into *E. coli* ET12567/pUZ8002 and conjugation with strain LR53, single-crossover exconjugants were selected on apramycin-containing SFM agar plates, with double-crossover mutants identified by screening for melanin-deficient colonies. The mutant strains were ultimately confirmed using PCR with verOEPKS-F/R primers (Table S3, Fig. S3).

### 2.3 Generation of recombinants with overexpressed regulatory genes

The *kasO*p* fragment was amplified from pUC-*kasO*p*-T [19] using PCR with kas-F/R primers, and the *ermE*p* fragment was amplified from pJTU824 [20] using PCR with erm-F/R primers (Table S3). The above fragments were co-ligated into the *Eco*RI/*Pst*I site of pSET5035 using Gibson assembly, yielding pSET5035-KE. The *tpmLAL* and *tpmSARP* genes were amplified from the genomic DNA of strain LR53 using PCR with primers of LAL-F/R and SARP-F/R (Table S3), respectively. The resulting fragment was individually cloned into the *Nde*I/*Spe*I site of pSET5035-KE under the control of the kasO* promoter, yielding pSET5035-LAL and pSET5035-SARP. The *Eco*RI/*Spe*I-digested *kasO*p*-LAL fragment from pSET5035-LAL and the *Xba*I/*Nsi*I-digested *kasO*p*-SARP fragment from pSET5035-SARP were co-inserted into the *Eco*RI/*Nsi*I site of pSET5035-KE, yielding pSET5035-LS. These plasmids were individually introduced into *E. coli* ET12567/pUZ8002 and subsequently conjugated with strain LR53, yielding three recombinant strains, LR53LAL, LR53SARP, and LR53LS, respectively. The recombinant strains were confirmed using PCR with primers of ver-F/verLAL-R, ver-F/verSARP-R, and ver-F/verLS-R, respectively (Table S3, Fig. S4a).

### 2.4 Generation of gene-deleted mutants

Homologous arms were amplified from the genomic DNA of *Streptomyces* sp. LR53 using PCR with ΔLALup-F/R and ΔLALdn-F/R primers (Table S3) to generate the regulatory gene *tpmLAL* deletion mutant. The resulting fragments were individually digested with *Hin*dIII/*Mfe*I and *Mfe*I/*Nco*I and ligated into the *Hin*dIII/*Nco*I site of the pSPRm vector using T4 DNA ligase, creating the pSPRm-ΔLAL construct. This construct was transformed into *E. coli* ET12567/pUZ8002 and conjugated with strain LR53. Similarly, homologous arms were amplified from the genomic DNA of strain LR53OEPKS with Δtpm16/17up-F/R and Δtpm16/17dn-F/R primers (Table S3) to delete the oxidoreductase genes *tpm16* and *tpm17*. These fragments were assembled into the *Hin*dIII/*Nco*I site of the pSPRm vector using Gibson assembly, generating the pSPRm-Δtpm16/17 construct. Both constructs were independently transformed into *E. coli* ET12567/pUZ8002 and conjugated with strain LR53OEPKS. Exconjugants were identified on apramycin-containing SFM agar plates, while double-crossover mutants were obtained by screening for melanin-deficient colonies. The mutants were verified using PCR with primers targeting the regions flanking and within the knockout genes: verLALout-F/R and verLALin-F/R for *tpmLAL* (Table S3, Fig. S5a), and verΔtpm16-F/R and verΔtpm17-F/R for *tpm16/17* (Table S3, Fig. S6a).

### 2.5 Fermentation and metabolite analysis of strains

Strain LR53 and the mutants were cultivated on ISP3 agar medium at 30 °C for 9 d. The cultured mycelia were subsequently cut into minute pieces and extracted overnight at ambient temperature using a solvent mixture of EtOAc/MeOH/AcOH (80/15/5, v/v/v). Pooled extracts were dried and dissolved in MeOH, with 20 µL aliquots analyzed using HPLC. Mobile phases were: A, water with 0.1% formic acid (FA); B, CH_3_CN with 0.1% FA. The gradient program was: 20% B for the first 3 min, increased to 35% B over the next 12 min, held at 35% B for 2 min, increased to 55% B over the next 10 min, then to 100% B at 26 min, held at 100% B for 2 min, decreased to 20% B at 30 min, and held at 20% B for 2 min.

### 2.6 Isolation of compounds **1**−**7**

Strain LR53 was cultured on ISP3 agar medium with 200 mg/L AHBA at 30 °C for 9 d. The culture was extracted three times with EtOAc/MeOH/AcOH (80/15/5, v/v/v). The pooled extracts were concentrated and subsequently subjected to four sequential partitions using EtOAc and H_2_O (1:1, v/v). The EtOAc fraction was then further partitioned between 95% MeOH and petroleum ether (1:1, v/v) to yield the MeOH extract. The extract (3.63 g) was chromatographed on Sephadex LH-20 (120 g) using MeOH as the mobile phase, yielding two fractions. Fr.1 (155.2 mg) was initially separated via medium-pressure liquid chromatography (MPLC) on a RP-18 column (20 g) using 15% CH_3_CN, producing subfraction Fr.1a. This subfraction (5.6 mg) was further refined through semipreparative HPLC with a 15% CH_3_CN eluent, ultimately yielding **1** (*t_R_* = 6.2 min, 2.3 mg). Fr.2 (90.1 mg) was processed using MPLC on a RP-18 column (20 g) with 20% CH_3_CN, generating subfraction Fr.2a. Subsequent semipreparative HPLC using 21% CH_3_CN as the mobile phase enabled the isolation of **2** (*t_R_* = 6.3 min, 1.2 mg)

The crude extract from the 10 L fermentation of LR53OEPKS was obtained using the same method as described for LR53 WT. The MeOH extract (4.8 g) was chromatographed on Sephadex LH-20 (120 g) using MeOH as the eluent, yielding four fractions (Fr.1–4). These fractions were individually chromatographed using MPLC (RP-18, 20 g) with varying concentrations of CH_3_CN: Fr.1 (114.0 mg) at 26%, Fr.2 (155.2 mg) at 13%, Fr.3 (180.2 mg) at 20%, and Fr.4 (71.7 mg) at 13%. This process yielded the subfractions Fr.1a–4a, respectively. Semipreparative HPLC was then performed using CH_3_CN as the eluent, with the following concentrations: Fr.1a (5.6 mg) at 30%, Fr.2a (32.6 mg) at 15%, Fr.3a (6.2 mg) at 21%, and Fr.4a (6.0 mg) at 15% to obtain **4** (*t_R_* = 6.3 min, 1.5 mg), **1** (*t_R_* = 6.4 min, 5.6 mg), **2** (*t_R_* = 6.7 min, 3.4 mg), and **3** (*t_R_* = 5.1 min, 3.1 mg), respectively.

The crude extract from the 10 L fermentation of LR53OEPKSΔtpm16 was obtained using the same method as described for LR53 WT. The MeOH extract (4.2 g) was chromatographed on Sephadex LH-20 (120 g) using MeOH as the mobile phase, yielding three fractions (Fr.1–3). Fr.1 (137.8 mg) was subjected to MPLC on a RP-18 column (20 g) with 12% CH_3_CN, producing subfraction Fr.1a. This subfraction (12.6 mg) underwent further purification via semipreparative HPLC using 20% CH_3_CN, yielding **5** (*t_R_* = 8.4 min, 3.1 mg). Fr.2 (153.0 mg) was processed by MPLC on a RP-18 column (20 g) using a step gradient of CH_3_CN (20%, 22%, 24%, 200 mL each), resulting in subfraction Fr.2a. Subsequent semipreparative HPLC with 28% CH_3_CN enabled the isolation of **7** (*t_R_* = 8.7 min, 7.2 mg). Fr.3 (91.4 mg) underwent MPLC on a RP-18 column (20 g) with a CH_3_CN step gradient (14%, 16%, 18%, 200 mL each), generating subfraction Fr.3a. Final purification by semipreparative HPLC using 21% CH_3_CN obtained **6** (*t_R_* = 6.7 min, 7.1 mg).

### 2.7 Biological activity assay

The antimicrobial activities of **1**–**7** were evaluated using disk diffusion and broth dilution methods against a diverse array of microorganisms, encompassing gram-negative bacteria (*Xanthomonas oryzae* pv. *oryzae, Xanthomonas oryzae* pv. *oryzicola*, and *Pseudomonas aeruginosa* PAO1), gram-positive bacteria (*Bacillus subtilis, Staphylococcus aureus* ATCC 25923, *Mycobacterium smegmatis* MC^2^ 155, and *Streptomyces* sp. LR53OEPKS), and fungi (*Candida albicans* 5314, *Colletotrichum gloeosporioides* lq27, *Aspergillus niger*, and *Aspergillus flavus* ACCC 32636). Bacterial and yeast strains were cultivated on Luria–Bertani (LB) agar medium, the *Streptomyces* strain was cultivated on both LB and ISP3 agar media, and filamentous fungal strains were grown on potato dextrose agar (PDA) medium. Sterile filter paper disks (5 mm) were loaded with 20 µg of each compound, with gentamicin (20 µg), apramycin (5 µg), nystatin (15 µg), and caspofungin (10 µg) as positive controls and DMSO as a blank control. A broth dilution method using 96-well plates and potato dextrose broth (PDB) for filamentous fungi and LB broth for other strains was used for minimum inhibitory concentration (MIC) determination. Each well contained 100 µL of bacterial or spore suspension and 100 µL of antimicrobial solution, creating concentration gradients from 0 to 64 µg/mL. MIC was determined by the absence of visible microbial growth. Concurrently, the Cell Counting Kit-8 (CCK-8) assay [21] was used to evaluate the cytotoxicity of the compounds against human hepatoma HepG2 cells. The experimental design included a blank group, a control group treated with 50 µM cisplatin, and a test group treated with compounds **1**–**7** at 20 µM, with cellular inhibition rates calculated from optical density (OD) measurements in triplicate.

## 3 Results and Discussion

### 3.1 Bioinformatic analysis of the tpm gene cluster

Bioinformatic analysis of the genome of strain LR53 identified an ansamycin biosynthetic gene cluster (*tpm*, Fig. 1a, GenBank accession No. PV541294), which was predicted to possess an unprecedented pentaketide backbone configuration (AHBA-C_3_-C_2_-C_3_-C_3_). The cluster spanned approximately 62 kb and contained 23 open reading frames (ORFs). The predicted functions (Table S1) included multiple genes involved in AHBA biosynthesis (*tpmG*–*N*), three genes encoding type I modular polyketide synthases (*tpmA*–*C*), and an amide synthase (*tpmD*) responsible for cyclization and the release of full-length polyketide chain. Additionally, the cluster harbors four predicted regulatory protein genes (*tpmLAL, tpmSARP, tpm5*, and *tpm7*) as well as five genes (*tpm2, tpm13, tpm15*–*17*) likely involved in redox processes. Disruption of the PKS gene *tpmA* did not alter the metabolite profile (Fig. S2b), indicating that the cluster is either inactive or expressed at extremely low levels during standard laboratory growth.

### 3.2 Targeted discovery of tropansamycins

Previous research has demonstrated that overexpression of pathway-specific positive regulators can often activate “cryptic” ansamycin gene clusters [7,[9], [10], [11]]. Therefore, we overexpressed the candidate regulator genes *tpmLAL* and *tpmSARP* individually and in combination (Fig. S4a). HPLC profiles of the resulting strains LR53LAL, LR53SARP, and LR53LS were indistinguishable from those of the wild type (Fig. S4b), indicating that simple transcriptional activation was ineffective. Given that insufficient precursor supply can limit ansamycin production [22], we next explored precursor-directed activation by supplementing the fermentation medium with exogenous AHBA (200 mg/L). This approach induced two additional peaks (**1** and **2**) with similar UV spectra in LR53 WT and LR53SARP that were absent in the *tpmA*-disrupted mutant LR53ΔPKS (Figs. 1b, S4b, and S7), implicating these peaks as the desired pentaketide ansamycins. Their absence in LR53LAL and LR53LS suggested that TpmLAL may function as a negative regulator; however, complete deletion of *tpmLAL* in LR53 WT did not restore production of **1** and **2** (Fig. S5b), pointing to a more complex regulatory network. Systematic AHBA feeding (50–200 mg/L) resulted in a concentration-dependent increase of **1** and **2** that plateaued at 200 mg/L (Fig. S8). Ultimately, a 10 L fermentation of LR53 WT under these optimized conditions yielded tropansamycins A (**1**) and B (**2**), enabling complete structural characterization.

**Fig. 2:**
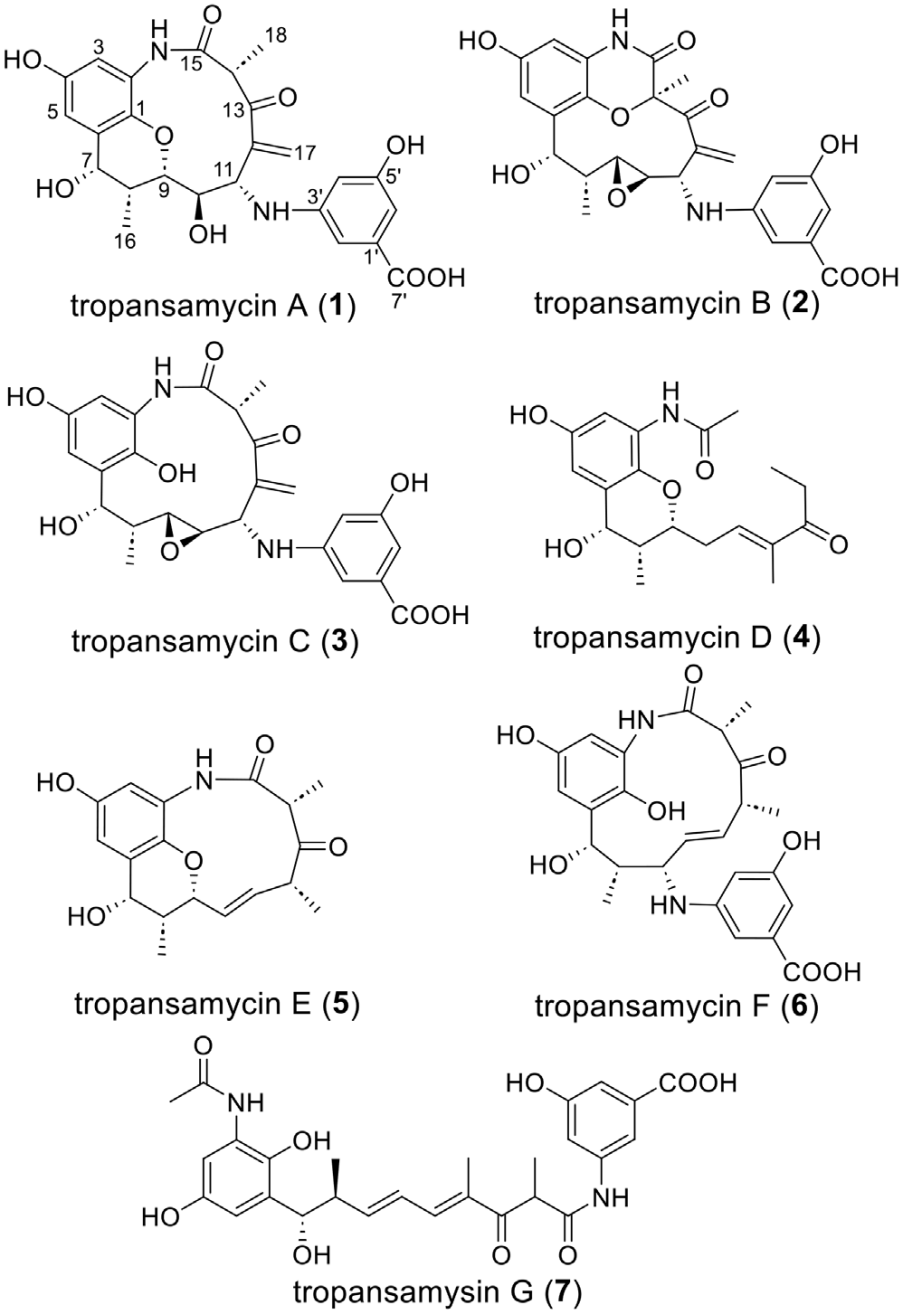
Structures of tropansamycins A–G (**1**–**7**).

Tropansamycin A (**1**, 0.2 mg/L) was isolated as a brown oil. The HRESIMS result was *m/z* 499.1712 [M + H]⁺ (calcd 499.1711) consistent with that of C_25_H_26_N_2_O_9_ (Fig. S9). Comprehensive analysis of 1D and 2D NMR data (Table S4, Figs. S10 and S21-S26) established the planar structure (Fig. 2). The ^13^C, HSQC, and HMBC spectra showed 25 carbon resonances corresponding to 2 CH_3_, 1 CH_2_, 11 CH, and 11 quaternary centers. HMBC correlations from H3 (*δ*_H_ 6.30) to C1 (*δ*_C_ 140.3), C2 (*δ*_C_ 124.5), C4 (*δ*_C_ 151.4), and C5 (*δ*_C_ 112.8), and from H5 (*δ*_H_ 6.77) to C1, C3 (*δ*_C_ 113.1), C4, and C7 (*δ*_C_ 67.5) defined a tetrasubstituted *p*-hydroquinone. A C_9_ aliphatic chain (C7–C15) bearing three methyl branches was deduced from COSY correlations (H7–H11) and HMBC correlations of Me16 to C7, C8, and C9; H17 to C11, C12, and C13; and Me18 to C13, C14, and C15. The hydroquinone and aliphatic fragments were linked via HMBC correlations from H7 to C1 and C6 and from 2-NH (*δ*_H_ 9.12) to C1, C14 (*δ*_C_ 51.2), and C15 (*δ*_C_ 171.9), while HMBC correlation from H9 (*δ*_H_ 3.78) to C1 confirmed an ether bridge between C1 and C9. A second AHBA unit was identified by HMBC correlations of H2’ (*δ*_H_ 6.65) to C6’ (*δ*_C_ 104.7) and C7’ (*δ*_C_ 168.3); H4’ (*δ*_H_ 6.04) to C2’ (*δ*_C_ 106.2), C5’ (*δ*_C_ 158.4), and C6’; and H6’ (*δ*_H_ 6.54) to C7’ and was attached to C11 via its amino group, as demonstrated by the COSY correlation between H11 (*δ*_H_ 4.42–4.40) and 3’-NH (*δ*_H_ 6.01). ROESY correlations established the relative configuration of **1**. Correlations between H-7/H-9/H-11/H-14 indicated these protons are cofacial, whereas those between Me-16/H-10/H-4′ suggested they reside on the opposite face (Fig. S10). Given that the 2-methyl group flanked by carbonyls (C14 in **1**) can undergo keto–enol tautomerism to produce diastereomers [14,[23], [24], [25]], we recorded the ^1^H NMR spectrum of **1** in CD_3_OD to investigate deuterium exchange at the H14 position. The observation of two diastereomers and the absence of the H14 signal, accompanied by the loss of the doublet coupling to Me18 (Figs. S27 and S28), confirmed epimerization mediated by keto-enol tautomerism.

Tropansamycin B (**2**, 0.1 mg/L), a brown oil, showed *m/z* 497.1557 [M + H]⁺ (calcd 497.1555) corresponding to C_25_H_24_N_2_O_9_ (Fig. S9). Its NMR data (Table S5, Figs. S11 and S29–S34) revealed the same core scaffold as **1**, but with an epoxide spanning C9/C10 and an ether linkage between C1 and C14, consistent with the characteristic ^13^C shifts for C9 (*δ*_C_ 57.4), C10 (*δ*_C_ 55.4), and C14 (*δ*_C_ 82.6). ROESY correlations between Me16 and H10 indicated a *cis*-disubstituted epoxide, whereas those between Me18 and H9 indicated they are cofacial. The discovery of tropansamycins A and B (**1** and **2**) suggests that the *tpm* cluster encodes a novel subfamily of pentaketide ansamycins.

### 3.3 Enhanced tropansamycin production via PKS gene overexpression

We constructed the mutant strain LR53OEPKS by inserting the strong kasO* promoter upstream of the PKS gene *tpmA* (Fig. S3) to enhance tropansamycins production for activity studies. HPLC analysis of this strain revealed several new peaks (Fig. 1b) and a modest increase in **1** (0.6 mg/L) and **2** (0.3 mg/L). A 10 L fermentation of LR53OEPKS afforded two additional metabolites, i.e., tropansamycins C (**3**, 0.3 mg/L) and D (**4**, 0.2 mg/L); however, the low titers and chromatographic overlap precluded further purification of minor congeners. Tropansamycin C (**3**), a brown oil, showed *m/z* 499.1711 [M + H]⁺ (calcd 499.1711), matching the formula C_25_H_26_N_2_O_9_ (Fig. S9). The NMR data (Table S6, Figs. S12 and S35–S40) of **3** were similar to those of **2**, except the cleavage of the C1–C14 ether bridge, as evidenced by the upfield shift of C14 (*δ*_C_ 43.7). Notably, **3** undergoes transformation to **1** in heated water via a non-enzymatic process (Fig. S13), suggesting that the relative configurations at C7, C8, C10, C11, and C14 are the same as those in **1**. Tropansamycin D (**4**) was obtained as a colorless powder. HRESIMS established C_19_H_25_NO_5_ (Fig. S9, *m/z* 348.1808 [M + H]⁺; calcd 348.1805). NMR analysis (Table S7, Figs. S14 and S41–S45) revealed a truncated ansamycin skeleton matching the C1–C13 segment of **1**, but lacking the C10 hydroxyl and bearing a C11/C12 double bond, as indicated by C10 (*δ*_C_ 32.3), C11 (*δ*_C_ 138.8), and C12 (*δ*_C_ 137.7) and HMBC correlations of H11 to C9 and C10 and Me17 to C11, C12, and C13. A terminal propionyl unit (C13–C15) was identified through HMBC correlations from Me15 to C13(*δ*_C_ 202.1) and C14 (*δ*_C_ 30.3) and COSY correlations between H14 and Me15. Finally, 2-NH showed HMBC correlations to C1, C2, C3, and an acyl carbonyl at C1’ (*δ*_C_ 168.4), together with HMBC correlations from Me2’ to C1’, confirming *N*-acetylation. The relative upfield chemical shift of the allylic methyl carbon (Me17, *δ*_C_ 11.9) indicated an *E* configuration of the C11/C12 double bond [26].

### 3.4 CYP450 enzyme Tpm16 mediates tropansamycin oxidative tailoring

We individually deleted two candidate genes, the CYP450 gene *tpm16* and the FAD-dependent oxidoreductase gene *tpm17*, in strain LR53OEPKS (Fig. S6a) to investigate the post-PKS oxidative tailoring steps. HPLC analysis revealed that deletion of *tpm16* completely abolished the formation of **1**–**4** and accumulated several new metabolites (Fig. 1b), while deletion of *tpm17* had no discernible effect (Fig. S6b). The results revealed that the CYP450 Tpm16 is required for the oxidative maturation of tropansamycins. A 10 L culture of LR53OEPKSΔtpm16 provided three new products (**5**–**7**).

**Fig. 3:**
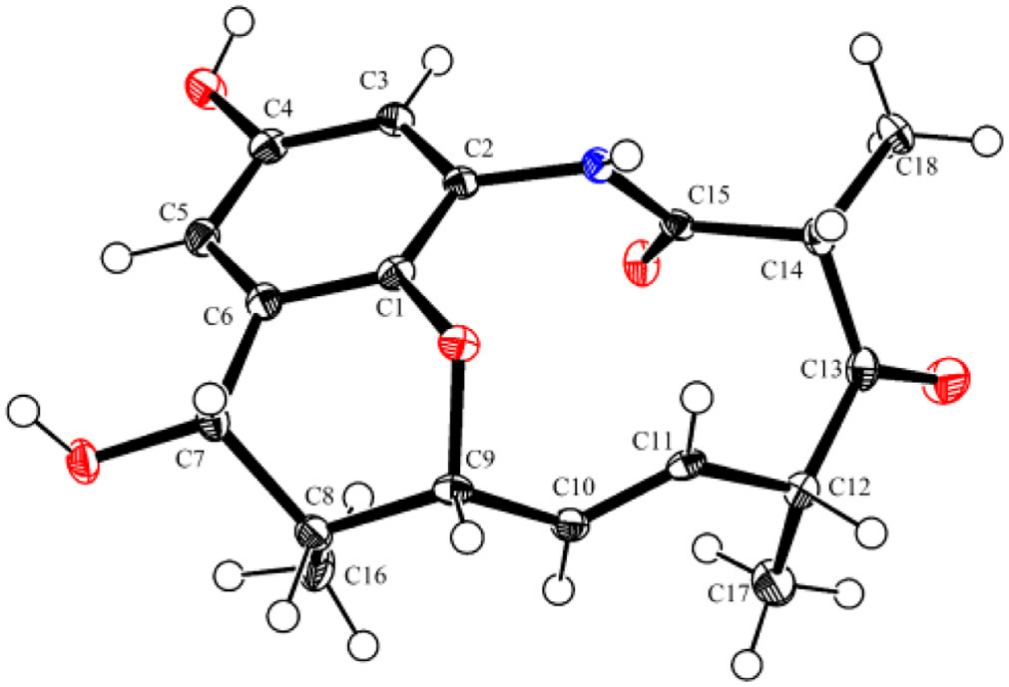
X-ray crystal of structure of **5**.

Tropansamycin E (**5**) crystallized from MeOH as colorless needles. HRESIMS provided *m/z* 332.1494 [M + H]⁺ (calcd 332.1492) consistent with C_18_H_21_NO_5_ (Fig. S9). Its NMR data (Table S8, Figs. S15 and S46−S51) closely paralleled those of **1**, but with two key differences: the C10 hydroxyl and attached AHBA unit were replaced by a C10 (*δ*_C_ 127.9)/C11 (*δ*_C_ 131.1) double bond and the C12/C17 double bond in **1** was completely reduced. The *E* configuration of the Δ^10, 11^double bond was assigned by a large coupling constant (*J* = 15.6). ROESY correlations H7/H9, Me16/H10/Me17, and H11/H14/H12 placed H7, H8, H9, H12 and H14 on one side of the macrolactam and Me16, Me17, and Me18 on the opposite side. Finally, single-crystal X-ray diffraction (Cu Kα, CCDC 2414079, Fig. 3 and Table S9) established the absolute configuration of **5** as *7S, 8R, 9R, 12R, 14R*. Accordingly, the absolute configurations of **1**−**4** were determined (Fig. 2). For **1**, ROESY correlations Me16/H10/H4’ and H7/H9/H11/H14 support a *10R, 11S* configuration (Fig. S10). Analogous ROESY correlations H10/Me16 assign *9R, 10R* in **2** and **3**, while correlations H9/Me18 suggest a *14R* configuration (Figs. S11 and S12).

Tropansamycin F (**6**) was obtained as a brown oil. HRESIMS showed *m/z* 485.1917 [M + H]⁺ (calcd 485.1918), establishing the formula C_25_H_28_N_2_O_8_ (Fig. S9). Detailed NMR analysis (Table S10, Figs. S16 and S52−S57) revealed that **6** differs from **5** by acquisition of an extra AHBA unit at C9 and cleavage of the C1–C9 ether bridge (Fig. 2). A large coupling constant between H10 and H11 (*J* = 16.1 Hz) confirmed an *E* geometry for the Δ^10,11^ olefin, while ROESY correlations of H2’/H17/H4’ suggest a *9S* configuration. Tropansamycin G (**7**) was obtained as a brown oil. HRESIMS revealed an [M + H]⁺ ion at *m/z* 527.2026 (calcd 527.2024) for C_27_H_30_N_2_O_9_ (Fig. S9). Analysis of the 1D NMR (DMSO‑*d*_6_) data for **7** (Table S11, Figs. S17 and S58−S63) disclosed two sets of closely overlapping resonances, indicative of an approximately 1:1 mixture of epimers at the chiral center C14, similar to that of compound **1**. The NMR data resembled a complete *ansa* chain (C1–C15), as demonstrated by HMBC correlations from Me18 to C13, C14, and C15, and introduced an additional Δ^9,10^ olefin (*δ*_C_ 147.4/126.2). An extra AHBA unit is attached to C15 via an amide linkage, supported by the HMBC correlations from 3’-NH to C15, C2’, C3’, and C4’. The *E* configurations of the Δ^9, 10^ and Δ^11, 12^ double bonds were deduced from a large ^3^*J*_H9/H10_ value (15.2 Hz) and the characteristic upfield shift of Me17 (*δ*_C_ 11.8), respectively (Fig. 2). Thus, tropansamycin G (**7**) was characterized as an equilibrium mixture of C14 epimers.

### 3.5 Biological activity of **1**–**7**

Antimicrobial activity screening of compounds **1**–**7** revealed potent antibacterial properties for compounds **5** and **6** against gram-negative plant-pathogenic bacteria *Xanthomonas oryzae* pathovars and gram-positive *Staphylococcus aureus*. Using disk diffusion and broth microdilution assays, we observed clear inhibition zones (Fig. S18) and determined MICs ranging from 2–8 µg/mL (Table 1). Intriguingly, compounds **5** and **6** also exhibited inhibitory activity against the producing *Streptomyces* strain cultivated on LB agar medium, but not on ISP3 agar medium (Fig. S18). These findings suggest that Tpm16 catalyzes a modification step that attenuates the bioactivity of these compounds and that the producer may employ multiple self-resistance mechanisms. Cytotoxicity assessments demonstrated that all tested compounds showed no significant toxicity towards human hepatocellular carcinoma HepG2 cells at concentrations up to 20 µM (Table S12), indicating promising safety potential for potential agricultural or pharmaceutical development.

**Table 1:** Antimicrobial activities of **5** and **6**.

	MIC (µg/mL)		
**Strains tested**	**5**	**6**	Positive control
*X. oryzae* pv. *oryzae*	4–8	2–4	2–4
*X. oryzae* pv. *oryzicola*	4–8	4–8	1–2
*P. aeruginosa* PAO1	>64	>64	2–4
*S. aureus* ATCC 25923	4–8	2–4	1–2
*B. subtilis*	32–64	16–32	1–2
*M. smegmatis* MC^2^ 155	>64	>64	1–2
*S.* sp. LR53OEPKS	16–32	16–32	<1
*C. albicans* 5314	>64	>64	4–8
*C. gloeosporioides* lq27	32–64	32–64	4–8
*A. niger*	>64	>64	6–18
*A. flavus* ATCC 32636	>64	>64	6–18

### 3.6 Biosynthetic pathway of tropansamycins

**Fig. 4:**
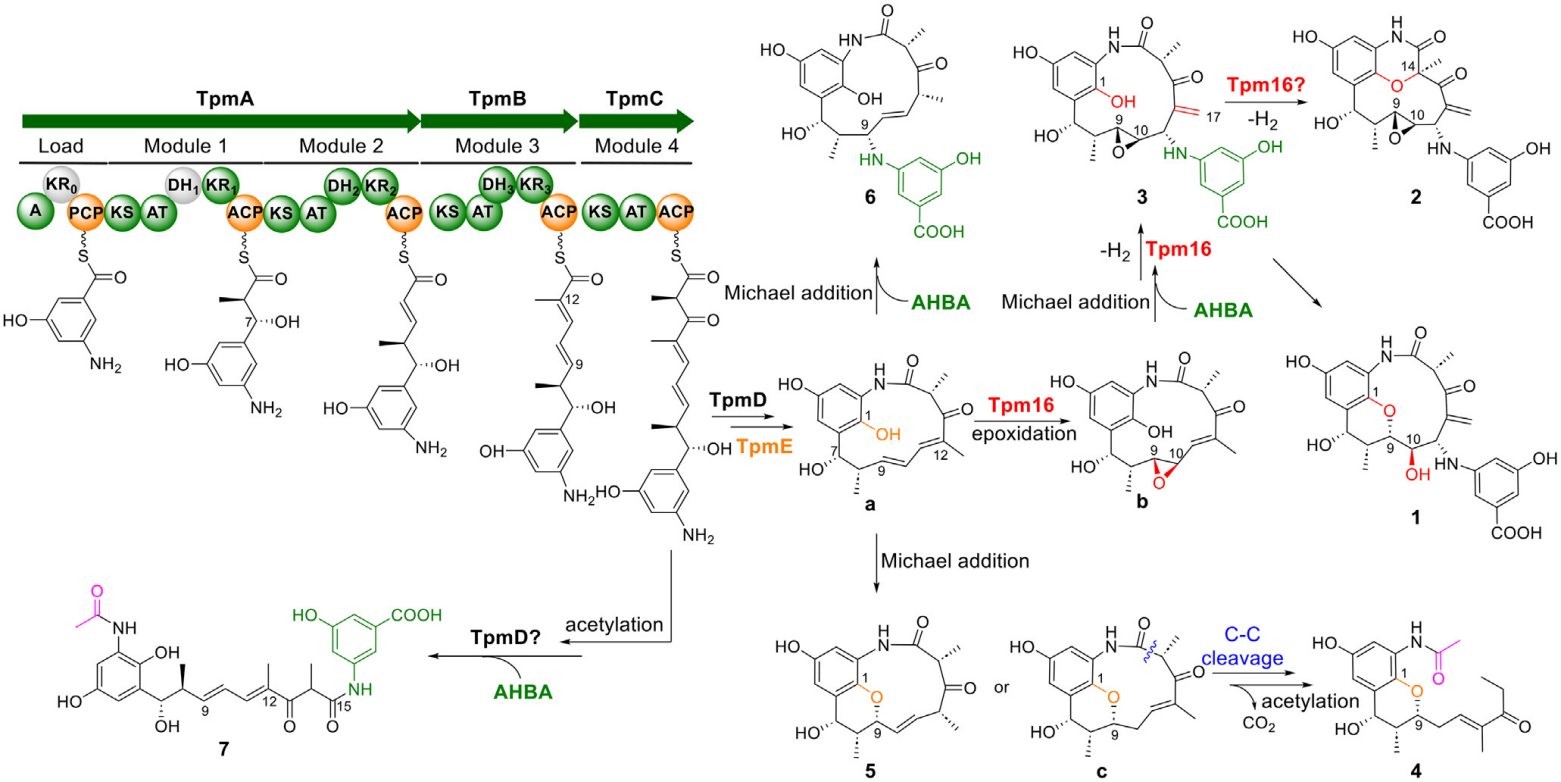
Proposed biosynthetic pathway of tropansamycins.

The biosynthetic pathway for tropansamycins was proposed (Fig. 4). The PKSs TpmA–C utilize AHBA as the starter unit, along with one malonyl-CoA and three methylmalonyl-CoA as extender units, to construct the pentaketide backbone. Sequence alignment of the ketoreductase (KR) domains indicates that KR_0_ is inactive due to the absence of the NADPH-binding motif (GXGXXG) and crucial catalytic triad residues (Fig. S19a), while KR_1_–KR_3_ are confirmed as active and classified as B1-type KRs (Fig. S19b) [27], consistent with the presence of two *trans* double bonds located at C9/C10 and C11/C12 in **7**. Further analysis of dehydratase (DH) domains shows that DH_1_ is inactive, lacking three conserved motifs (Fig. S20), which is consistent with the presence of a hydroxyl group at C7 [28]. The hydroxylase TpmE shares 49.7% amino acid sequence identity with the hydroxylase Nam7 from the neoansamycin biosynthetic pathway [29] and is predicted to catalyze the same reaction. The amide synthase TpmD cyclizes the full-length polyketide chain, after which TpmE installs the C1-OH to furnish precursor **a**. In the absence of CYP450 enzyme Tpm16, intermediate **a** may undergo either a Michael addition to form the C1–O–C9 ether bridge, yielding **5** and **c**, or functionalization at C9 with an additional AHBA unit to afford **6**. Compound **c** then undergoes C-C cleavage and subsequent *N*-acetylation to produce **4**. Conversely, in the presence of Tpm16, compound **a** is epoxidized at the C9/C10 double bond to form intermediate **b**, as demonstrated for CYP450 PimD in pimaricin biosynthesis [30]. This intermediate can further undergo both Michael addition and Tpm16-mediated dehydrogenation, ultimately yielding **3**. Additionally, compound **3** may undergo oxidative cyclization catalyzed by Tpm16 to yield **2** as reported for CYP450 GsfF in griseophenone B biosynthesis [31]. The phenolic coupling proceeds through initial phenolic O-H abstraction, followed by direct attack at the C14 position by the enol form of the C13 carbonyl group to form the morpholinone ring. Notably, the mature linear polyketide chain may be acetylated at the 2-NH position and potentially released by TpmD, utilizing another AHBA unit to generate **7**.

## 4 Conclusions

In this study, genome sequencing and bioinformatic analysis enabled the identification of the *tpm* pentaketide ansamycin gene cluster in *Streptomyces* sp. LR53. Through a combination of AHBA precursor supplementation, promoter insertion, and targeted gene deletion, we discovered and characterized seven novel ansamycins, tropansamycins A–G (**1**–**7**). A distinguishing feature of tropansamycins from other reported pentaketide ansamycins is the unique arrangement of extender units (C_3_-C_2_-C_3_-C_3_) during polyketide chain elongation, along with distinct post-PKS modifications. Among structurally related compounds, cebulactam A1 most closely resembles tropansamycin E (**5**), differing only in the absence of a C10 methyl group and the stereochemistry of the *ansa* bridge [16]; however, its bioactivity has not been reported. While ansamycins are well-known for their anti-tuberculosis and antitumor activities, their efficacy against gram-negative bacteria remains underexplored. Notably, compounds **5** and **6**, isolated from a Δtpm16 (CYP450) mutant, exhibited potent antibacterial activity against gram-negative plant-pathogenic *Xanthomonas oryzae* pathovars with MIC values of 2–8 µg/mL, indicating their potential as novel pesticide candidates. The CYP450 enzyme Tpm16 was observed to play a crucial role in modulating the bioactivity of pathway intermediates. Collectively, this study expands the structural and bioactivity repertoire of pentaketide ansamycins.

## Data availability statement

The biosynthetic gene cluster of *tpm* has been deposited into GenBank database under Accession No. PV541294. Crystallographic data of tropansamycin E (**5**) has been deposited with the Cambridge Crystallographic Data Centre (CCDC) under deposition number CCDC 2414079. All relevant data supporting the findings of this study are provided within the manuscript and the Supplementary Materials.

## CRediT authorship contribution statement

**Haotian Wang:** Writing – original draft, Investigation, Data curation. **Run Jiao:** Data curation. **Liran Ma:** Investigation. **Yaoyao Li:** Writing – review & editing, Data curation. **Yuemao Shen:** Writing – review & editing, Supervision, Project administration. **Haoxin Wang:** Writing – review & editing, Supervision, Project administration, Funding acquisition.

## Declaration of competing interest

The authors declare that they have no known competing financial interests or personal relationships that could have appeared to influence the work reported in this paper.

## Appendix Supplementary materials

Image, application 1
