# Supplementary material for: Genome-guided discovery of tropansamycins: antimicrobial pentaketide ansamycins
Source: Eng Microbiol. 2026 Feb 12;6(2):100263. doi: 10.1016/j.engmic.2026.100263 (PMC13000494; doi:10.1016/j.engmic.2026.100263)
Supplement: Supplementary file 1 [file mmc1.pdf]

Supplementary Materials

Genome-guided discovery of tropansamycins: antimicrobial pentaketide ansamycins

Haotian Wang[a], Run Jiao[b], Liran Ma[b], Yaoyao Li[b], Yuemao Shen[a,b,*], and Haoxin Wang[a,*]

[a]State Key Laboratory of Microbial Technology, Shandong University, Qingdao 266237, China

[b]Key Laboratory of Chemical Biology of Ministry of Education, School of Pharmaceutical Sciences, Cheeloo College of Medicine, Shandong University, Jinan 250012, China

Methods

Strains, primers, plasmids, and culture conditions

Streptomyces sp. LR53 was isolated from the soil collected at Xishuangbanna Tropical Botanical Garden, Yunnan, China. The strain was cultured on YMG agar medium (0.4% yeast extract, 1% malt extract, 0.4% glucose, 1.5% agar, pH 7.4) at 30°C to promote sporulation and on ISP3 agar medium (2% oatmeal, 2% agar, pH 7.2) for fermentation. *E. coli* DH5α was used as the host for general DNA cloning and sequencing, while *E. coli* ET12567/pUZ8002 served as the conjugation donor strain. Intergeneric conjugation between ET12567/pUZ8002 and strain LR53 was conducted on SFM agar medium (2% D-mannitol, 2% soybean meal, 2% agar, pH 7.4) supplemented with 20 mM $MgCl_2$. Detailed information about the strains, plasmids, and primers are provided in Table S1.

General experimental procedures for isolation and characterization of compounds

Optical rotations were measured using an Anton Paar MCP200 polarimeter. NMR spectra were measured using a Bruker AVANCE NEO 600 MHz spectrometer with DMSO-d_6 as the solvent. X-ray diffraction data were collected using a Bruker XtaLAB Synergy diffractometer. High-resolution electrospray ionization mass spectrometry (HRESIMS) data were collected using a Bruker impact HD instrument. HPLC analysis was performed using an Agilent ZORBAX Eclipse SB-C18 column (5 µm, 4.6 × 250 mm) at a flow rate of 1 mL/min. Semipreparative HPLC was carried out using an Agilent ZORBAX Eclipse SB-C18 column (5 µm, 9.4 × 250 mm) at a flow rate of 4 mL/min. Both analyses were conducted on a DIONEX Ultimate 3000 instrument, with detection at UV 235 nm. Water with 0.1% formic acid (solvent A) and acetonitrile with 0.1% formic acid (solvent B) were used as mobile phases. Sephadex LH-20 (25–100 µm, Pharmacia Biotech, Denmark) and LiChroprep RP-18 (40–63 µm; Merck, Germany) were used for column chromatography. All solvents used were of analytical grade.

The characteristics of 1−7

*Tropansamycin A (**1**)*. brown oil; $[\alpha]_D^{20}$ = +88.0 (*c* = 0.3, MeOH); UV/Vis: λ_{max} 213, 305 nm (Fig. S7); HRESIMS m/z 499.1712 $[M + H]^+$ (calcd for $C_{25}H_{27}N_2O_9^+$, 499.1711). ^1H and ^{13}C NMR, Table S4.

*Tropansamycin B (**2**)*. brown oil; $[\alpha]_D^{20}$ = -21.0 (*c* = 0.3, MeOH); UV/Vis: λ_{max} 217, 310 nm (Fig. S7); HRESIMS m/z 497.1557 $[M + H]^+$ (calcd for $C_{25}H_{25}N_2O_9^+$, 497.1555). ^1H and ^{13}C NMR, Table S5.

*Tropansamycin C (**3**)*. brown oil; $[\alpha]_D^{20}$ = +56.9 (*c* = 0.3, MeOH); UV/Vis: λ_{max} 209, 306 nm (Fig. S7); HRESIMS m/z 499.1711 $[M + H]^+$ (calcd for $C_{25}H_{27}N_2O_9^+$, 499.1711). ^1H and ^{13}C NMR, Table S6.

*Tropansamycin D (**4**)*. colorless powder; $[\alpha]_D^{20}$ = -24.8 (*c* = 0.3, MeOH); UV/Vis: λ_{max} 222, 308 (Fig. S7) nm; HRESIMS m/z 348.1808 $[M + H]^+$ (calcd for $C_{19}H_{26}NO_5^+$, 348.1805). ^1H and ^{13}C NMR, Table S7.

*Tropansamycin E (**5**)*. colorless crystal; $[\alpha]_D^{20}$ = +147.7 (*c* = 0.3, MeOH); UV/Vis: λ_{max} 206, 291 (Fig. S7) nm; HRESIMS m/z 332.1494 $[M + H]^+$ (calcd for $C_{18}H_{22}NO_5^+$, 332.1492). ^1H and ^{13}C NMR, Table S8. Crystallographic data, Table S9.

*Tropansamycin F (**6**)*. brown oil; $[\alpha]_D^{20}$ = -85.7 (*c* = 0.3, MeOH); UV/Vis: λ_{max} 238, 304, 342 (Fig. S7) nm; HRESIMS m/z 485.1917 $[M + H]^+$ (calcd for $C_{25}H_{29}NO_8^+$, 485.1918). ^1H and ^{13}C NMR, Table S10.

*Tropansamycin G (**7**)*. brown oil; $[\alpha]_D^{20}$ = -16.7 (*c* = 0.3, MeOH); UV/Vis: λ_{max} 213, 293 (Fig. S7) nm; HRESIMS m/z 527.2026 $[M + H]^+$ (calcd for $C_{27}H_{31}N_2O_9^+$, 527.2024). ^1H and ^{13}C NMR, Table S11.

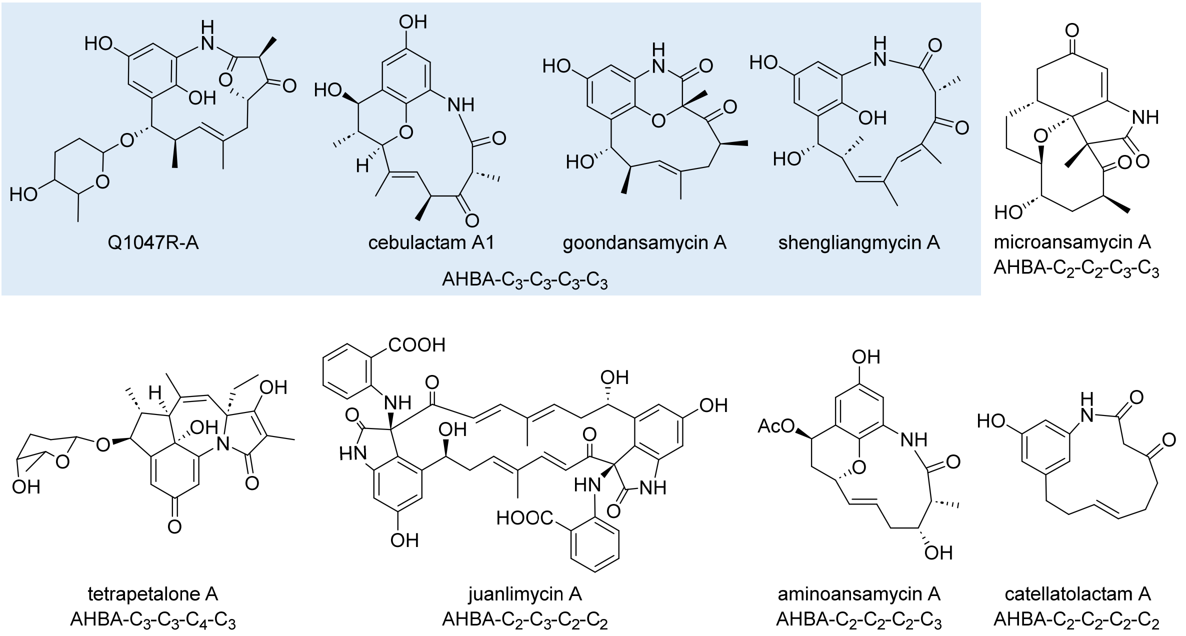

Figure S1. Known scaffolds of pentaketide ansamycins.

Table S1. The genetic organization of the *tpm* gene cluster in *Streptomyces* sp. LR53.

ORF	AA	Proposed function	Identity/similarity (%)	
tpm1	316	ScbA/BarX family γ-butyrolactone biosynthesis protein	100/100	WP_205372931.1
tpm2	323	NAD-dependent epimerase/dehydratase family protein	99/99	WP_205372932.1
tpmLAL	941	LuxR family transcriptional regulator	99/99	WP_205372933.1
tpmSARP	294	SARP family transcriptional regulator	99/100	WP_280521549.1
tpm3	223	DUF4097 family β strand repeat-containing protein	98/99	WP_205372935.1
tpm4	316	class A beta-lactamase	99/99	WP_205372936.1
tpm5	382	LysR family transcriptional regulator	95/95	MBM9618692.1
tpm6	300	serine hydrolase	99/99	WP_205372938.1
tpmN	316	kinase	99/99	WP_205372939.1
tpm7	129	helix-turn-helix transcriptional regulator	99/99	MBM9618695.1
tpm8	251	alpha/beta fold hydrolase	100/100	WP_205372941.1
tpmJ	160	aminoDHQ dehydratase	100/100	WP_147986149.1
tpmK	389	AHBA synthase	99/100	WP_205372942.1
tpmL	389	oxidoreductase	99/100	WP_205376435.1
tpmM	237	phosphatase	99/100	WP_205372943.1
tpm9	144	VOC family protein	99/98	WP_205372944.1
tpmG	356	aminoDHQ synthase	99/99	WP_205372945.1
tpm10	280	hypothetical protein	99/98	WP_205372946.1
tpmA	4546	type I polyketide synthase<br>(CAL-KR-T, KS-AT-DH-KR-T, KS-AT-DH-KR-T)	98/98	WP_244883140.1
tpmB	1785	type I polyketide synthase (KS-AT-DH-KR-T)	99/98	WP_205372947.1
tpmC	1047	type I polyketide synthase (KS-AT-T)	99/99	WP_205372948.1
tpmD	259	amide synthase	99/99	WP_244882681.1
tpmE	545	3-(3-hydroxy-phenyl) propionate hydroxylase	99/99	WP_205372949.1
tpm11	147	transcriptional regulator	100/100	WP_205372950.1
tpm12	499	MFS transporter	99/100	WP_205376438.1
tpm13	369	Gfo/Idh/MocA family oxidoreductase	99/99	WP_244882683.1
tpm14	264	DUF2306 domain-containing protein	99/99	WP_205372951.1
tpm15	73	ferredoxin	100/100	WP_205372952.1
tpm16	420	cytochrome P450	99/99	WP_244882685.1
tpm17	500	FAD-binding protein	99/99	WP_205372953.1
tpm18	424	acyltransferase	99/98	WP_244882688.1
tpmH	463	aminoDAHP synthase	99/99	WP_205372954.1
tpmI	301	shikimate dehydrogenase	98/98	WP_205372955.1
tpm19	149	SPW repeat protein	100/100	WP_205372956.1
tpm20	515	MFS transporter	99/99	MBM9618720.1
tpm21	132	RidA family protein	100/100	WP_205372958.1
tpm22	379	acyl-CoA dehydrogenase family protein	99/100	WP_205372959.1
tpm23	538	AMP-binding protein	99/99	WP_205372960.1

Table S2. Strains and plasmids used in this study.

Strains/Plasmids	Relevant characteristic	Source
Streptomyces		
LR53 WT	*Streptomyces* sp. LR53 wild type	This study
LR53LAL	Recombinant strain with integrated pSET5035-LAL	This study
LR53SARP	Recombinant strain with integrated pSET5035-SARP	This study
LR53LS	Recombinant strain with integrated pSET5035-LS	This study
LR53ΔPKS	The *tpmA* gene disrupted mutant of LR53	This study
LR53ΔLAL	The *tpmLAL* gene deleted mutant of LR53	This study
LR53OEPKS	The *tpmPKS* gene overexpression mutant of LR53	This study
LR53OEPKSΔtpm16	The *tpm16* gene deleted mutant of LR53OEPKS	This study
LR53OEPKSΔtpm17	The *tpm17* gene deleted mutant of LR53OEPKS	This study
E. coli		
DH5α	Strain used for general DNA cloning and sequencing	Invitrogen
ET12567/pUZ8002	Strain used for *E. coli-Streptomyces* intergeneric conjugation	[1]
**Plasmids**		
pSET5035	pSET152 derivative, compatible with BioBrick assembly standard, *aac(3)IV*	[2]
pSET5035-KE	pSET5035 derivative with convergent *kasO*p* and *ermE*p* promoters, compatible with BioBrick standards	This study
pSET5035-LAL	The *tpmLAL* gene overexpression plasmid	This study
pSET5035-SARP	The *tpmSARP* gene overexpression plasmid	This study
pSET5035-LS	The *tpmLAL* and *tpmSARP* genes co-overexpression plasmid	This study
pOJ260	A suicide plasmid in *Streptomyces*, *aac(3)IV*, rep^{puc}, *oriT*	[3]
pSPRm	pOJ260 derivative, which bears a melanin biosynthetic gene cassette (*melC1*-*melC2*) under the control of the *ermE*p* promoter, *aac(3)IV*	This study
pSPRm-ΔLAL	Plasmid for the *tpmLAL* gene deletion	This study
pOJ260-ΔPKS	Plasmid for the *tpmA* gene deletion	This study
pSPRm-OEPKS	Plasmid for the *tpmA* gene promoter replacement	This study
pSPRm-Δ*tpm16*	Plasmid for the *tpm16* gene deletion	This study
pSPRm-Δ*tpm17*	Plasmid for the *tpm17* gene deletion	This study

Table S3. Primers used in this study.

Primers	5' - 3'
EmelC-F	ATTGGCGCGCCAGATCTCGATCGTCTAGAAGCTTCGAAGTGCACGCGG TCGATCTTGAC
EmelC-R	CAGCTATGACATGATTACGAATTCTCAGTCGGTGTCGAAGG
POJ260m-F	GAATTCGTAATCATGTCATAGCTGTTTCC
POJ260m-R	TCGAGATCTGGCGCGCCAATTGCATGCCATGGCTGCAGTCATGGCTCTG CCCTCGGG
**Overexpression and deletion of regulatory genes**	
kas-F	ACGAATTCCCAATTGCCTCTAGACTCGAGTGTTCACATTCGAACGG
kas-R	CGACCAAAGGAGGCGGACATATGTCCG
erm-F	CGACCAAAGGAGGCGGACATATGTCCG
erm-R	TTCTGCAGGGATGCATGACTAGTGCACGCGGTCGATCTTG
LAL-F	ACCAAAGGAGGCGGACATATGGAACTGGTCGAACGCGCC
LAL-R	GCAGGGATGCATGACTAGTTCATGCCTGCCCTCCGGCGT
SARP-F	AGGAGGCGGACATATGCGATTCAATTTATTGGGCCC
SARP-R	GGATGCATGACTAGTTCAGCGCCGCCCGTGCGTGG
ver-F	AGGACGGCACGGAAGACGTA
verLAL-R	AGGAGTTCCGCGGCCCGGA
verSARP-R	TTATAGTCCTGTCGGGTTTCG
verLS-R	TTCGCCGAGGATCTCGTGGT
ΔLALup-F	CACACTAGTCCATGGGGTTCGGACCATGCAGCACA
ΔLALup-R	TTCCAATTGGCCGCCAGGCGATTCGTTTT
ΔLALdn-F	GGCCAATTGGAAGCGGACCGCCCCACCCTGT
ΔLALdn-R	GATAAGCTTACCTTCCGCACCTCCGCACCG
verΔLALout-F	GCAGTTGTGCCGTCACCTCG
verΔLALout-R	GAATCGCACGCTGGCTCACT
verΔLALin-F	GCCGTACTGGACACCGCGA
verΔLALin-R	GAGCTGGCGGACGAGGTCCA
**Disruption and overexpression of the PKS genes**	
ΔPKS-F	GACCTGCATAGATCTGTGCTGGGGGCGGACCGCTC
ΔPKS-R	CATGATTACGAATTCGCTCCGCGCCCTCCAGCACC
verΔPKS-F	GACCTGCATAGATCTATGCTGCGAACCGAGCTGAT
verΔPKS-R	CCTACAGCGTGAGCTATGAG
OEPKSup-F	ACCGCGTGCACTTCGAAGCTTTCGGCGCGCGCTCCACAAGG
OEPKSup-R	ACTCGAGTCTAGAGGCAATTGGGAATTCGTCCACACACCCCTCGATAA
OEPKSdn-F	ACCAAAGGAGGCGGACATATGCTGCGAACCGAGCTGAT
OEPKSdn-R	GAGCCATGACTGCAGCCATGGCCCTCAAGACCGAAGGTGTACG
verOEPKS-F	CGGTCGCGGCGAGAATCTTG
verOEPKS-R	GCGCAGGACGGAGAGGTAAC
**Deletion of oxidoreductase genes**	
Δtpm16up-F	CCGCGTGCACTTCGAAGCTTGAGATCAGCGGAGCCAACATC
Δtpm16up-R	GACGCAATTGGAGGAGGTGCCGTGGAAGTCC
Δtpm16dn-F	CCTCCAATTGCGTCATCGTCGGGTCGCCTCT
Δtpm16dn-R	GAGCCATGACTGCAGCCATGGCCCATGCACGGCAACACCAA
verΔtpm16-F	GGTTCGGTCGTCGTGATCGTC
verΔtpm16-R	GACCCGCTACCTGAGCTTCGTC
Δtpm17up-F	ACCGCGTGCACTTCGAAGCTTTCGCGGACGACTACGAGATCA
Δtpm17up-R	GCTCCAATTGCCGTACCGCTTCTTCGACCAC
Δtpm17dn-F	TACGGCAATTGGAGCATGGTTGCTTCCTCCG
Δtpm17dn-R	GAGCCATGACTGCAGCCATGGCTTCGCCGACGAGATCAGCC
verΔtpm17-F	TGAGGCGGTCCAGCAGTTCG
verΔtpm17-R	ATGCCCGTGAGGGACGGAAG

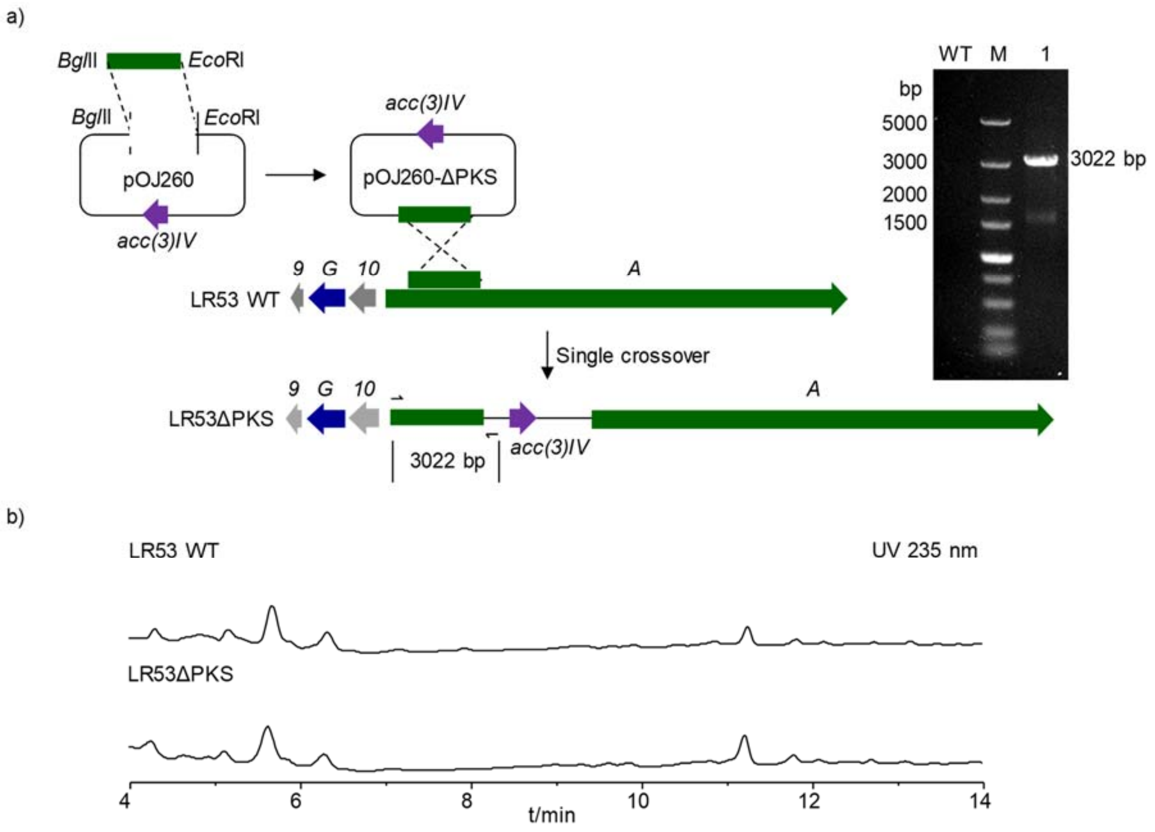

Figure S2. Construction of *tpmA* disruption mutant and HPLC metabolite profiling. a) Construction of the *tpmA* gene disrupted mutant LR53ΔPKS through homologous recombination and PCR verification of the mutant. WT, LR53 wild type; M, DNA marker (DL5000); 1, the mutant LR53ΔPKS (3022 bp expected). b) HPLC analysis of the metabolites produced by the mutant LR53ΔPKS.

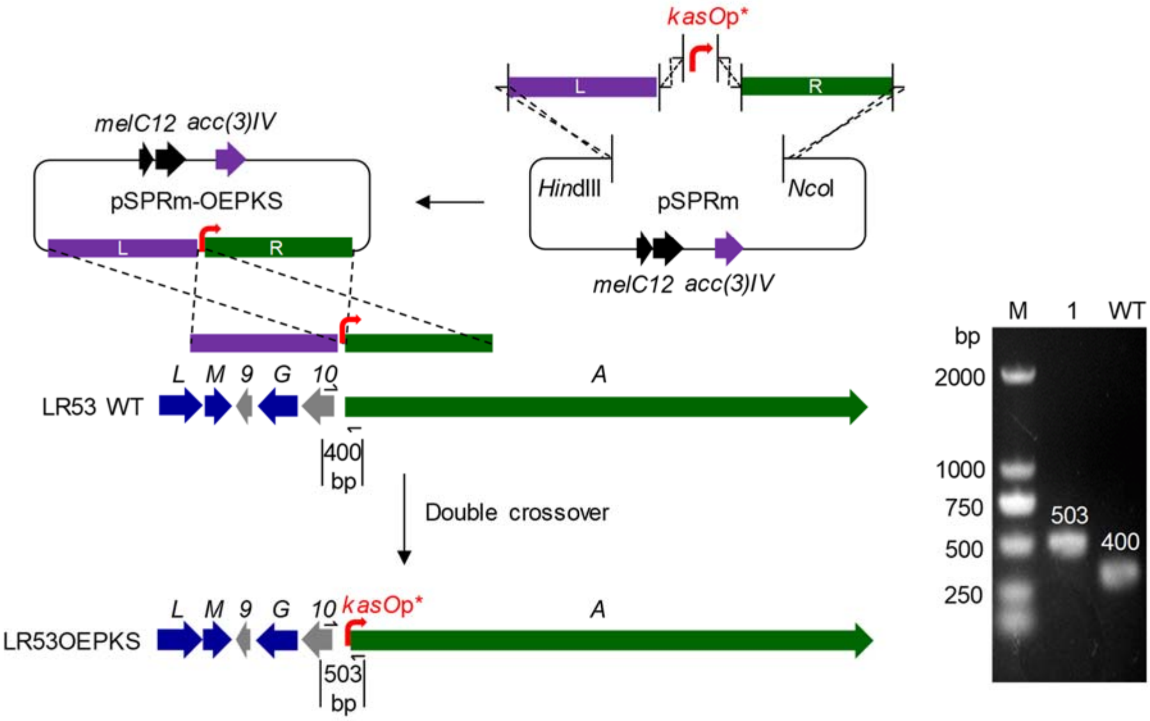

Figure S3. Construction and verification of the LR53OEPKS mutant. Construction of the *tmpA* gene overexpression mutant LR53OEPKS through homologous recombination and PCR verification of the mutant. M, DNA marker (DL2000); 1, the mutant LR53OEPKS (503 bp expected); WT, LR53 wild type (400 bp expected).

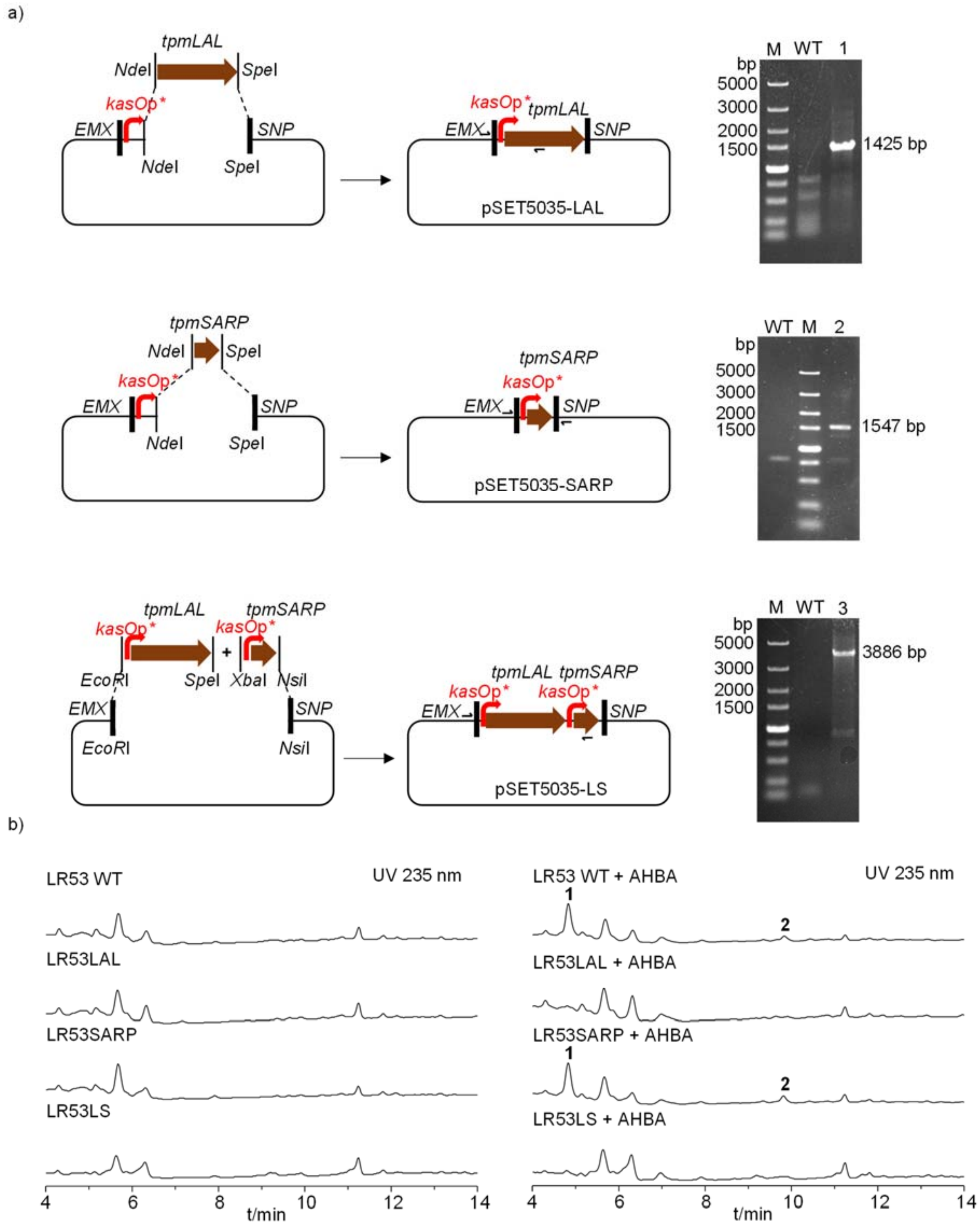

Figure S4. Construction of regulatory genes overexpression mutants and HPLC metabolite profiling. a) Construction of overexpression plasmids for the regulatory genes *tpmLAL* and *tpmSARP,* and PCR verification of the mutants. M, DNA marker (DL5000); WT, LR53 wild type; 1, the mutant LR53LAL (1425 bp expected); 2, the mutant LR53SARP (1547 bp expected); 3, the mutant LR53LS (3886 bp expected). EMX: *Eco*RI-*Mfe*I-*Xba*I restriction sites. SNP: *Spe*I-*Nsi*I-*Pst*I restriction sites. b) HPLC analysis of the metabolites produced by the mutants cultured with or without AHBA (200 mg/L).

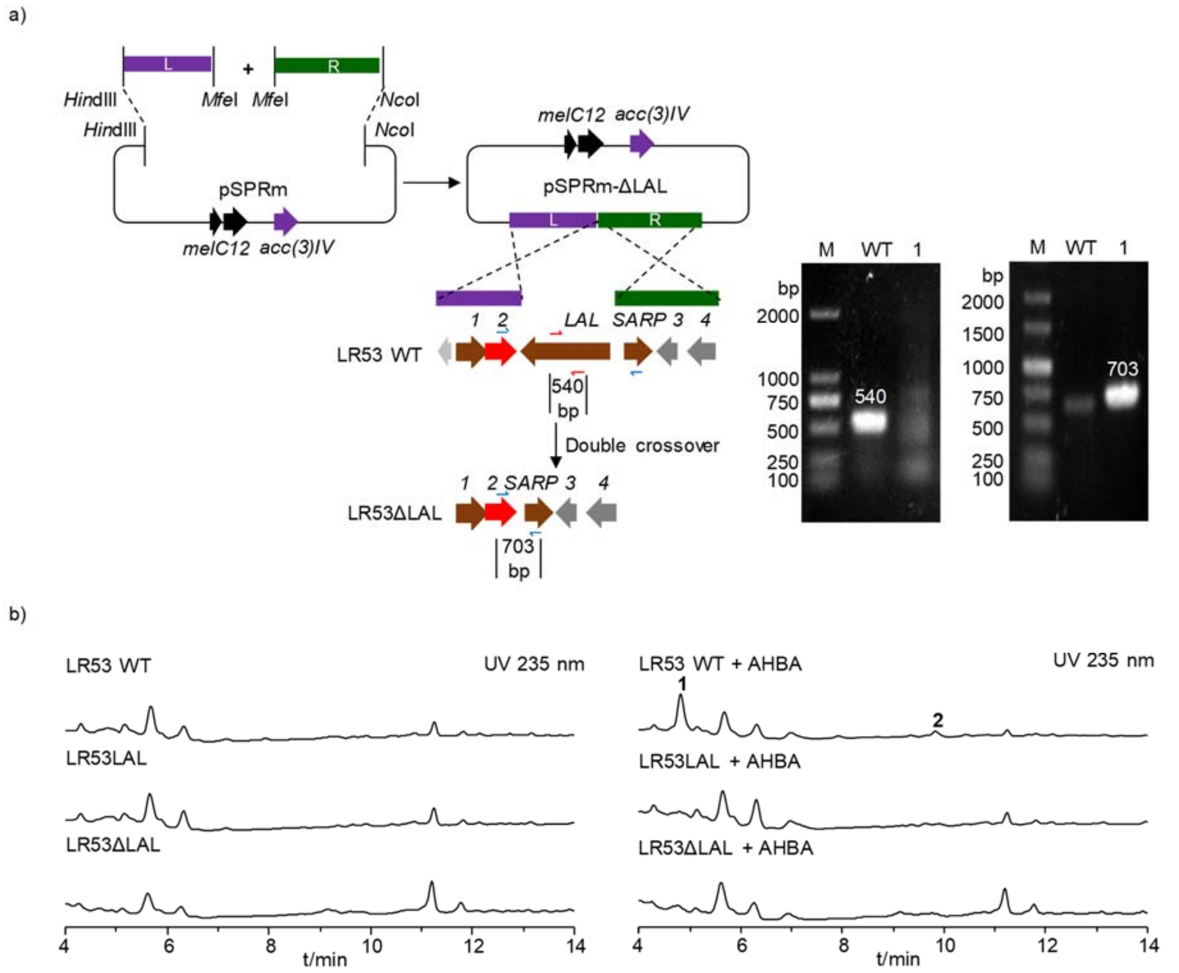

Figure S5. Construction of LR53ΔLAL and HPLC metabolite profiling. a) Construction of the *tpmLAL* gene deletion mutant LR53ΔLAL through homologous recombination, and PCR verification of the mutant. M, DNA marker (DL2000); WT, LR53 wild type (540 bp expected with the primers within the *tpmLAL* gene); 1, the mutant LR53ΔLAL (703 bp expected with the primers outside the *tpmLAL* gene). b) HPLC analysis of the metabolites produced by the mutants cultured with or without AHBA (200 mg/L).

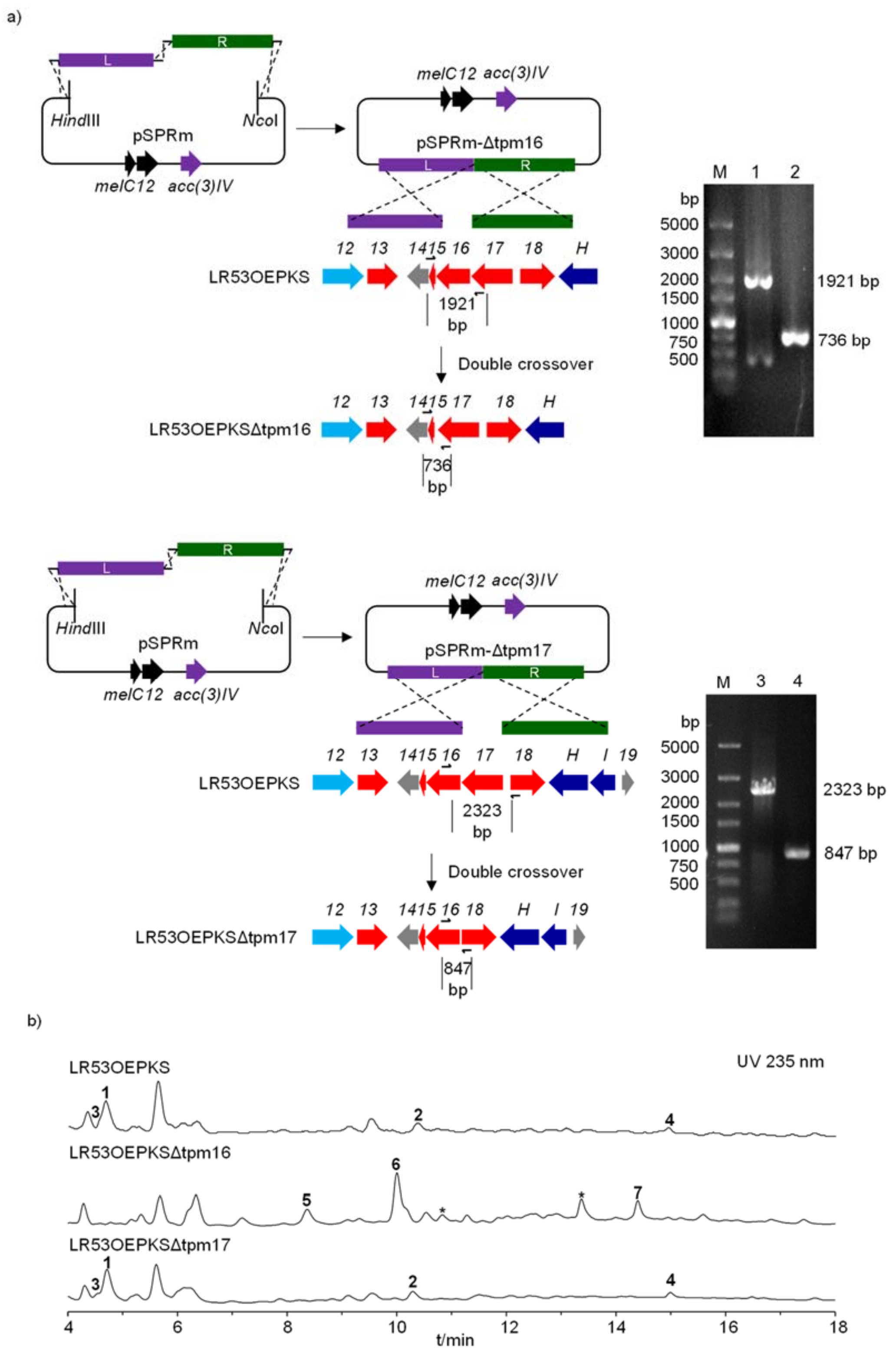

Figure S6. Construction of the mutants LR53OEPKSΔtpm16/17 and HPLC metabolite profiling. a) Construction of the *tpm16* gene and the *tpm17* gene deletion mutants LR53OEPKSΔtpm16/17 through homologous recombination and PCR verification of the mutants. M, DNA marker (DL5000); 1, LR53OEPKS (1921 bp expected); 2, the mutant LR53OEPKSΔtpm16 (736 bp expected); 3, LR53OEPKS (2323 bp expected); 4, the mutant LR53OEPKSΔtpm17 (847 bp expected). b) HPLC metabolite profiling of the mutants with AHBA (200 mg/L).

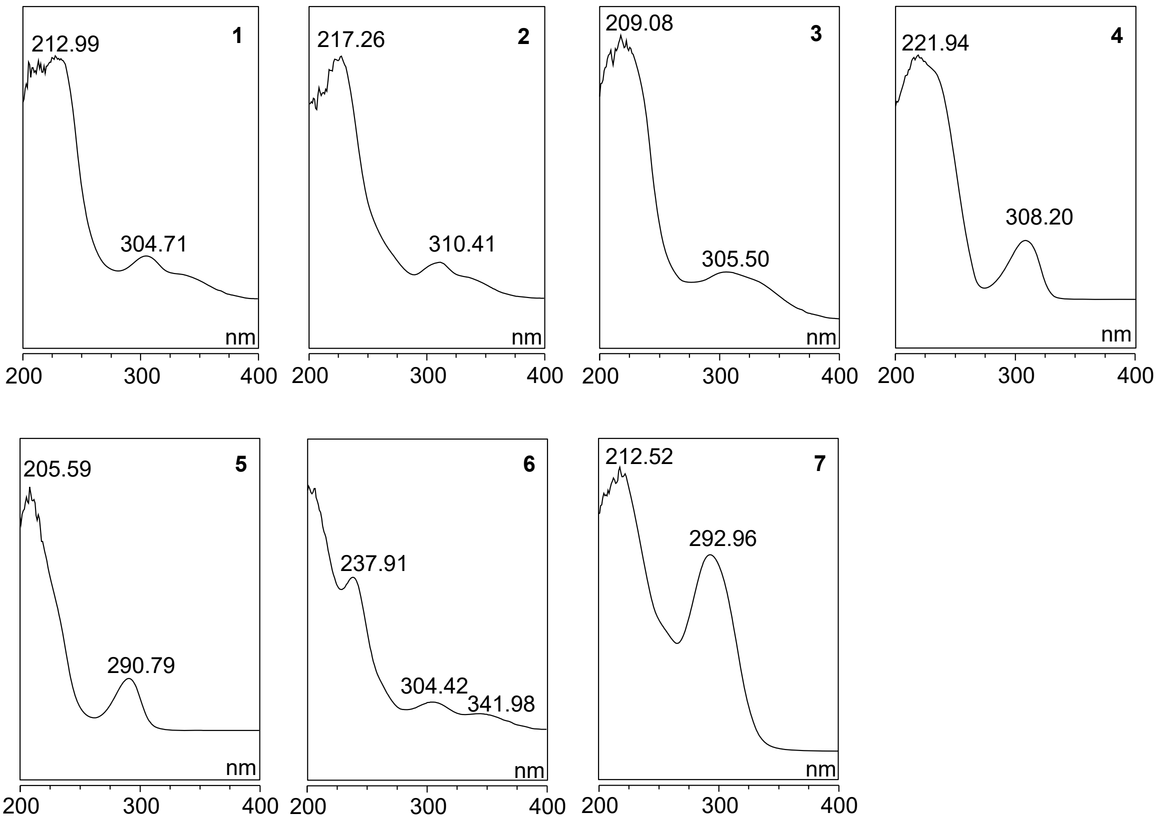

Figure S7. The UV spectra of **1**−**7**.

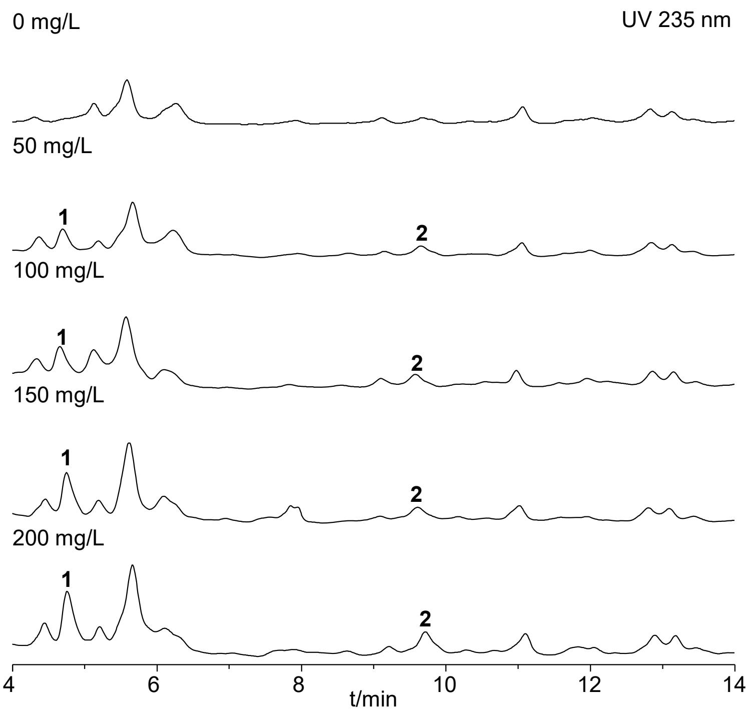

Figure S8. HPLC analysis of metabolites produced by LR53 WT cultured in the presence of varying concentrations of AHBA.

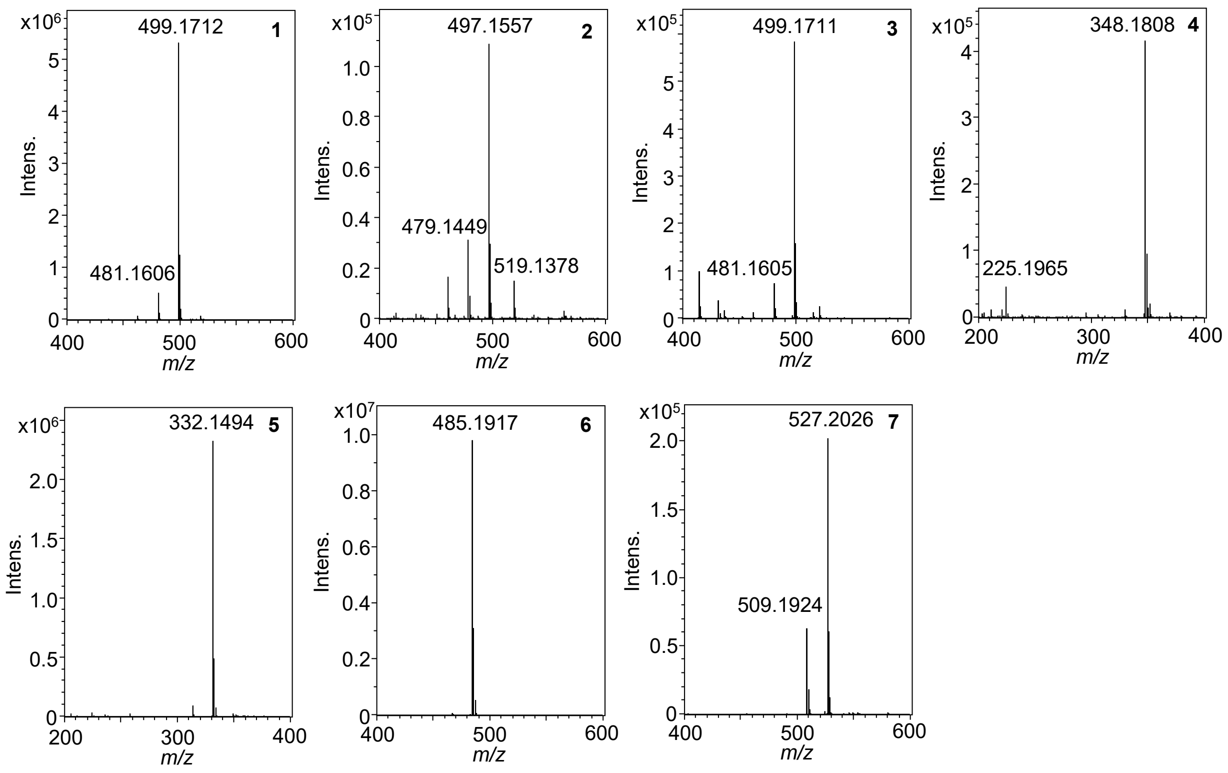

Figure S9. The HR-MS spectra of **1**−**7**.

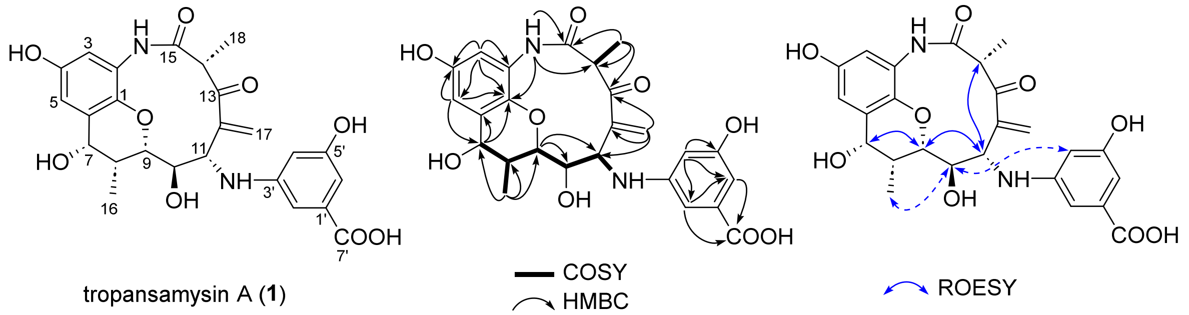

Figure S10. The key HMBC, COSY, and ROESY correlations of **1**.

Table S4. NMR spectroscopic data for **1** in DMSO-d_6 (δ in ppm, J in Hz).

No.	^{1}H	^{13}C	COSY	HMBC	ROESY
1		140.3			
2		124.5			
2-NH	9.12 (s, 1H)			C-1, C-14, C-15	H-3
3	6.30 (d, 1H, J = 3.1 Hz)	113.1		C-1, C-2, C-4, C-5	H-11, H-14, H-17
4		151.4			
5	6.77 (d, 1H, J = 3.2 Hz)	112.8		C-1, C-3, C-4, C-7	H-7
6		128.7			
7	4.75 (d, 1H, J = 6.0 Hz)	67.5	H-8	C-1, C-6, C-8, C-9, C-16	H-5, H-8, H-9
8	2.45-2.42 (m, 1H)	31.9	H-7, H-16	C-6, C-7, C-9, C-16	H-7, H-9, H-16
9	3.78 (d, 1H, J = 9.2 Hz)	78.6	H-10	C-7, C-10, C-11, C-16	H-7, H-8, H-11
10	3.35 (t, 1H, J = 9.0 Hz)	71.9	H-9, H-11	C-9, C-11	H-16, H-4’
11	4.42-4.40 (m, 1H)	59.1	H-10	C-10, C-12	H-9, H-14, H-2’, H-4’
12		144.3			
13		200.1			
14	3.18-3.17 (m, 1H)	51.4	H-18	C-13, C-15, C-18	H-11, H-18
15		171.9			
16	0.69 (d, 3H, J = 6.9 Hz)	5.4	H-8	C-7, C-8, C-9	H-8, H-10
17	5.72 (s, 1H)	123.0			
	6.43 (s, 1H)				
18	1.36 (d, 3H, J = 7.5 Hz)	18.5	H-14	C-13, C-14, C-15	H-14, H-17
1’		132.6			
2’	6.65 (s, 1H)	106.2		C-4’, C-6’, C-7’	H-11, H-4’
3’		149.3			
3’-NH	6.01 (s, 1H)		H-11		H-17
4’	6.04 (s, 1H)	103.4		C-2’, C-5’, C-6’	H-10, H-11, H-2’
5’		158.4			
6’	6.54 (s, 1H)	104.7		C-2’, C-4’, C-5’, C-7’	
7’		168.3			

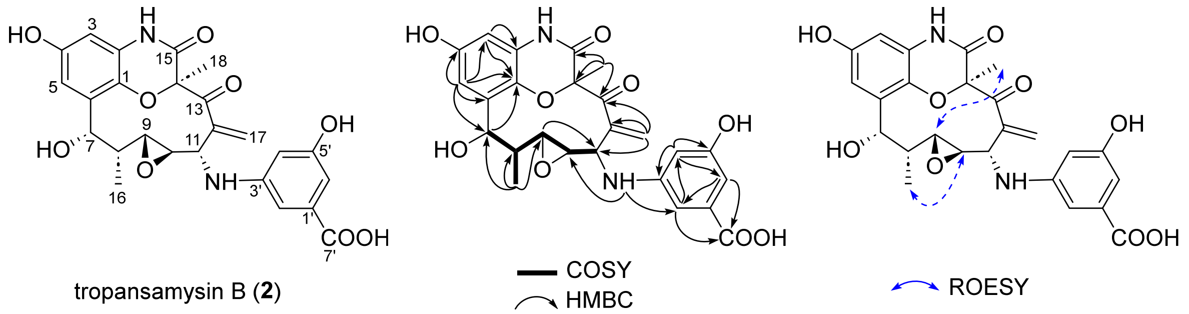

Figure S11. The key HMBC, COSY, and ROESY correlations of **2**.

Table S5. NMR spectroscopic data for **2** in DMSO-d_6 (δ in ppm, J in Hz).

No.	^{1}H	^{13}C	COSY	HMBC	ROESY
1		129.6			
2		124.7			
3	6.24 (d, 1H, J = 3.1 Hz)	101.3		C-1, C-2, C-4, C-5	
4		151.4			
5	6.47 (d, 1H, J = 2.8 Hz)	109.9		C-1, C-3, C-4, C-6, C-7	H-16
6		129.9			
7	5.13 (d, 1H, J = 3.3 Hz)	65.5	H-8	C-1, C-5, C-6, C-8, C-9, C-16	H-8, H-9
8	2.58-2.56 (m, 1H)	40.4	H-16	C-6, C-7, C-9, C-10, C-16	H-7, H-9, H-16
9	3.05 (t, 1H, J = 2.6 Hz)	57.4	H-8, H-10	C-8, C-16	H-7, H-8, H-10, H-11, H-18
10	2.47-2.50 (m, 1H)	55.4	H-11	C-9, C-11	H-10, H-16, H-17, 3’-NH
11	3.57-3.54 (m, 1H)	58.9	H-10	C-10, C-12, C-13, C-17	H-9, H-10, H-2’, H-4’
12		146.8			
13		195.5			
14		82.6			
15		165.0			
16	0.51 (d, 3H, J = 7.0 Hz)	5.7	H-8	C-7, C-8, C-9	H-5, H-8, H-10
17	5.46 (d, 2H, J = 2.1 Hz)	118.1		C-11, C-12, C-13	H-10, 3’-NH
18	1.61 (s, 3H)	21.2		C-13, C-14, C-15	H-9
1’					
2’	6.72 (brs, 1H)	105.7		C-4’, C-6’, C-7’	H-11
3’		147.5			
3’-NH	6.54 (d, 7.6, 1H)		H-11	C-10, C-2’, C-4’	H-10, H-17
4’	6.19 (brs, 1H)	102.9		C-2’, C-3’, C-5’, C-6’	H-11
5’		157.8			
6’	6.59 (brs, 1H)	105.2		C-2’, C-4’, C-5’, C-7’	
7’		168.5			

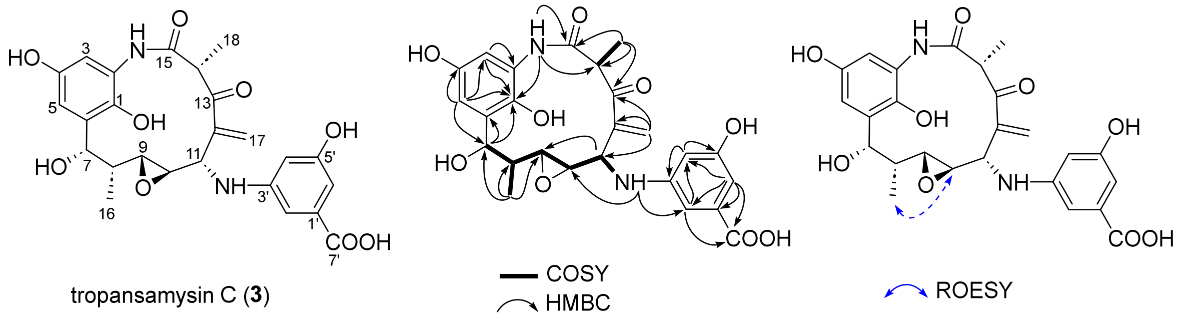

Figure S12. The key HMBC, COSY, and ROESY correlations of **3**.

Table S6. NMR spectroscopic data for **3** in DMSO-d_6 (δ in ppm, J in Hz).

No.	^{1}H	^{13}C	COSY	HMBC	ROESY
1		142.7			
2		123.2			
2-NH	8.34 (s, 1H)			C-1, C-2, C-3, C-14, C-15	H-3
3	6.48 (d, 1H, J = 3.0 Hz)	115.7		C-1, C-3, C-4, C-7	H-14, 2-NH
4		150.0			
5	6.69 (d, 1H, J = 3.0 Hz)	115.6		C-1, C-2, C-4, C-5	H-16
6		131.1			
7	5.33 (d, 1H, J = 6.2 Hz)	63.7	H-8	C-1, C-5, C-6, C-8, C-9, C-16	H-8, H-9
8	2.57-2.54 (m, 1H)	39.2	H-7, H-16	C-6, C-7, C-9, C-10, C-16	H-7, H-9, H-16
9	2.92-2.91 (m, 1H)	56.0	H-10	C-7, C-8, C-10, C-11, C-16	H-7, H-8, H-10, H-11
10	2.60 (dd, 1H, J = 8.3, 2.3 Hz)	57.0	H-11	C-9, C-11	H-9, H-16, H-17, 3'-NH
11	3.74 (t, 1H, J = 8.0 Hz)	50.9	H-10, 3'-NH	C-9, C-10, C-12, C-13, C-17, C-3'	H-9, H-10, H-2', H-4'
12		144.3			
13		196.2			
14	4.45 (q, 1H, J = 6.8 Hz)	43.7	H-18	C-13, C-15, C-18	H-3, H-17, H-18
15		172.1			
16	0.72 (d, 3H, J = 6.9 Hz)	5.7	H-8	C-7, C-8, C-9	H-5, H-8, H-10
17	5.93 (s, 1H)<br>5.89 (s, 1H)	127.5			H-10, H-11, H-14
18	0.99 (d, 3H, J = 7.0 Hz)	15.2		C-13, C-14, C-15	H-14, H-17
1'		132.3			
2'	6.31 (brs, 1H)	105.0		C-1', C-4', C-6', C-7'	H-11
3'		148.3			
3'-NH	6.36 (d, 1H, J = 8.0 Hz)			C-10, C-11, C-2', C-4'	H-17, H-4'
4'	5.91-5.90 (m, 1H)	103.7		C-2', C-3', C-5'	H-11
5'		157.9			
6'	6.51 (dd, 1H, J = 2.2, 1.4 Hz)	104.8		C1', C-2', C-4', C-5', C-7'	
7'		167.7			

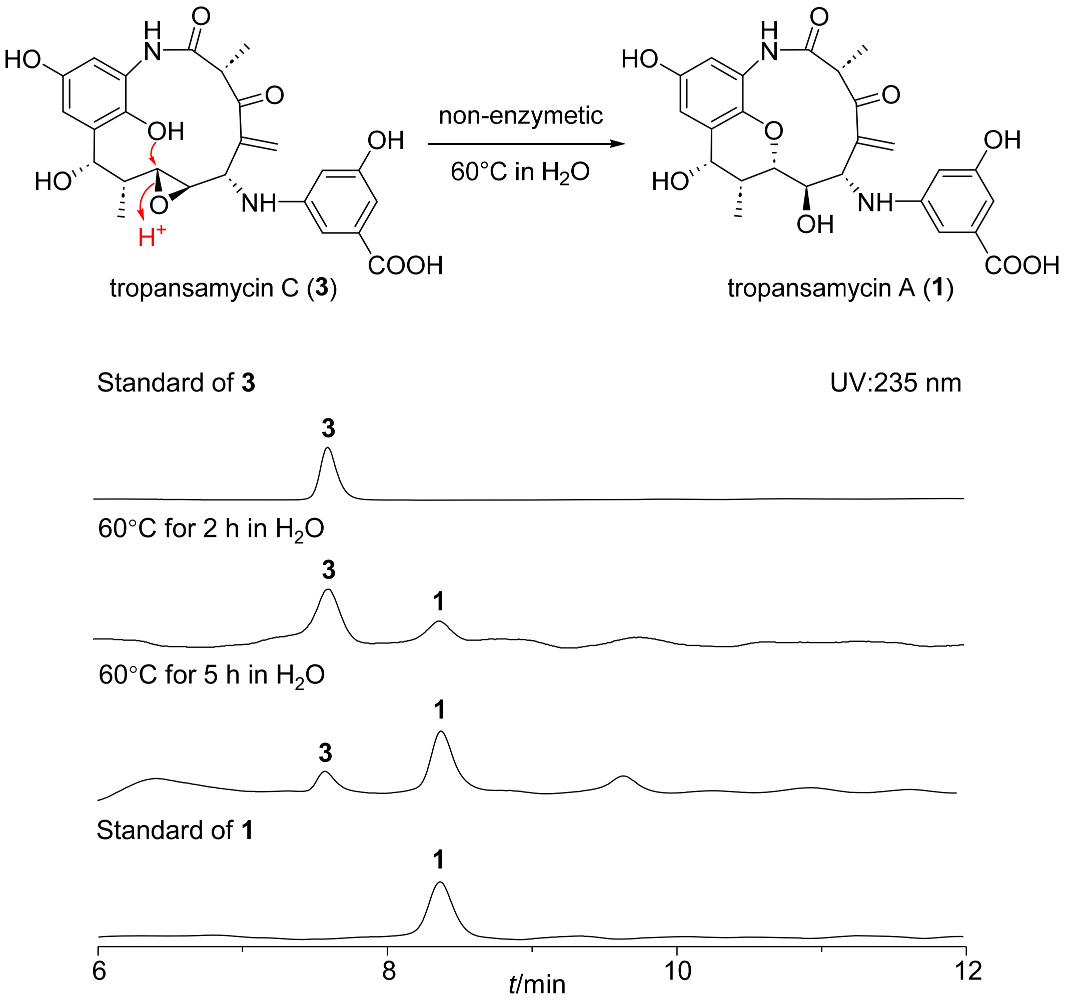

Figure S13. Nonenzymatic transformation of compound **3** to compound **1**.

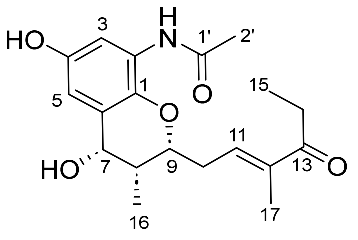

tropansamysin D (**4**)

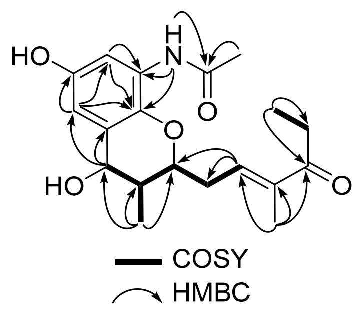

Figure S14. The key HMBC and COSY correlations of **4**.

Table S7. NMR spectroscopic data for **4** in DMSO-d_6 (δ in ppm, J in Hz).

No.	^{1}H	^{13}C	COSY	HMBC
1		137.1		
2		126.8		
2-NH	8.63 (s, 1H)			C-1, C-2, C-3, C-1’
3	7.36 (d, 1H, J = 3.0 Hz)	109.3		C-1, C-2, C-4, C-5
4		150.2		
5	6.40 (d, 1H, J = 3.0 Hz)	111.1		C-1, C-3, C-4, C-7
6		126.2		
7	4.28 (m, 1H)	66.1	H-8	C-1, C-5, C-6, C-9, C-16
7-OH	5.18 (brs, 1H)			
8	1.84-1.82 (m, 1H)	37.3	H-9, H-18	C-9, C-16
9	4.11-4.08 (m, 1H)	75.1	H-8, H-10	C-7, C-11
10	2.74-2.70 (m, 1H), 2.58-2.53 (m, 1H)	32.3	H-9, H-11	C-8, C-9, C-11, C-12
11	6.99 (t, 1H, J = 6.8 Hz)	138.8	H-10	C-9, C-10, C-13, C-17
12		137.7		
13		202.1		
14	2.76-2.71 (m, 2H)	30.3	H-15	C-12, C-13, C-15
15	0.98 (t, 3H, J = 7.3 Hz)	9.2	H-14	C-13, C-14
16	0.99 (d, 3H, J = 7.0 Hz)	13.0	H-8	C-7, C-8, C-9
17	0.95 (s, 3H)	11.9		C-11, C-12, C-13
1’		168.4		
2’	2.01 (s, 3H)	24.3		C-1’

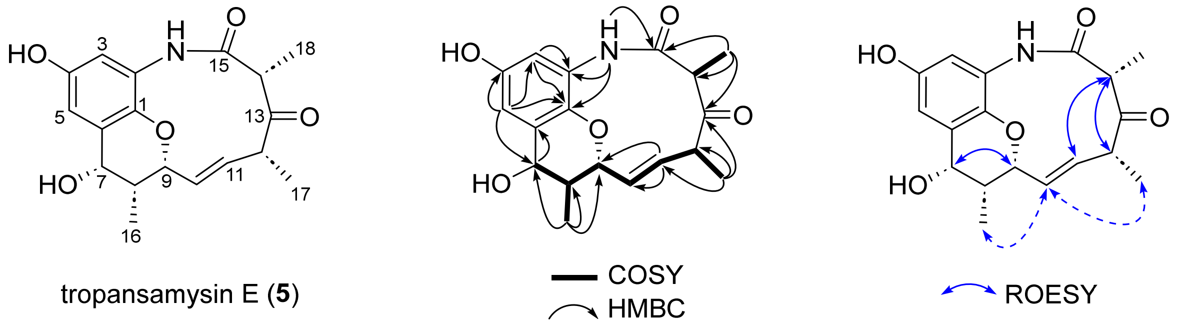

Figure S15. The key HMBC, COSY, and ROESY correlations of **5**.

Table S8. NMR spectroscopic data for **5** in DMSO-d_6 (δ in ppm, J in Hz).

No.	^{1}H	^{13}C	COSY	HMBC	ROESY
1		141.6			
2		127.9			
2-NH	9.75 (s, 1H)			C-1, C-2, C-3, C-15	H-11, H-14
3	6.33 (d, 1H, J = 2.9 Hz)	113.7		C-1, C-2, C-4, C-5	
4		151.9			
5	6.63 (dd, 1H, J = 2.9, 1.2 Hz)	110.0		C-1, C-3, C-4, C-7	
6		135.6			
7	4.80 (brs, 1H)	67.7	H-8	C-6, C-16	H-8, H-9, H-10
8	2.61-2.54 (m, 1H)	39.2	H-9, H-16	C-6, C-7, C-16	H-7, H-9, H-16
9	5.03-5.01 (m, 1H)	76.6	H-8	C-1, C-8, C-10, C-11, C-16	H-7, H-8, H-10
10	5.59 (dt, 1H, J = 15.6, 2.3 Hz)	127.9	H-11	C-8, C-9, C-11, C-12	H-9, H-16, H-17
11	7.36 (ddd, 1H, J = 15.6, 4.4, 2.3 Hz)	131.1	H-10, H-12	C-9, C-10, C-12, C-13, C-17	2-NH, H-12, H-14
12	3.24-3.19 (m, 1H)	48.8	H-17		H-11, H-14, H-17
13		204.5			
14	4.18 (q, 1H, J = 6.6 Hz)	52.6	H-18	C-13, C-15, C-18	2-NH, H-11, H-12, H-18
15		171.7			
16	0.21 (d, 3H, J = 7.1 Hz)	8.7	H-8	C-7, C-8, C-9	H-8, H-10
17	1.05 (d, 3H, J = 6.7 Hz)	11.9	H-12	C-11, C-12, C-13	H-10, H-12
18	1.17 (d, 3H, J = 6.7 Hz)	14.6		C-13, C-14, C-15	H-14

Table S9. X-ray crystallographic data and structure refinement for tropansamycin E (**5**).

Identification code (deposition number)	**5** (CCDC 2414079)
Empirical formula	$C_{18}H_{21}NO_5$
Formula weight	331.14
Temperature/K	100.00
Crystal system	monoclinic
Space group	$P2_1$
a/Å	8.6907(3)
b/Å	9.4880(3)
c/Å	11.7681(3)
α/°	90
β/°	111.6
γ/°	90
Volume/Å^3	902.02
Z	2
ρ_{calc}g/cm^3	1.338
μ/mm^{-1}	0.825
F (000)	388.0
Crystal size/mm^3	0.1 × 0.1 × 0.15
Radiation	Cu Kα (λ = 1.54184)
2Θ range for data collection/°	8.082 to 143.152
Index ranges	$-10 \le h \le 10$, $-10 \le k \le 11$, $-13 \le l \le 20$
Reflections collected	7432
Independent reflections	3045 [R_{int} = 0.0372, R_{sigma} = 0.0274]
Data/restraints/parameters	3045/1/245
Goodness-of-fit on F^2	1.047
Final R indexes [I>=2σ (I)]	R_1 = 0.0389, wR_2 = 0.1057
Final R indexes [all data]	R_1 = 0.0390, wR_2 = 0.1058
Largest diff. peak/hole/eÅ^{-3}	0.27/-0.27
Flack parameter	-0.03 (13)
Melting point/°C	209.4

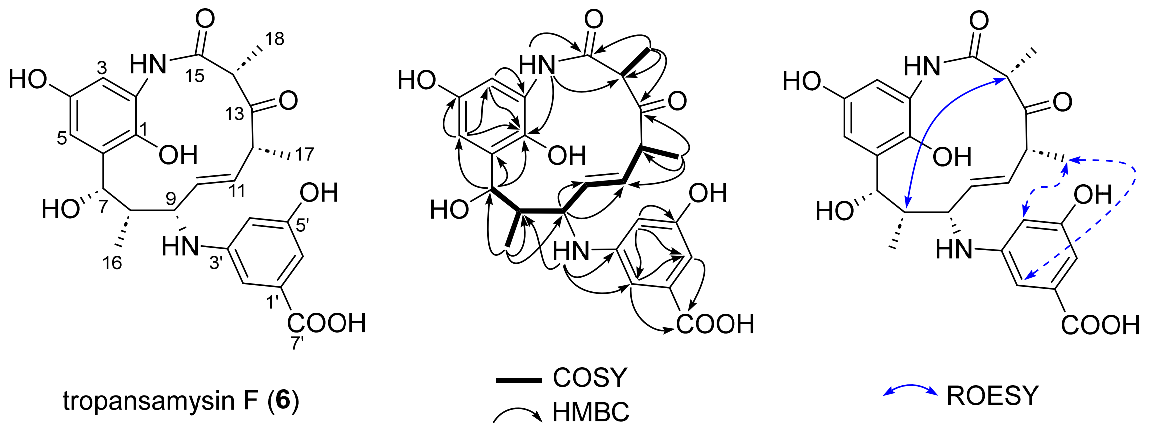

Figure S16. The key HMBC, COSY, and ROESY correlations of **6**.

Table S10. NMR spectroscopic data for **6** in DMSO-d_6 (δ in ppm, J in Hz).

No.	^{1}H	^{13}C	COSY	HMBC	ROESY
1		141.6			
2		124.3			
2-NH	8.73 (s, 1H)			C-1, C-2, C-3, C-14, C-15	H-3, H-14
3	6.03 (d, 1H, J = 3.1 Hz)	114.5		C-1, C-2, C-4, C-5	2-NH
4		149.5			
5	6.90 (d, 1H, J = 3.1 Hz)	112.3		C-1, C-3, C-4, C-7	
6		134.0			
7	4.90 (brs, 1H)	71.6	H-8	C-1, C-5, C-6, C-8, C-9, C-16	H-8, H-16
8	2.70-2.66 (m, 1H)	40.0	H-16	C-7, C-9, C-16	H-7, H-9, H-16
9	3.67 (t, 1H, J = 6.6 Hz)	60.9	H-10, 3'-NH	C-7, C-8, C-10, C-11, C-16, C-3'	H-8, H-10, H-16, H-2', H-4'
10	4.61 (dd, 1H, J = 16.6, 6.6 Hz)	138.2	H-9, H-11	C-9, C-11, C-12	H-8, H-9, H-12, H-14, H-17
11	5.50 (dd, 1H, J = 16.1, 8.0 Hz)	127.0	H-10, H-12	C-9, C-12, C-17	H-9, H-12, H-14, H-17
12	3.10-3.04 (m, 1H)	43.7	H-11, H-17	C-10, C-11, C-13, C-17	H-11, H-14, H-17, H-18
13		208.9			
14	3.95 (q, 1H, J = 7.2 Hz)	47.4	H-18	C-13, C-15, C-18	H-11, H-8, H-12, H-18
15		172.1			
16	1.27 (d, 3H, J = 7.1 Hz)	19.0	H-8	C-7, C-8, C-9	H-7, H-8, H-9
17	0.68 (d, 3H, J = 6.8 Hz)	14.2	H-12	C-11, C-12, C-13	H-10, H-12, H-18, H-2', H-4'
18	1.16 (d, 3H, J = 7.3 Hz)	13.5	H-14	C-13, C-14, C-15	H-12, H-14, H-17
1'		132.2			
2'	6.45 (brs, 1H)	104.9		C-4', C-6', C-7'	H-9, 3'-NH
3'		149.6			
3'-NH	6.15 (d, 5.8)				H-5, H-9, H-16, H-2'
4'	5.94 (brs, 1H)	102.5		C-2', C-5', C-6'	H-9
5'		158.2			
6'	6.51 (brs, 1H)	103.7		C-2', C-4', C-5', C-7'	
7'		168.0			

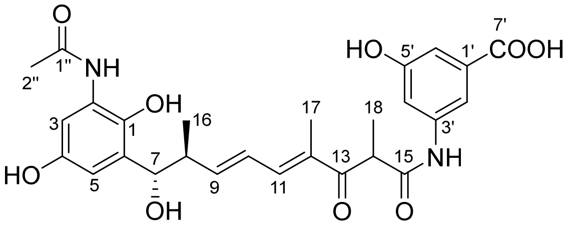

tropansamysin G (7)

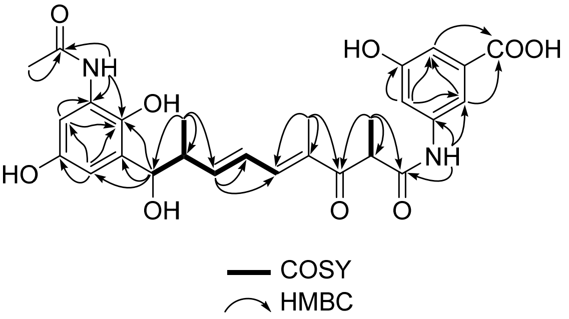

Figure S17. The key HMBC and COSY correlations of **7**.

Table S11. NMR spectroscopic data for **7** in DMSO-d_6 (δ in ppm, J in Hz).

No.	^{1}H	^{13}C	COSY	HMBC
1		137.9		
2		126.8		
2-NH	9.79 (s, 1H)			C-1, C-2, C-3, C-1”
3	6.72 (d, 1H, J = 2.9 Hz)	107.8		C-1, C-2, C-4, C-5
4		149.7		
5	6.58 (d, 1H, J = 3.6 Hz)	110.6		C-1, C-3, C-4, C-7
6		134.2		
7	4.78 (d, 1H, J = 6.1 Hz)	71.1	H-8	C-1, C-5, C-6, C-8, C-9, C-16
8	2.68-2.59 (m, 1H)	43.5	H-7, H-9, H-23	C-7, C-9, C-10, C-16
9	6.33-6.30 (m, 1H)	147.4	H-8	C-8, C-10, C-11, C-16
10	6.40 (dd, 1H, J = 15.2, 9.7 Hz)	126.2	H-11	C-8, C-11
11	7.23 (d, 1H, J = 8.0 Hz)	139.6	H-10	C-9, C-17
12		132.8		
13		197.8		
14	4.33 (qd, 1H, J = 7.0, 1.7 Hz)	47.9	H-25	C-13, C-15, C-18
15		169.5		
16	0.99 (d, 3H, J = 6.9 Hz)	16.9	H-8	C-7, C-8, C-9
17	1.78 (s, 3H)	11.8		
18	1.25 (d, 3H, J = 7.0 Hz)	14.8	H-14	C-13, C-14, C-15
1’		132.3		
2’	7.56 (t, 1H, J = 1.7 Hz)	111.2		C-3’, C-4’, C-6’, C-7’
3’		140.1		
3’-NH	10.16 (s, 1H)			C-15, C-2’, C-3’, C-4’
4’	7.36 (t, 1H, J = 2.2 Hz)	110.3		C-2’, C-3’, C-5’, C-6’
5’		157.6		
6’	7.03 (ddd, 1H, J = 4.7, 2.3, 1.4 Hz)	111.1		C-1’, C-4’, C-5’, C-7’
7’		167.4		
1”		169.9		
2”	2.10 (d, 3H, J = 1.7 Hz)	23.3		C-1”

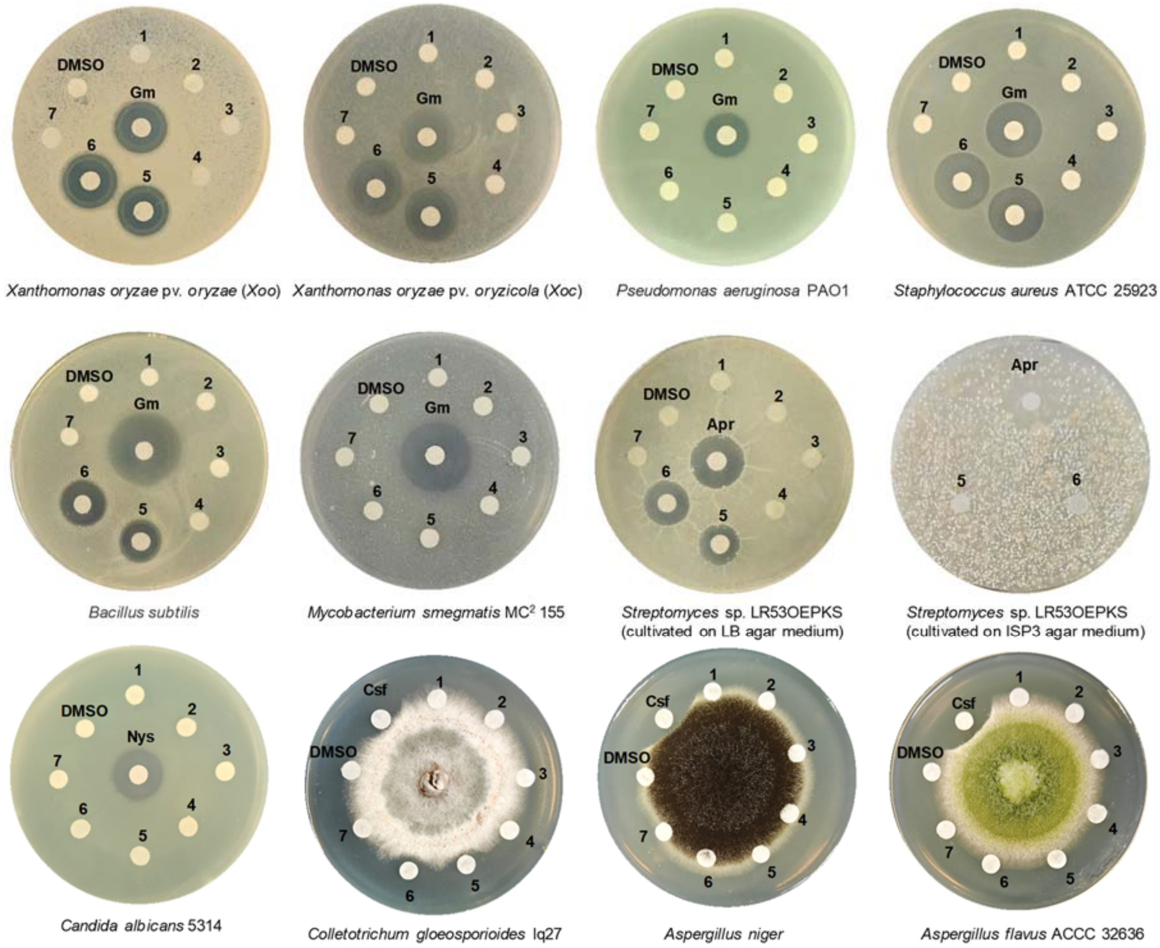

Figure S18. Antimicrobial activity of compounds **1**–**7**. Positive controls included gentamicin (Gm, 20 µg), apramycin (Apr, 5 µg), nystatin (Nys, 15 µg), and caspofungin (Csf, 10 µg).

Table S12. The inhibitory activity of compounds **1-7** against HepG2 cells.

Compounds	Concentration (μM)	Cell inhibition ± SD (%)
**1**	20	7.4 ± 3.3
**2**	20	8.0 ± 4.1
**3**	20	5.5 ± 4.6
**4**	20	2.4 ± 2.0
**5**	20	-2.8 ± 2.2
**6**	20	4.0 ± 3.8
**7**	20	6.2 ± 1.5
Cisplatin	50	72.1 ± 1.1

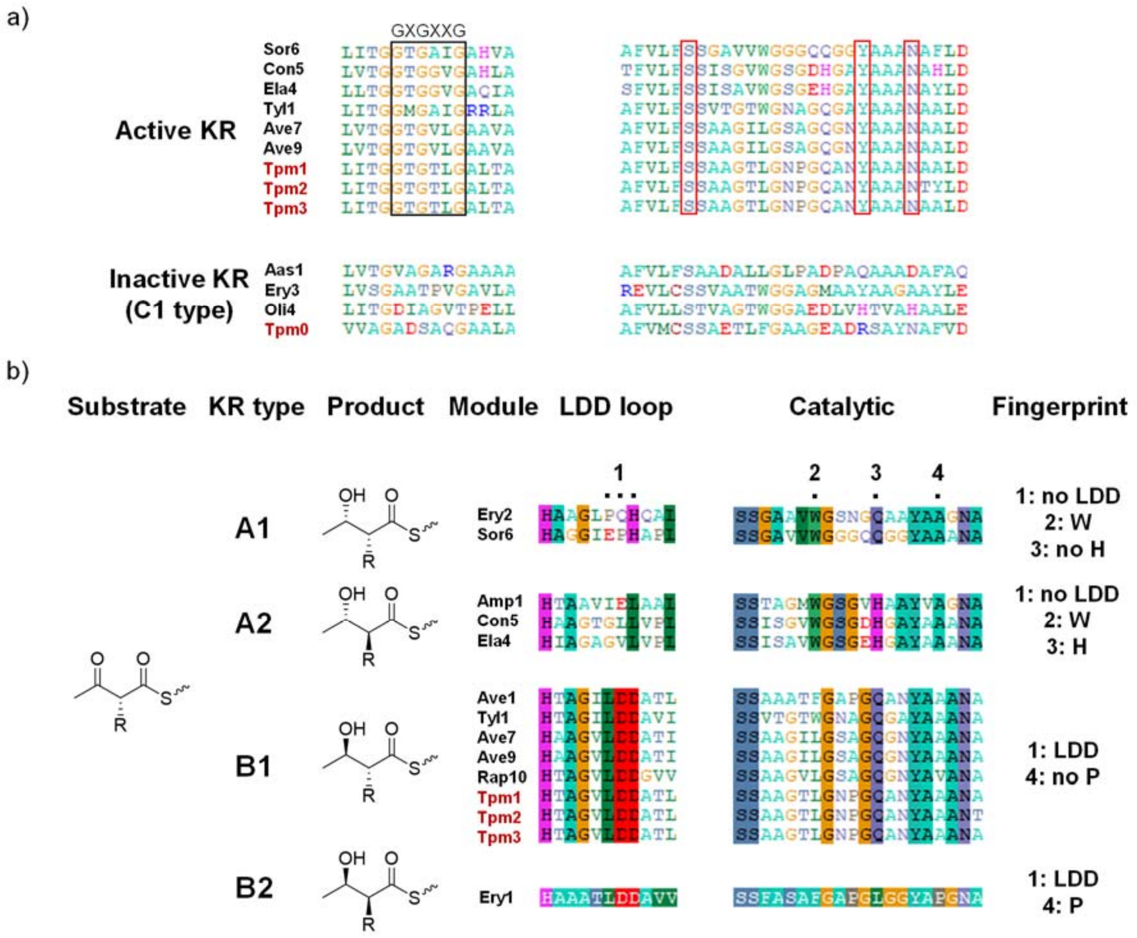

Figure S19. KR fingerprints. a) Sequence alignments of active and inactive KR with key residues highlighted. The NADPH binding motif (GXGXXG) is shown in black boxes, and catalytic triad residues are marked with red boxes. b) Sequence alignment of KR domains from tropansamycins and other antibiotics. Aas: Aminoansamycin; Ave: Avermectin; Con: Concanamycin, Ela: Elaiophylin; Ery: Erythromycin; Oli: Oligomycin; Rap: Rapamycin; Sor: Soraphen.

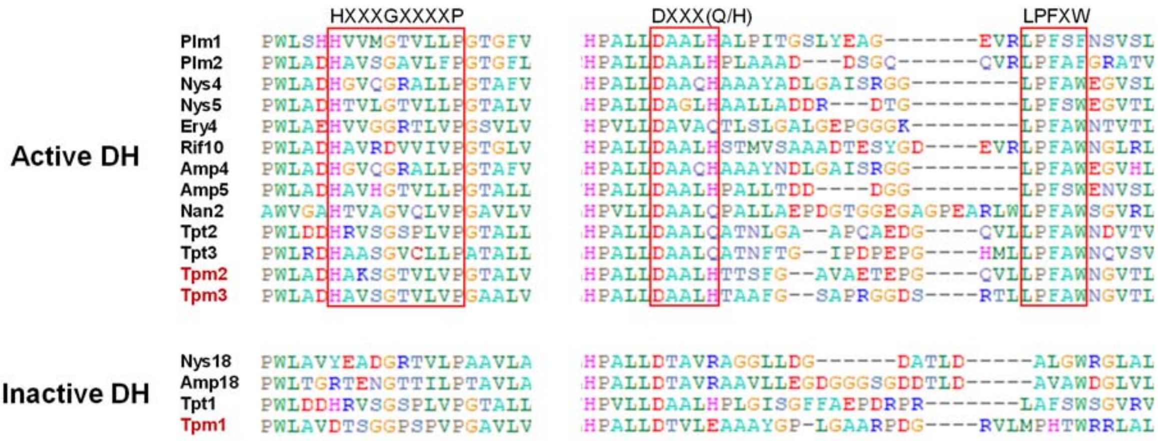

Figure S20. Sequence alignments of DH domains. The conserved HXXXGXXX, DXXX(Q/H), and LPFXW motifs are marked with red boxes. Amp: Amphotericin; Ery: Erythromycin; Nan: Nanchangmycin; Nys: Nystatin; Plm: Phoslactomycin; Rif Rifamycin; Tpt: Tetrapetalones.

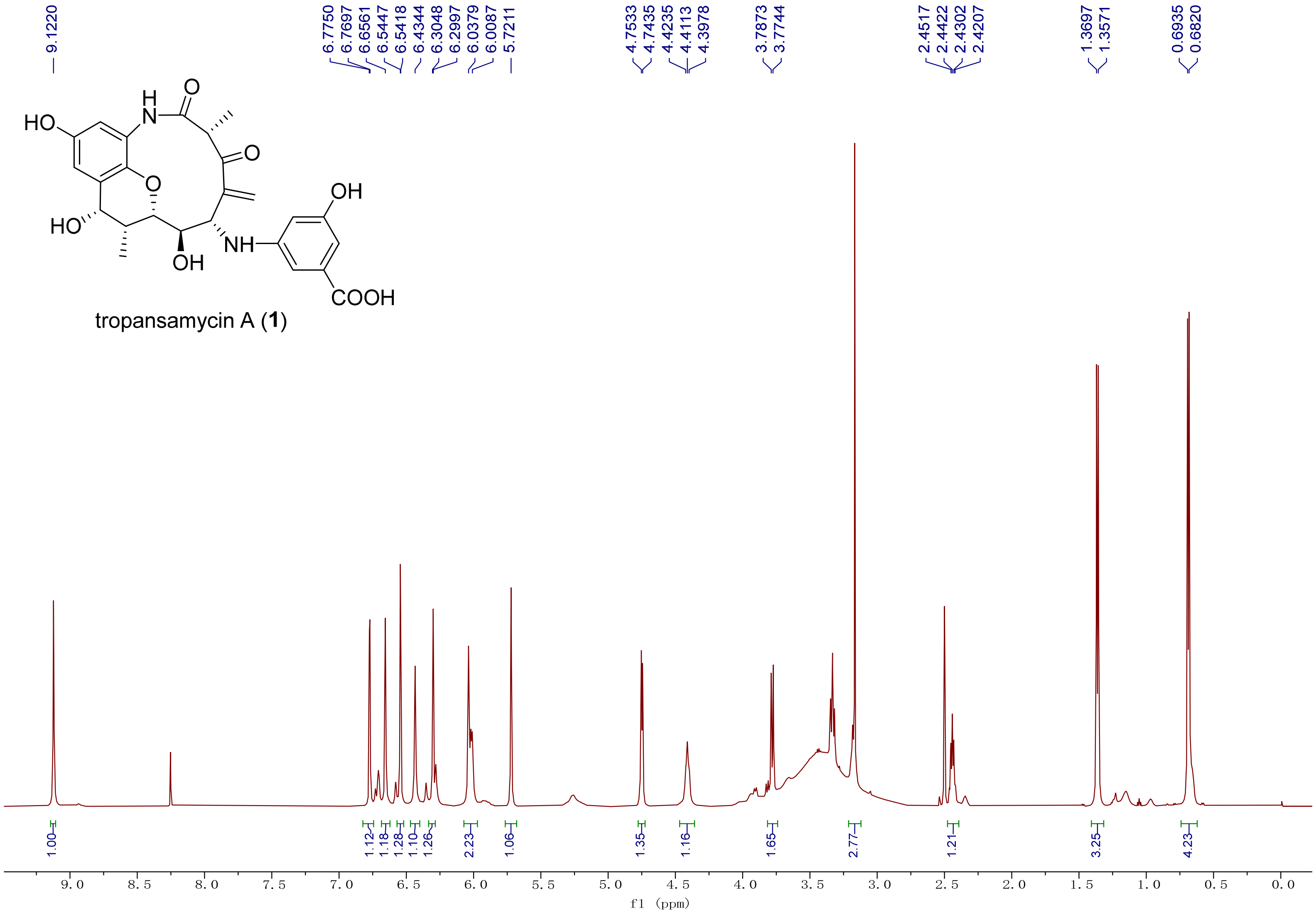

Figure S21. ^{1}H NMR spectrum of **1** in DMSO-d_6.

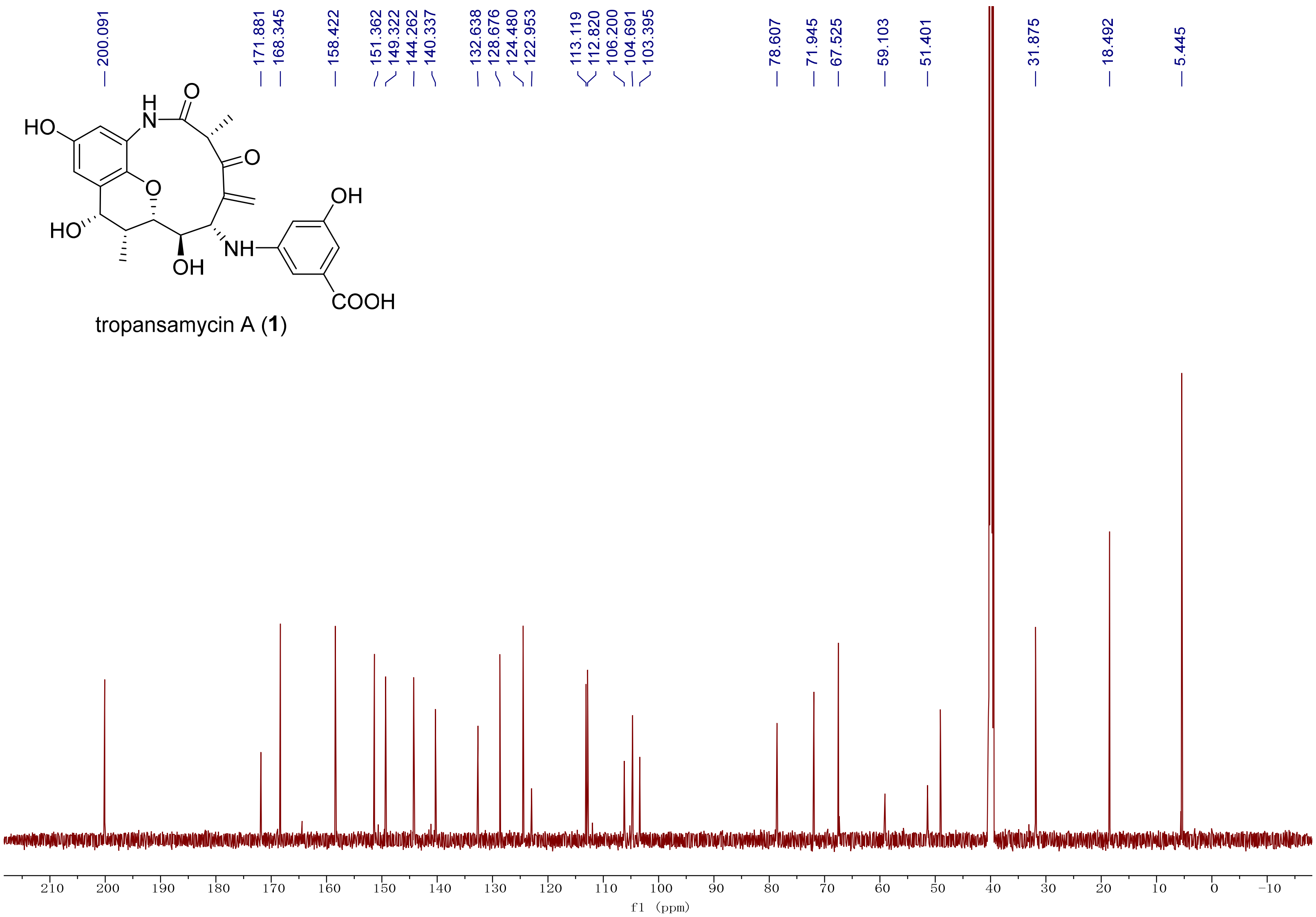

Figure S22. ^{13}C NMR spectrum of **1** in DMSO-d_6.

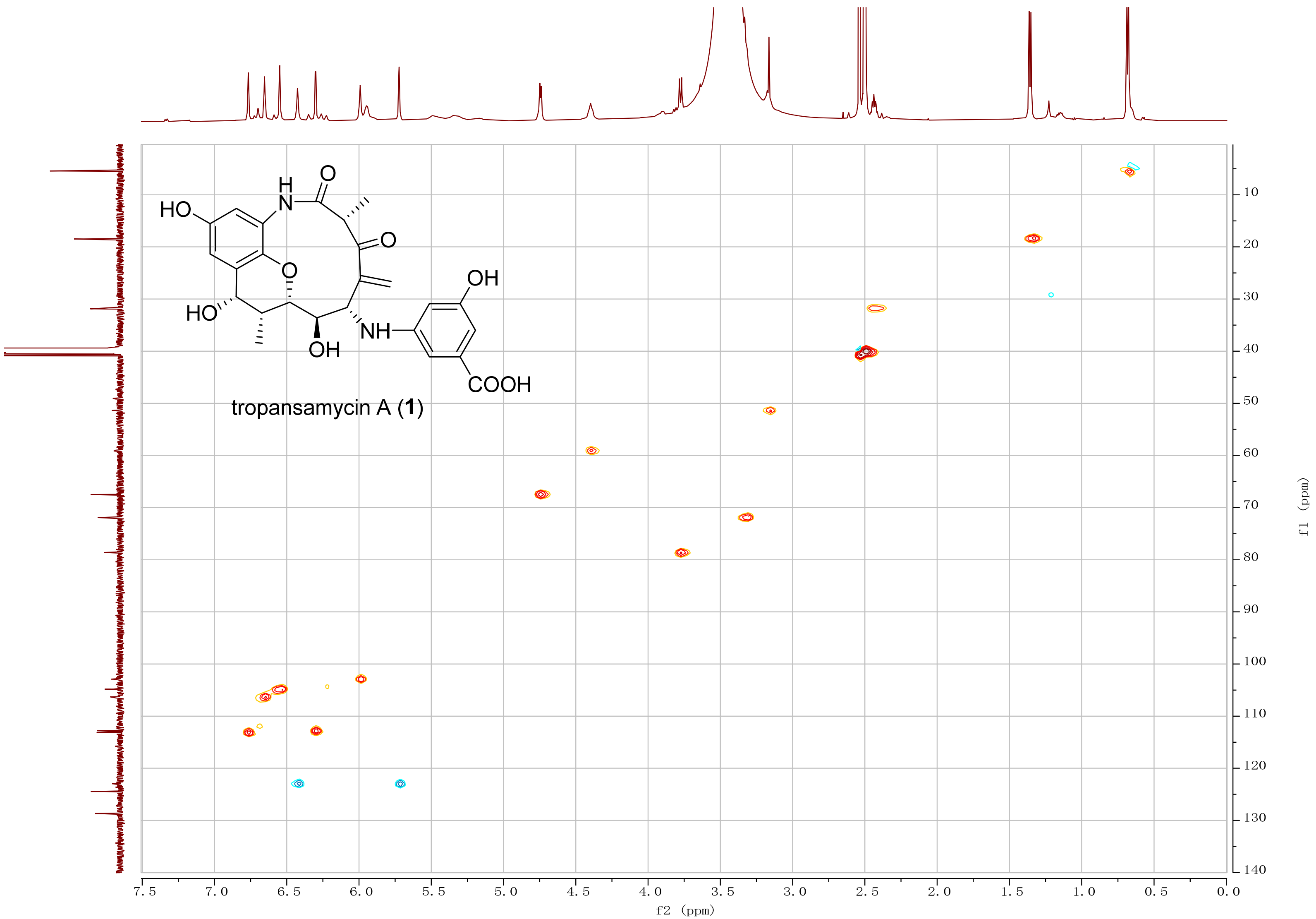

Figure S23. The HSQC spectrum of **1** in DMSO-d_6.

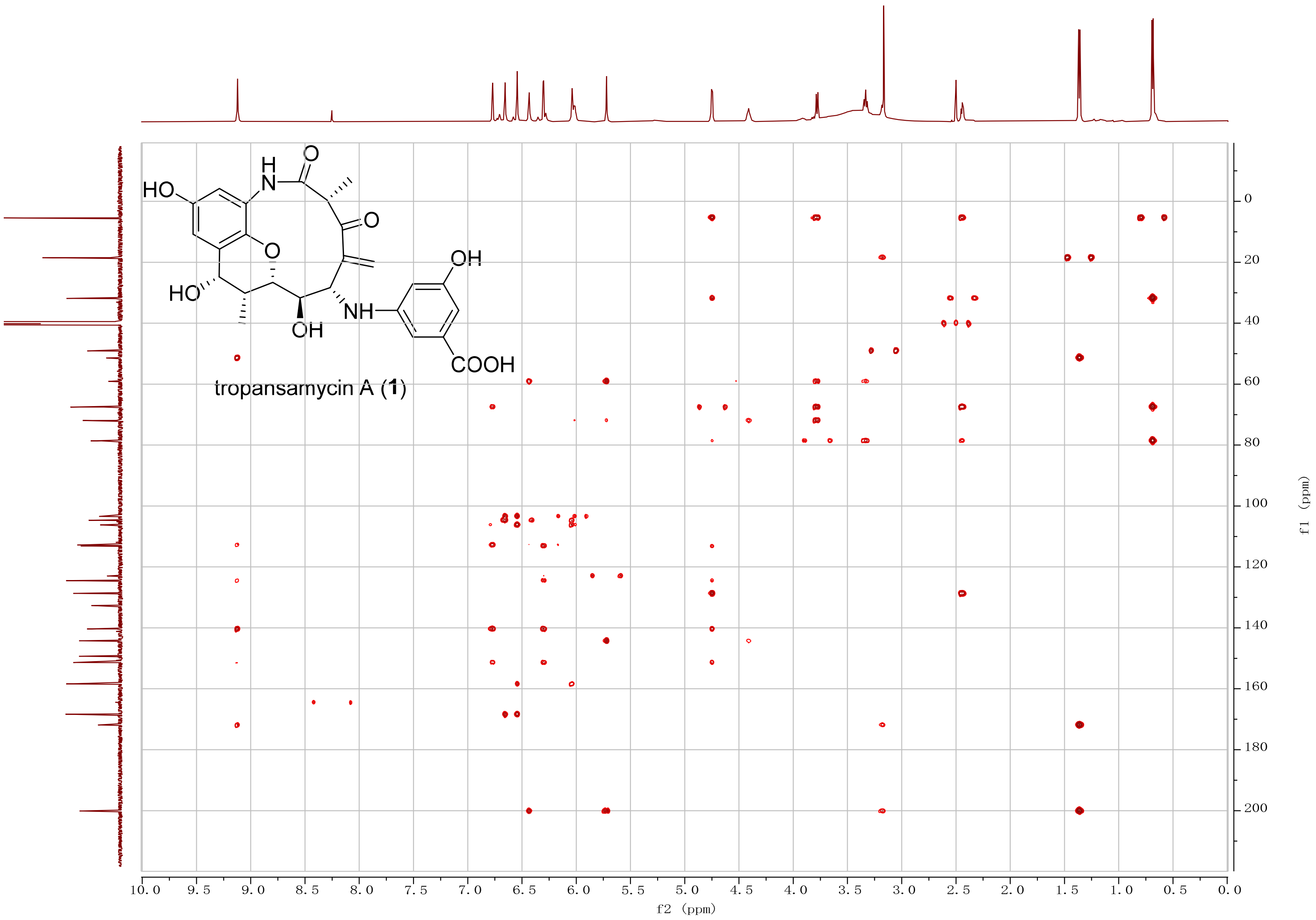

Figure S24. The HMBC spectrum of **1** in DMSO-d_6.

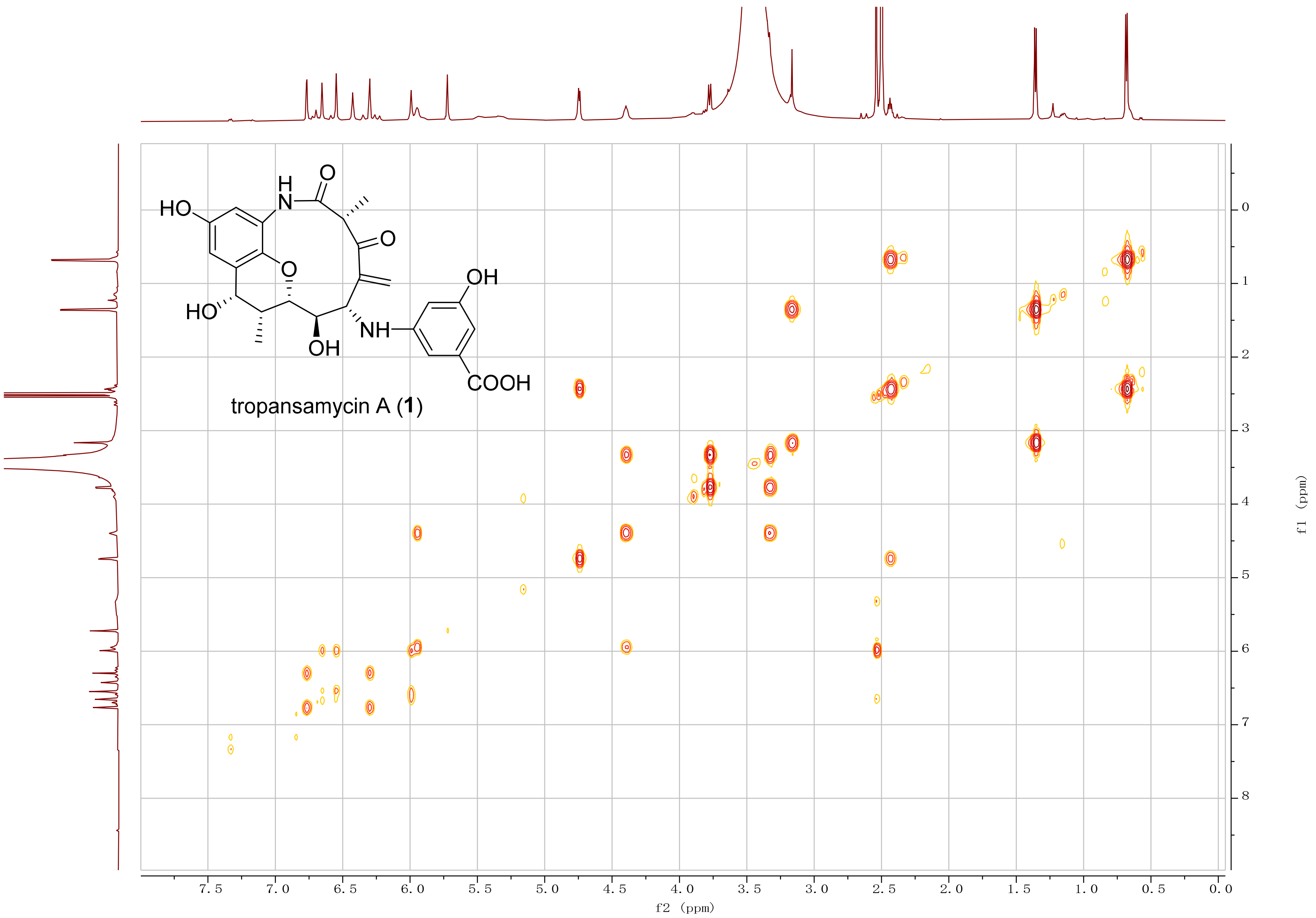

Figure S25. The ^{1}H-^{1}H COSY spectrum of **1** in DMSO-d_6.

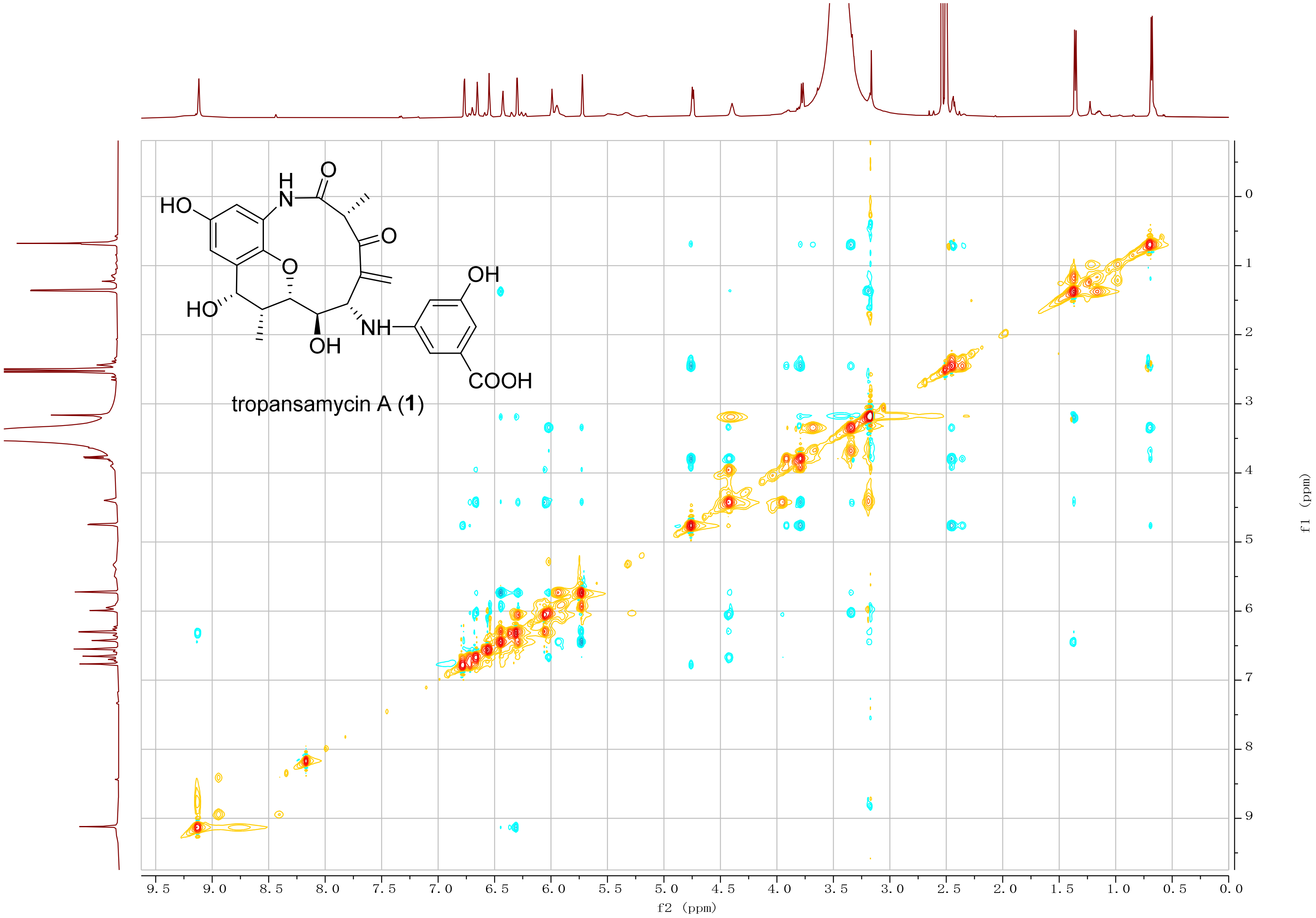

Figure S26. The ROESY spectrum of **1** in DMSO-d_6.

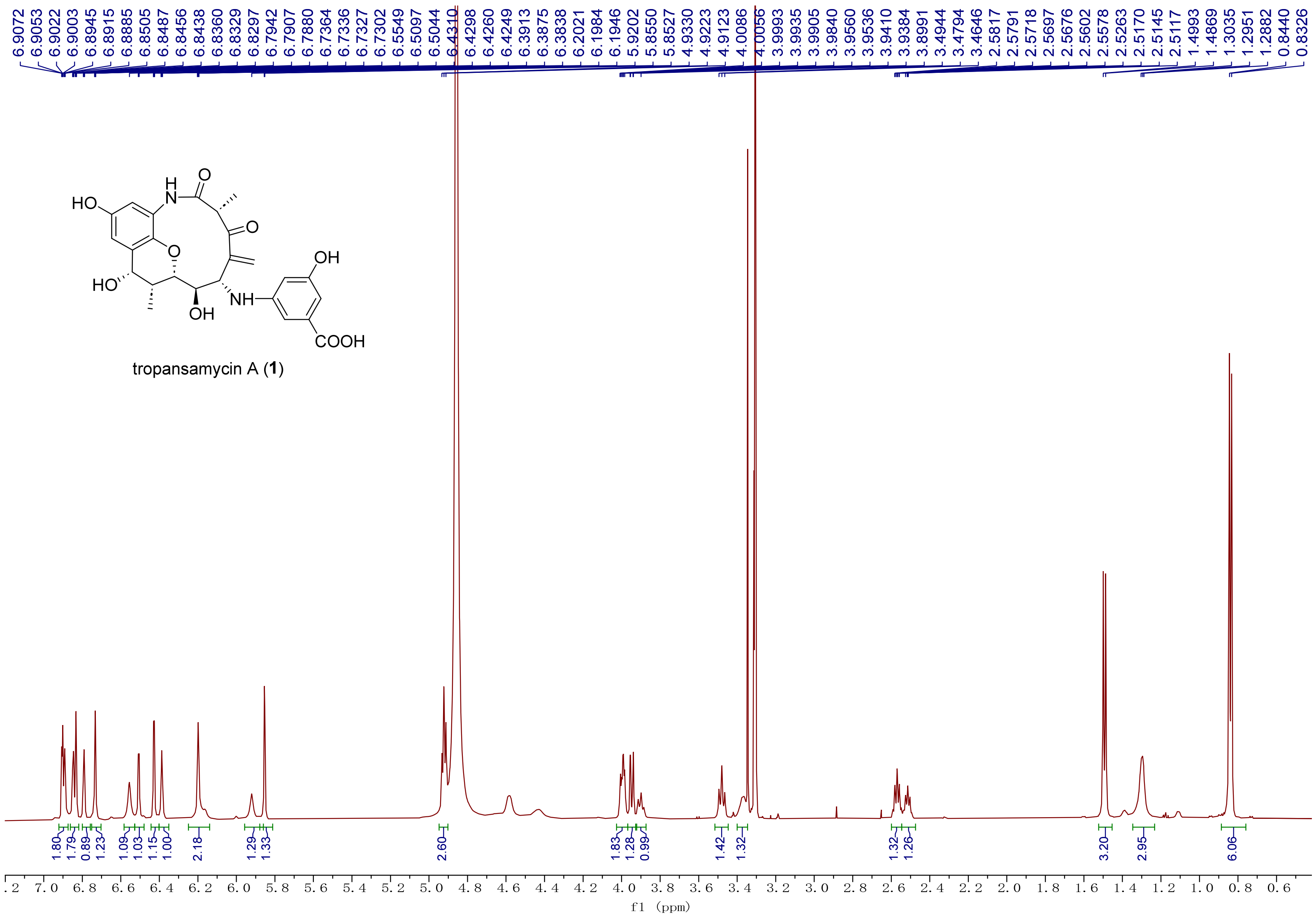

Figure S27. ^{1}H NMR spectrum of **1** in methanol-d_4.

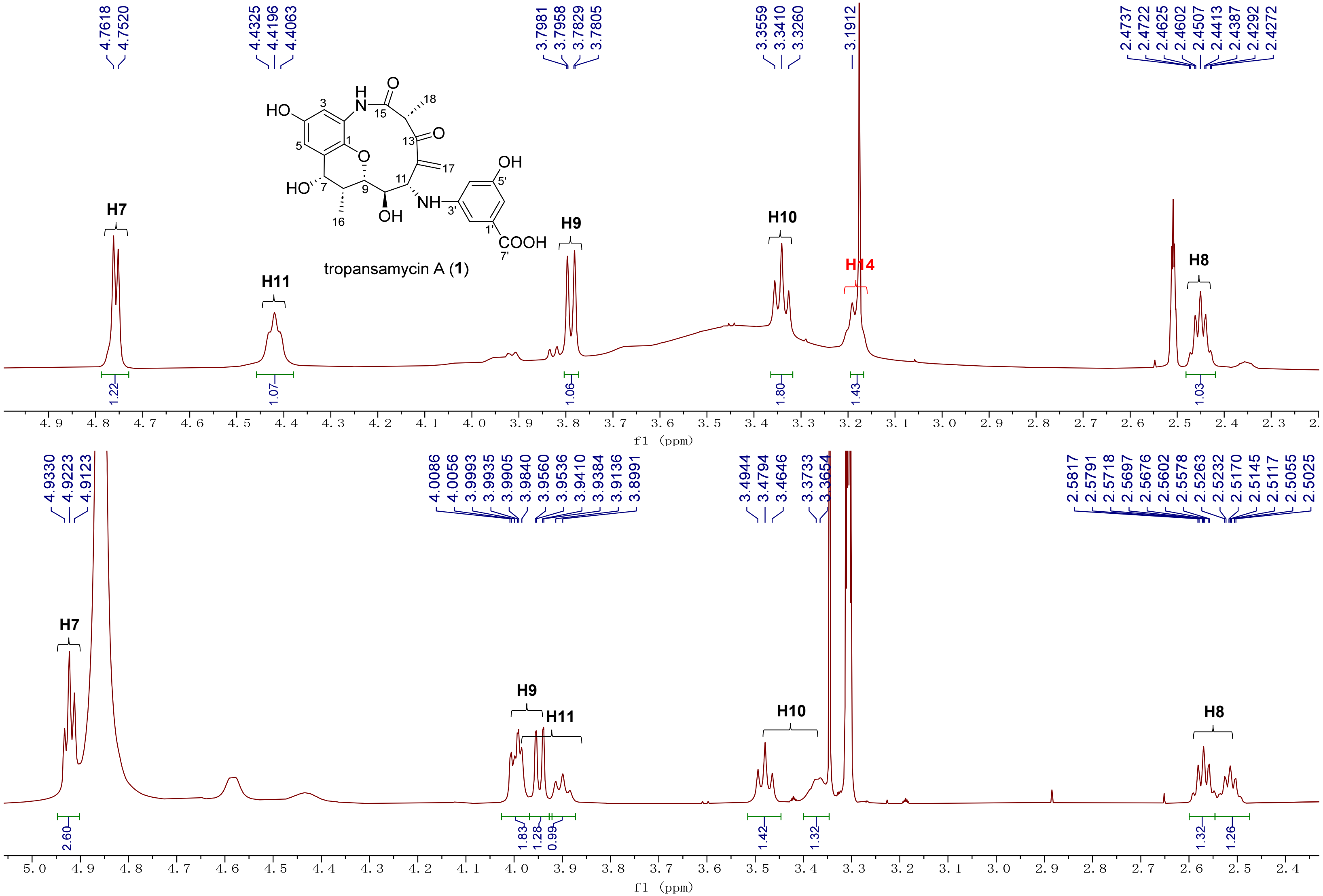

Figure S28. Comparison of partial ^{1}H NMR spectra of **1** in DMSO-d_6 (top) and methanol-d_4 (bottom).

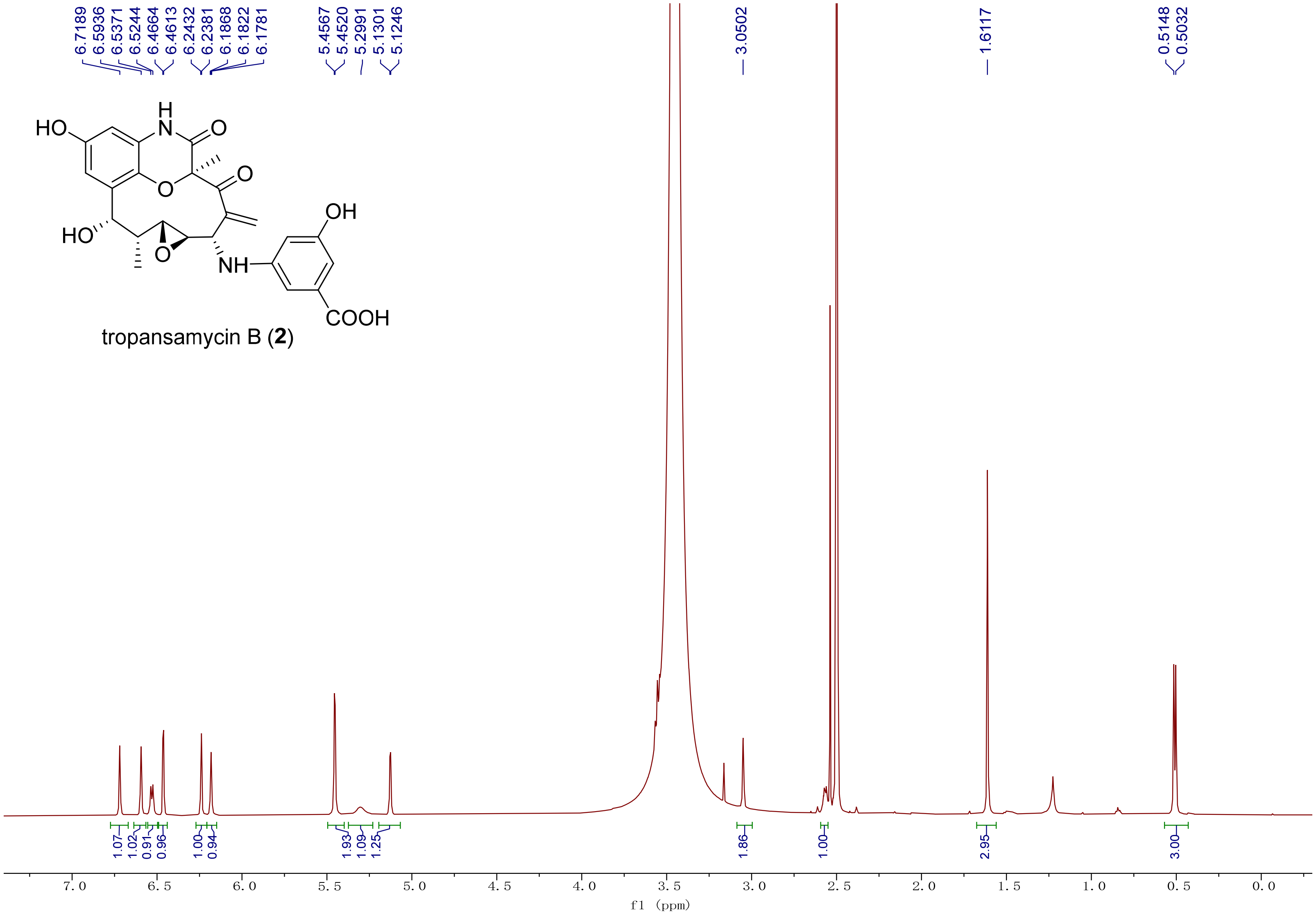

Figure S29. ^{1}H NMR spectrum of **2** in DMSO-d_6.

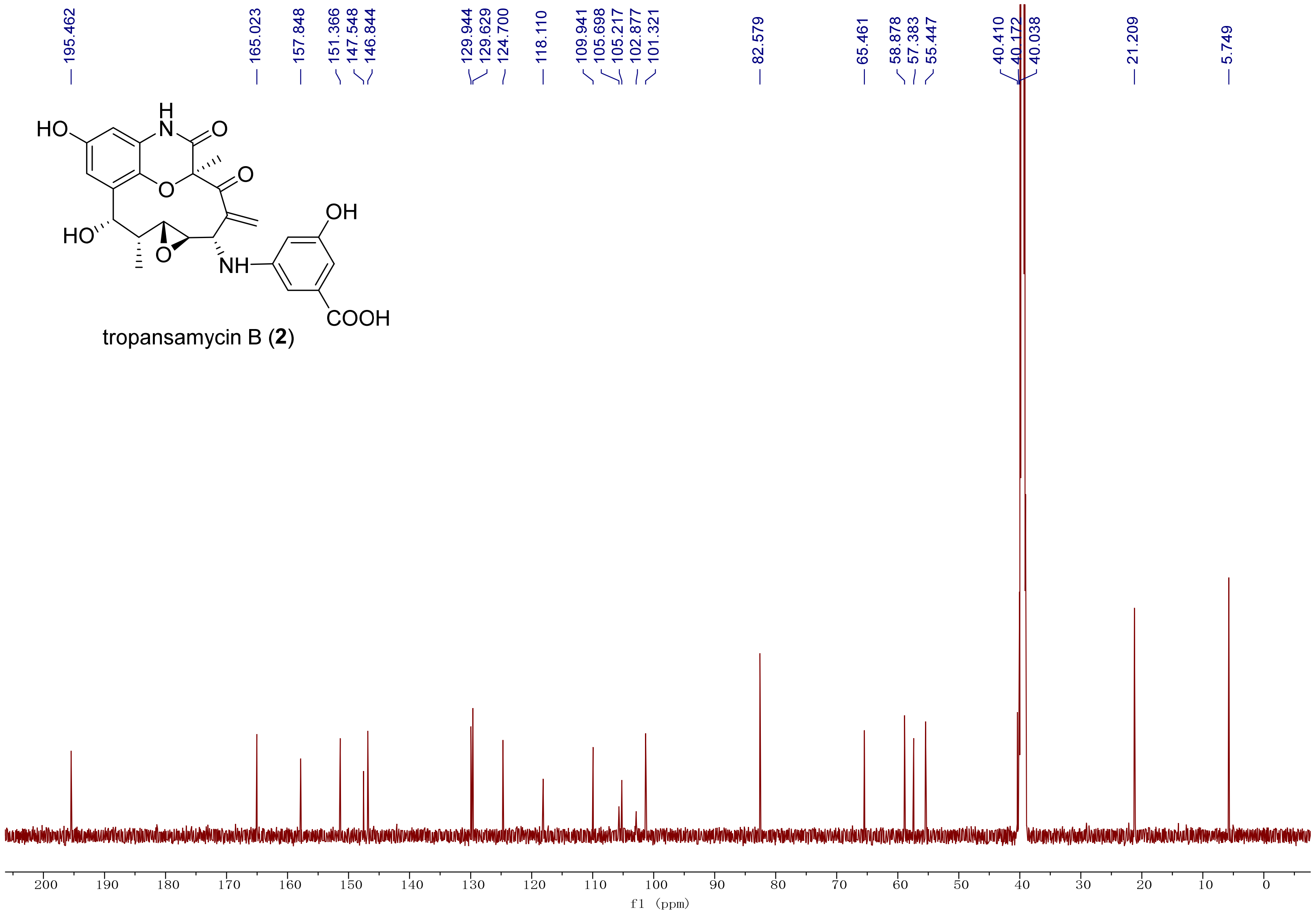

Figure S30. ^{13}C NMR spectrum of **2** in DMSO-d_6.

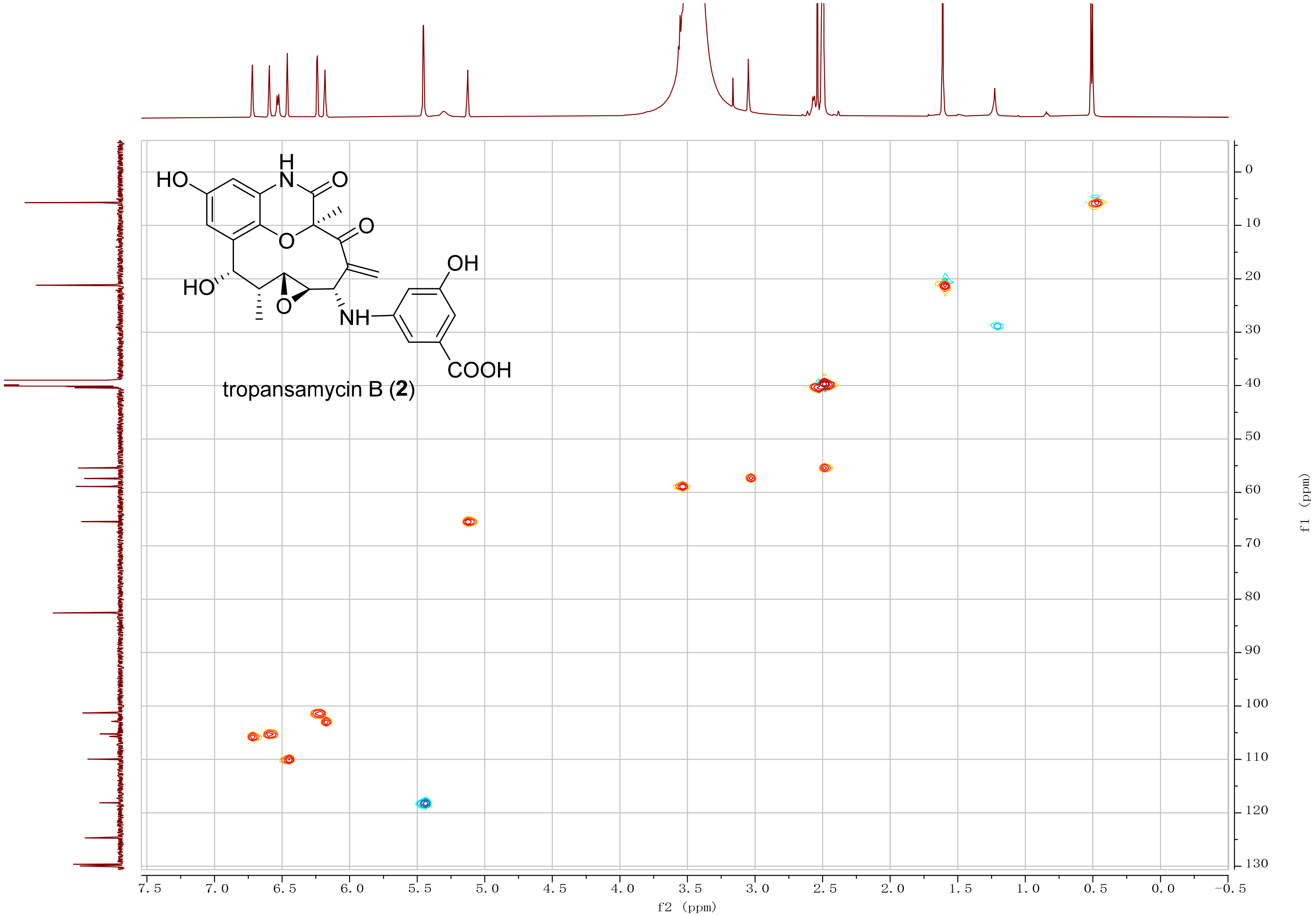

Figure S31. The HSQC spectrum of **2** in DMSO-d_6.

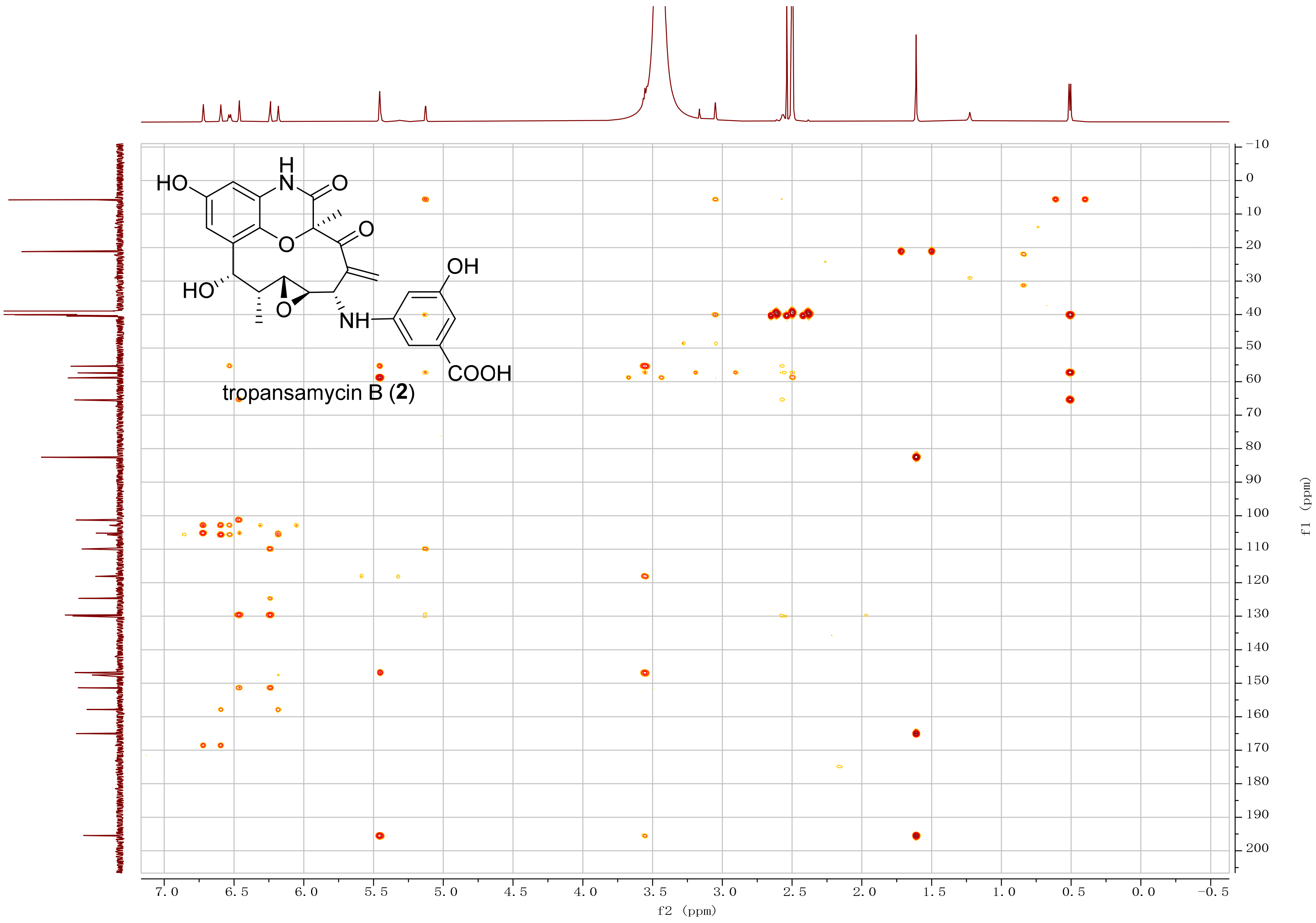

Figure S32. The HMBC spectrum of **2** in DMSO-d_6.

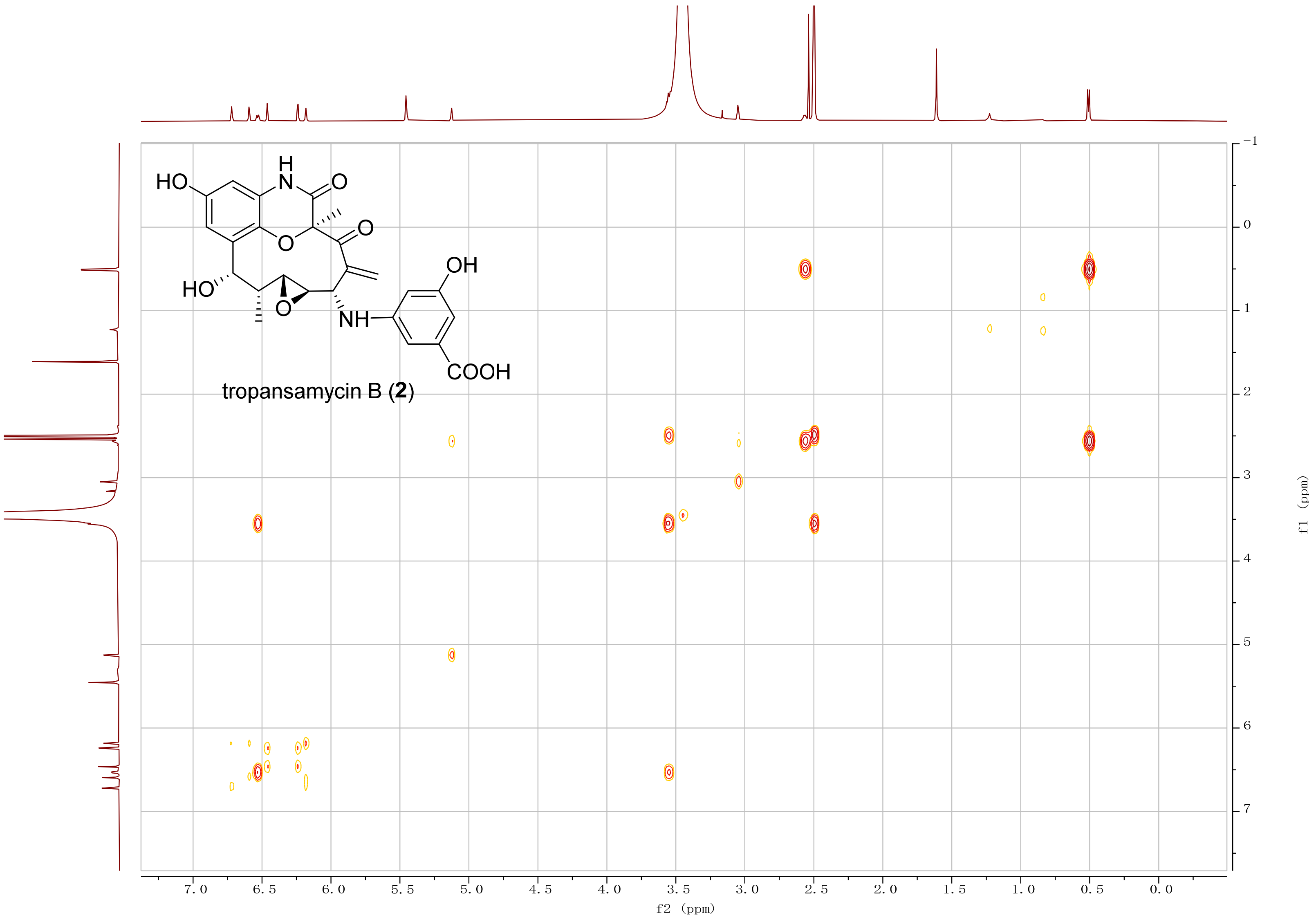

Figure S33. The ^{1}H-^{1}H COSY spectrum of **2** in DMSO-d_6.

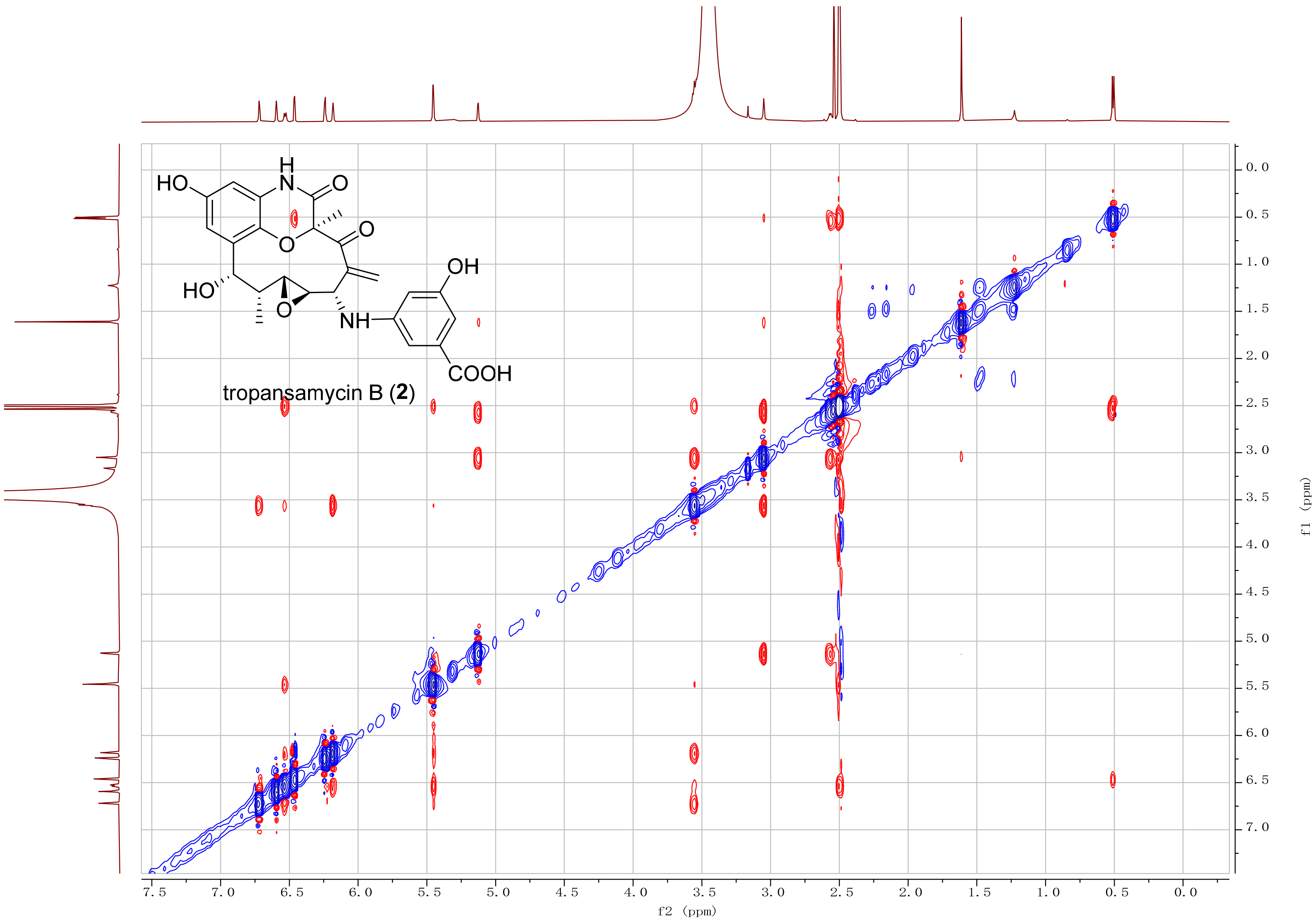

Figure S34. The ROESY spectrum of **2** in DMSO-d_6.

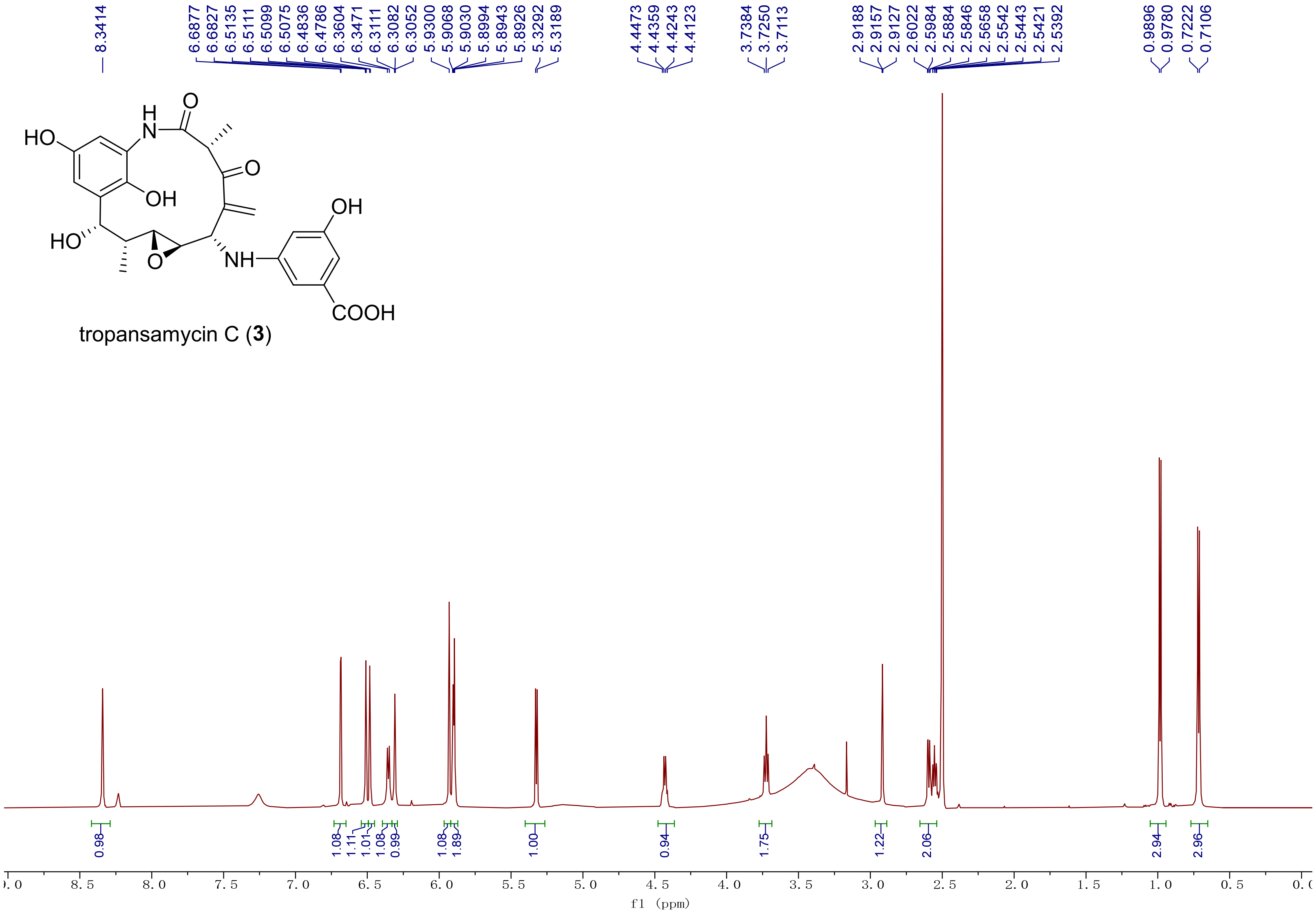

Figure S35. ^{1}H NMR spectrum of **3** in DMSO-d_6.

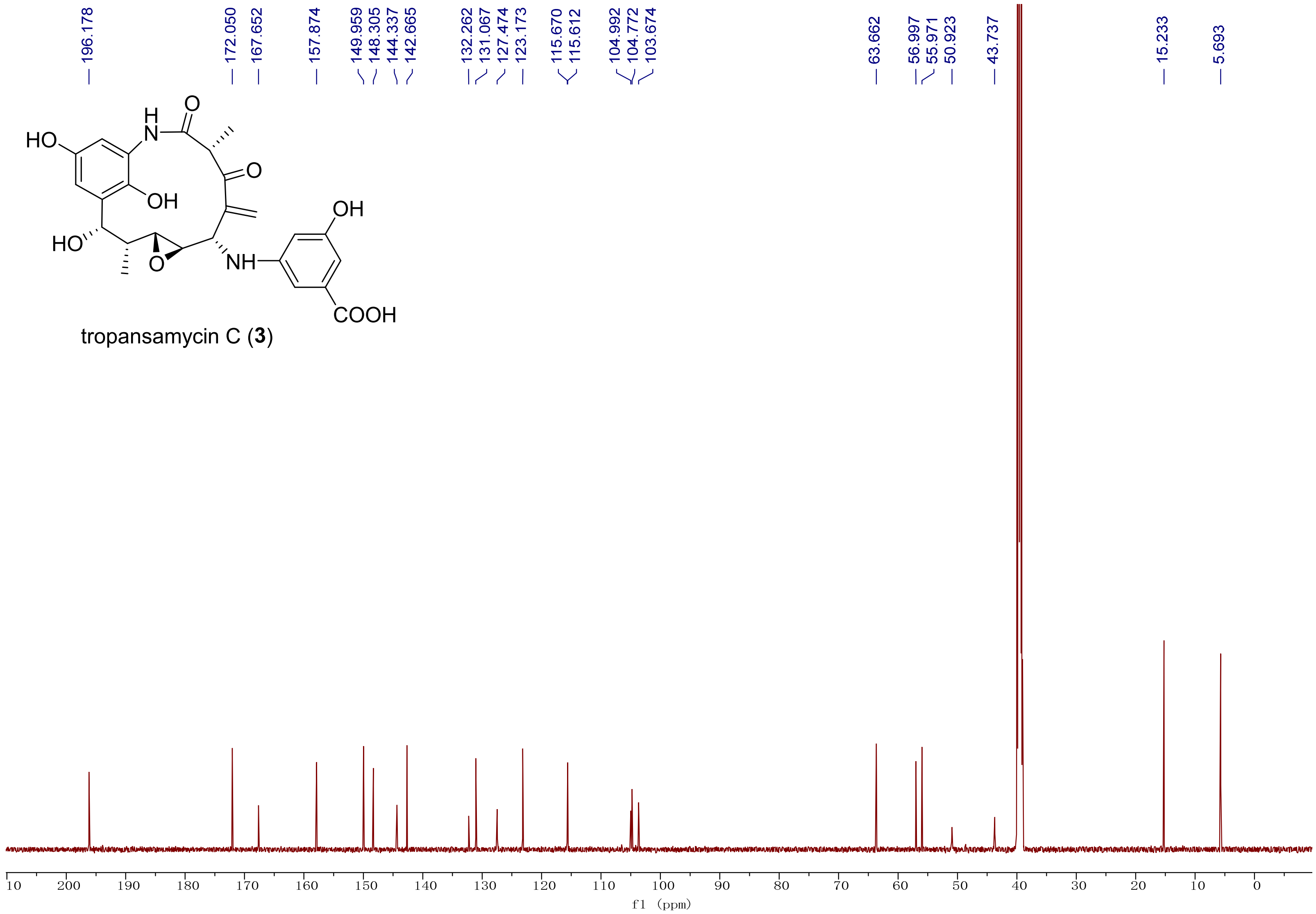

Figure S36. ^{13}C NMR spectrum of **3** in DMSO-d_6.

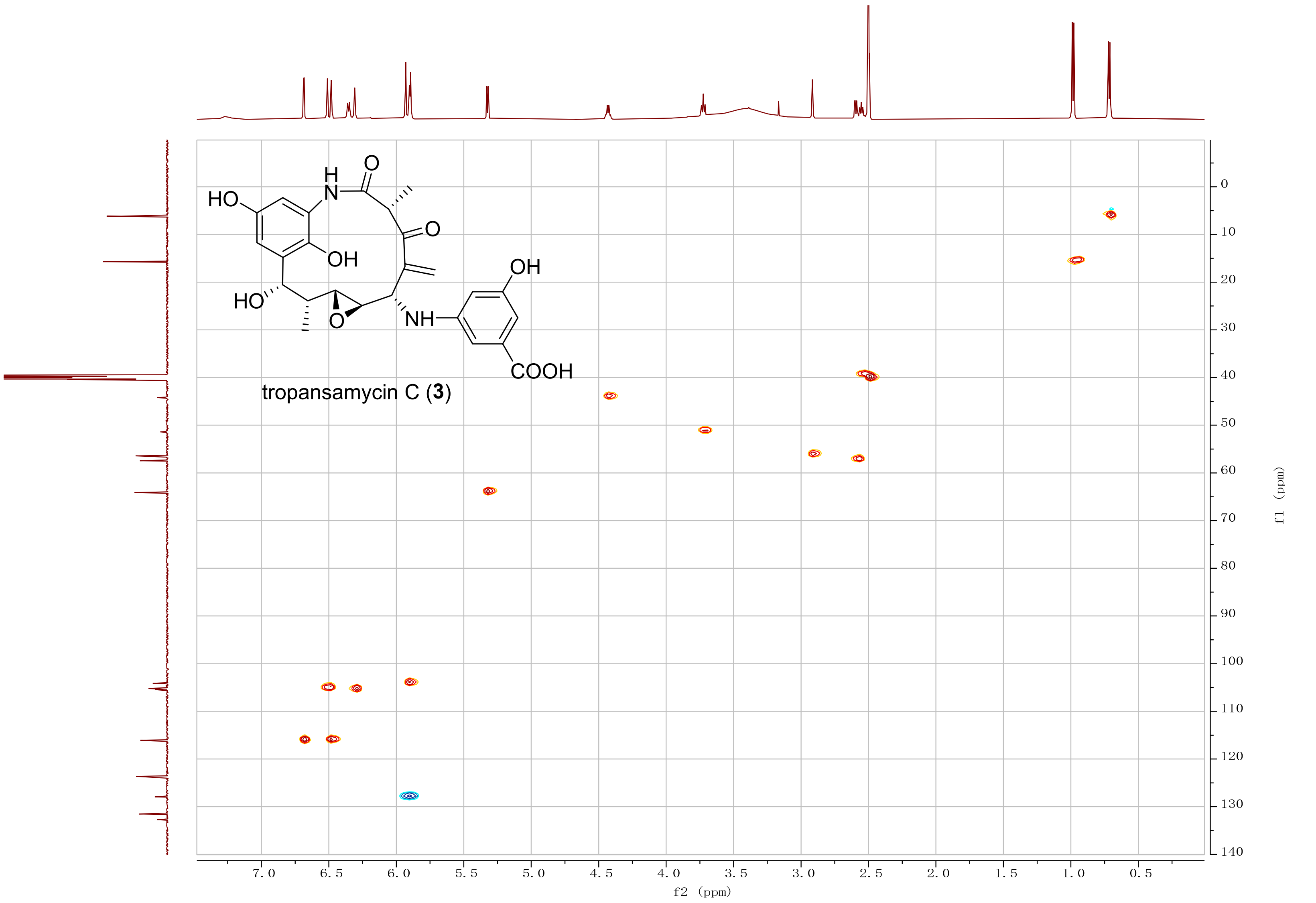

Figure S37. The HSQC spectrum of **3** in DMSO-d_6.

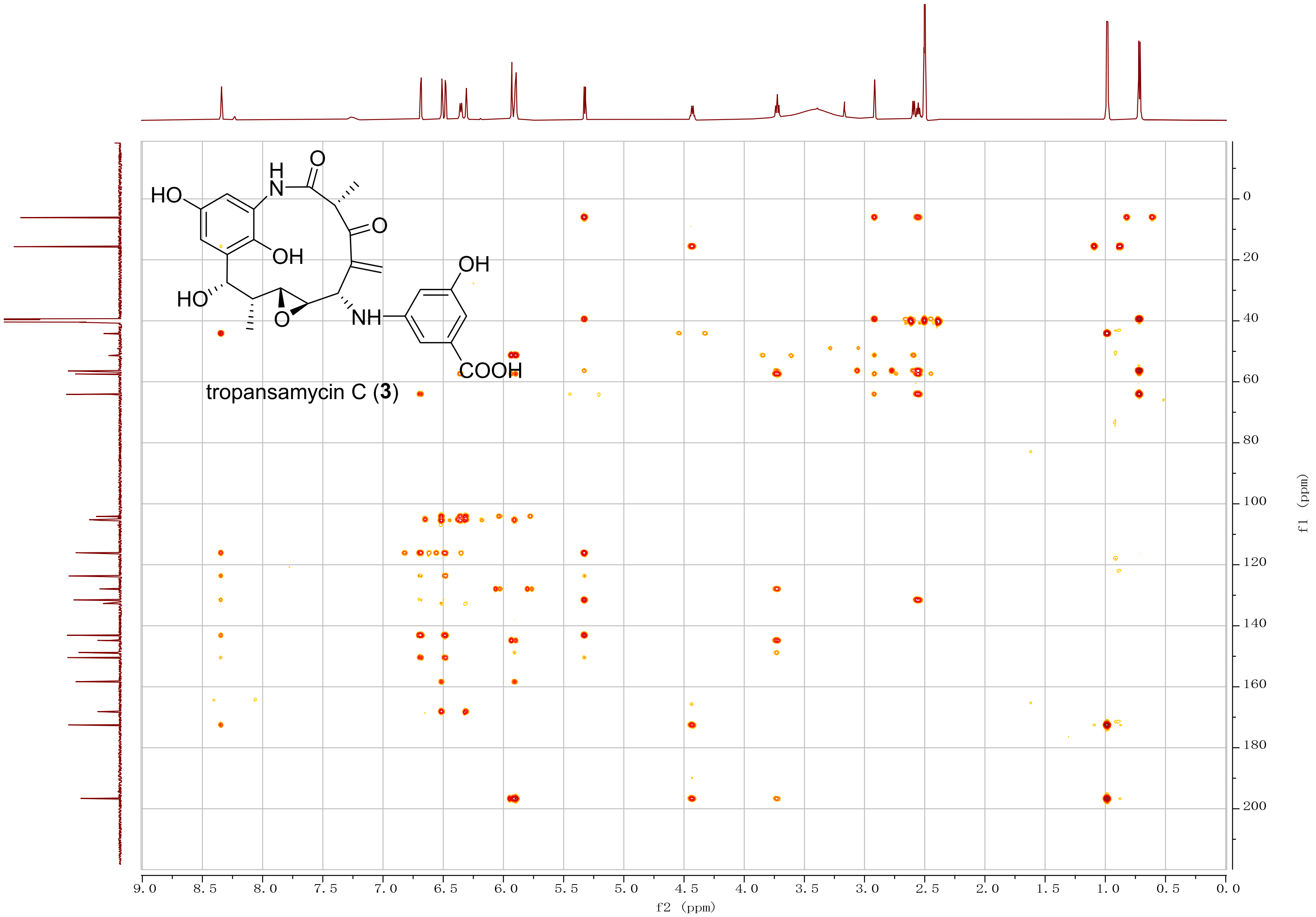

Figure S38. The HMBC spectrum of **3** in DMSO-d_6.

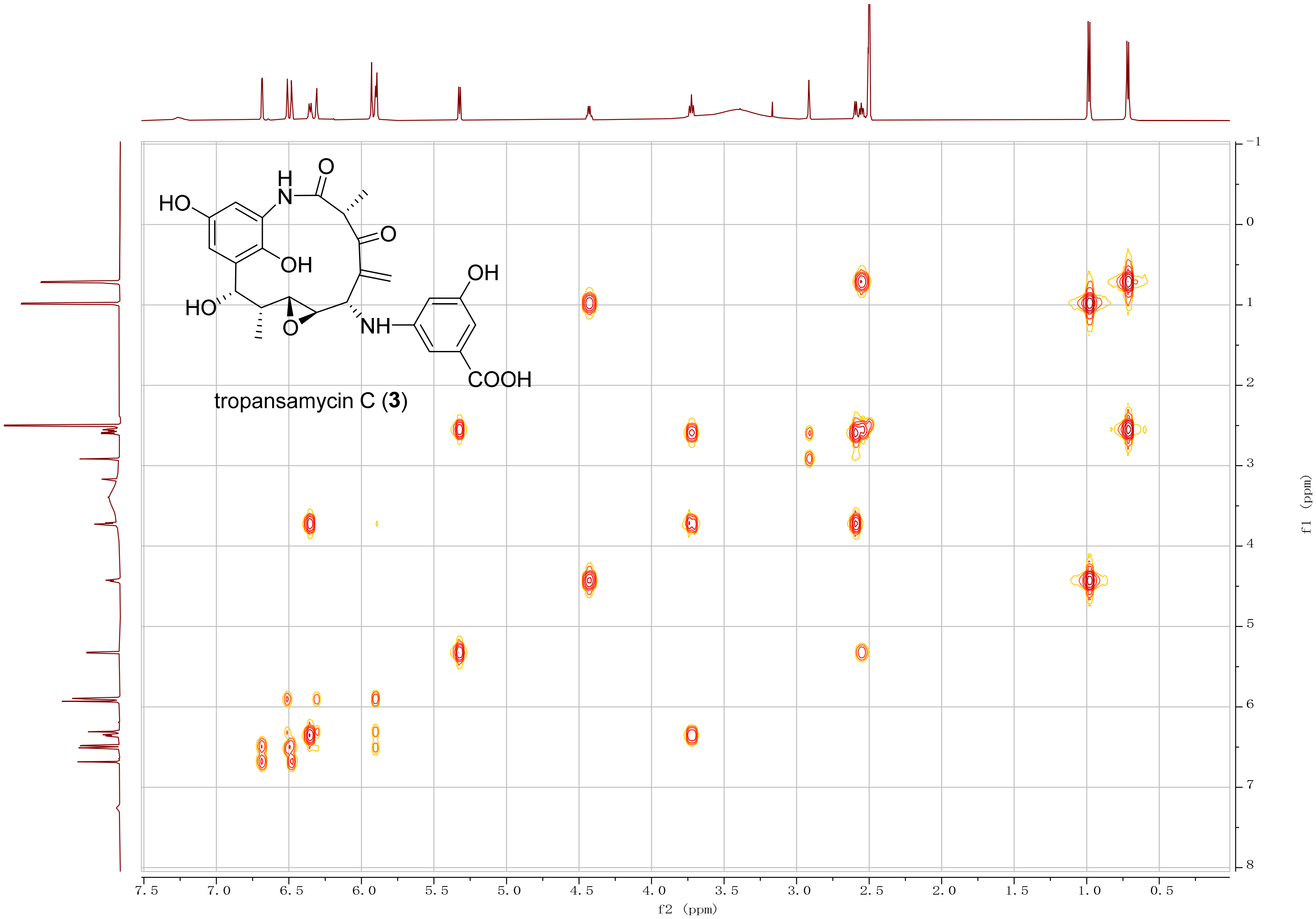

Figure S39. The ^{1}H-^{1}H COSY spectrum of **3** in DMSO-d_6.

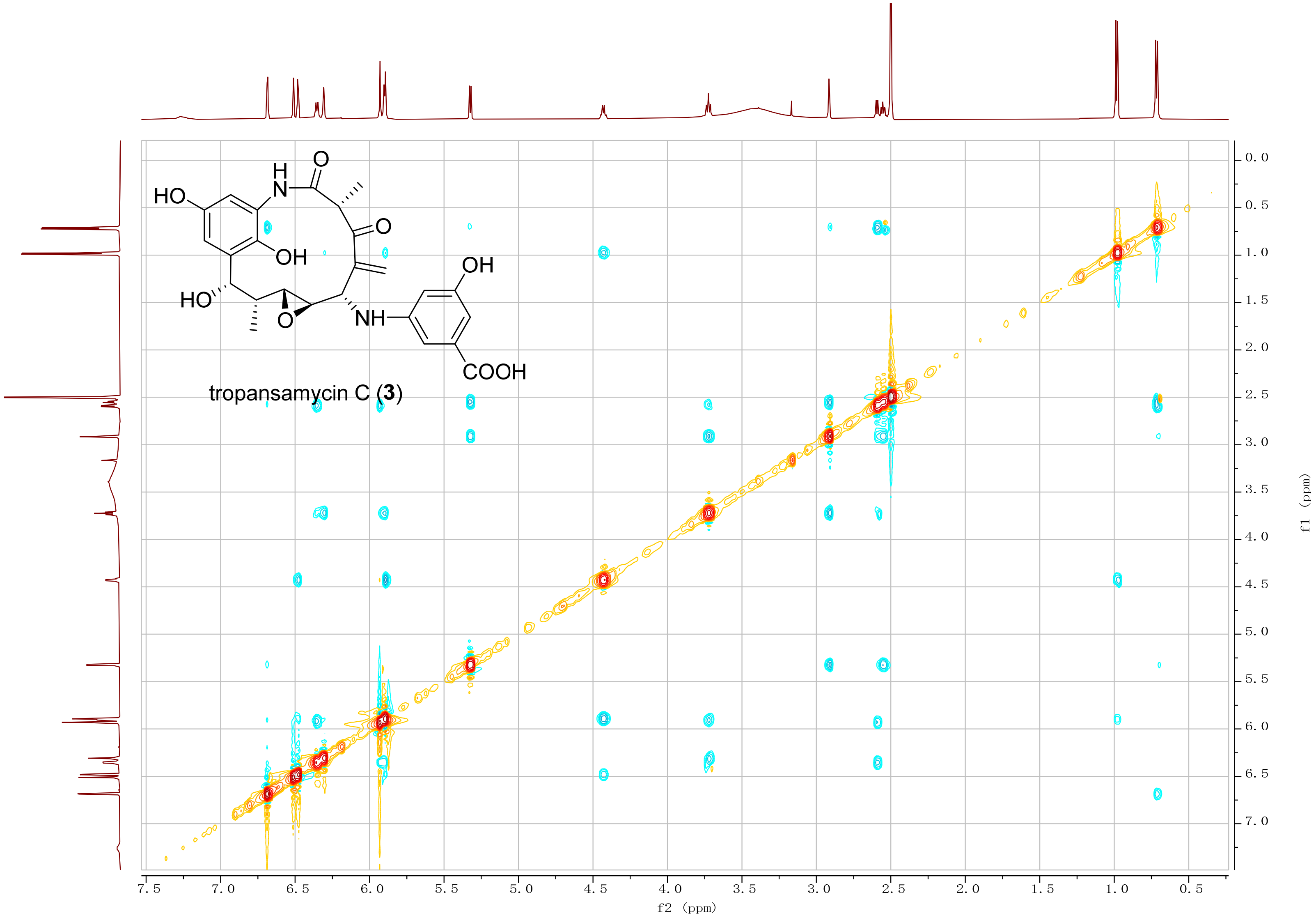

Figure S40. The ROESY spectrum of **3** in DMSO-d_6.

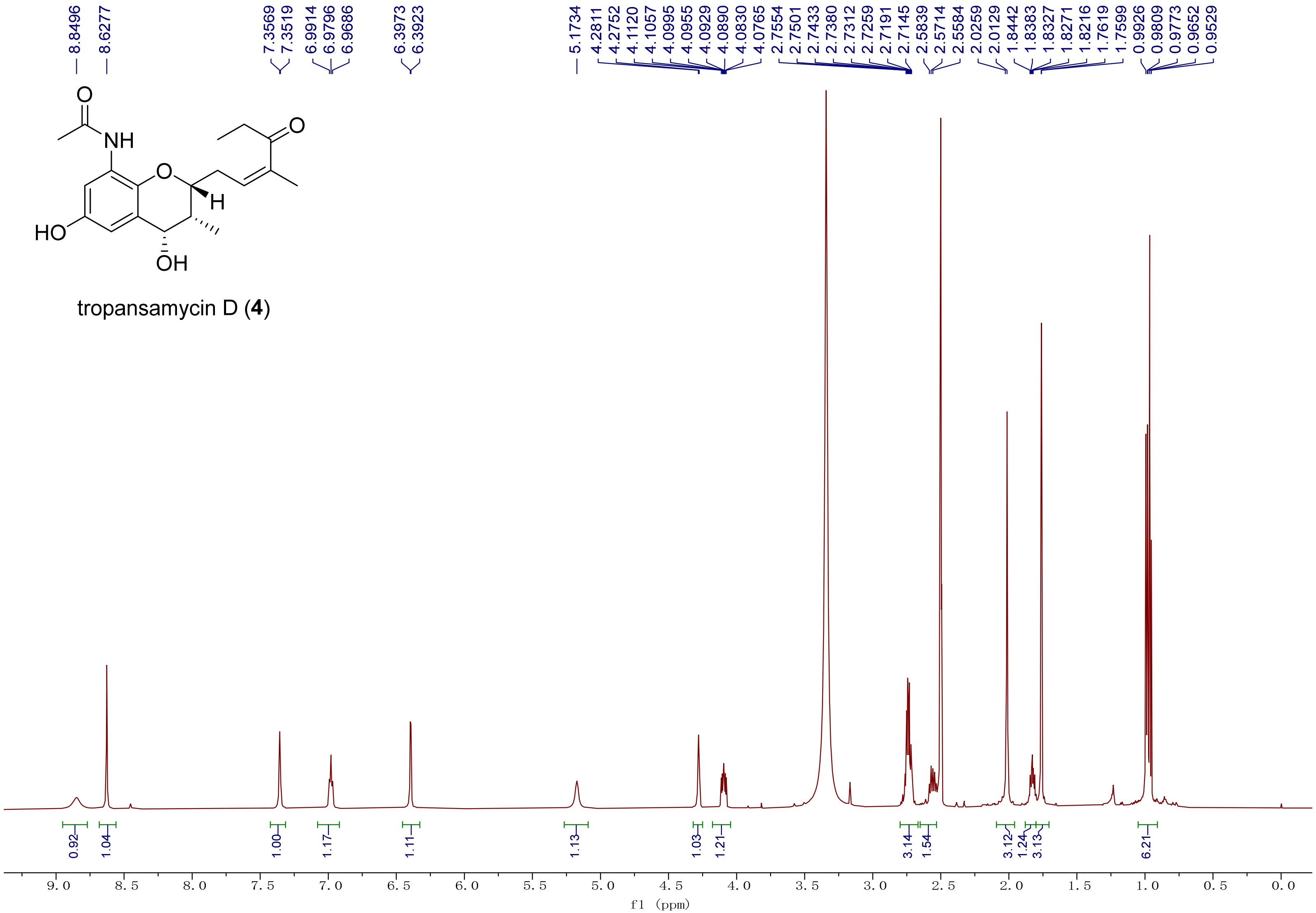

Figure S41. ^{1}H NMR spectrum of **4** in DMSO-d_6.

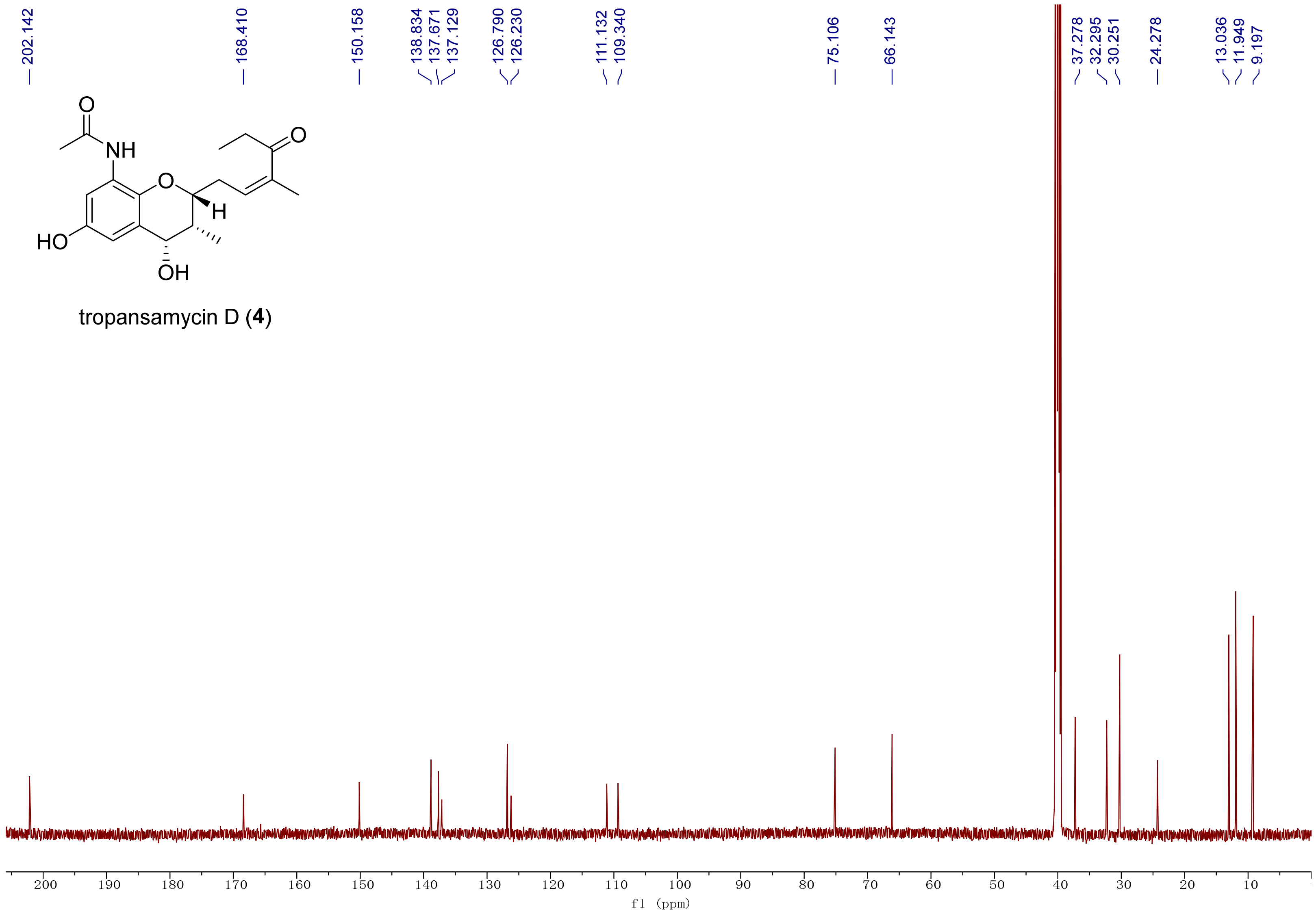

Figure S42. ^{13}C NMR spectrum of **4** in DMSO-d_6.

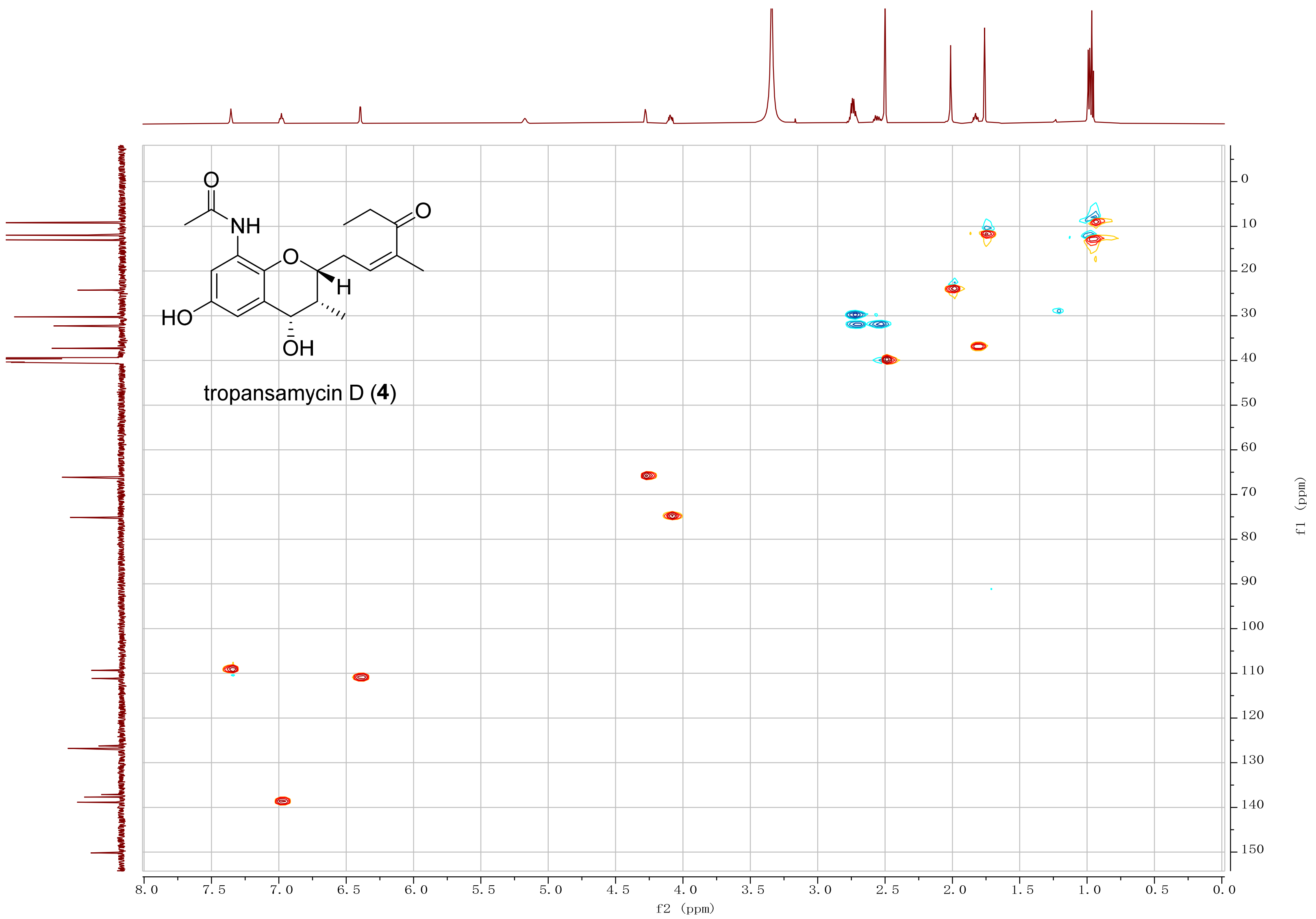

Figure S43. The HSQC spectrum of **4** in DMSO-d_6.

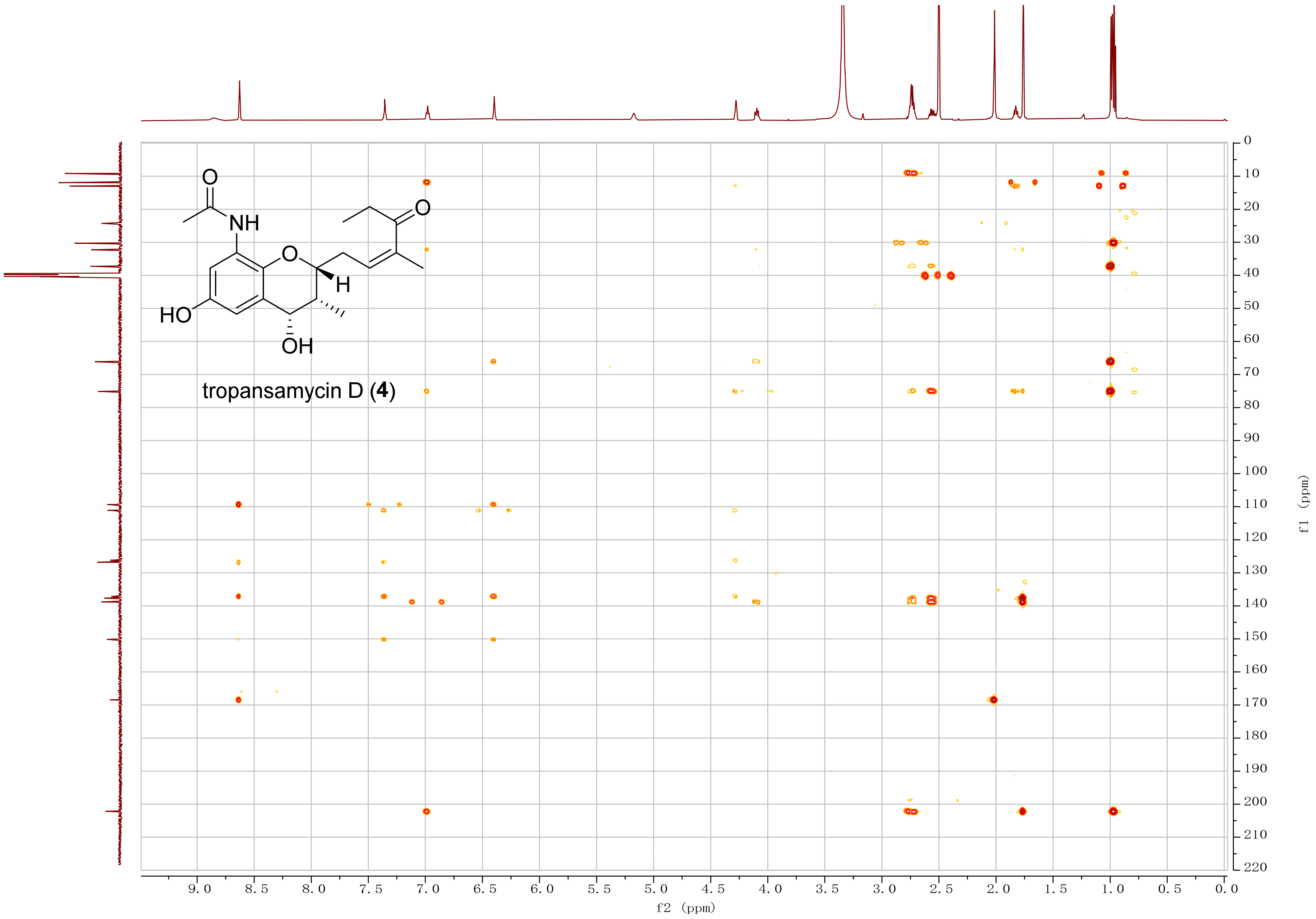

Figure S44. The HMBC spectrum of **4** in DMSO-d_6.

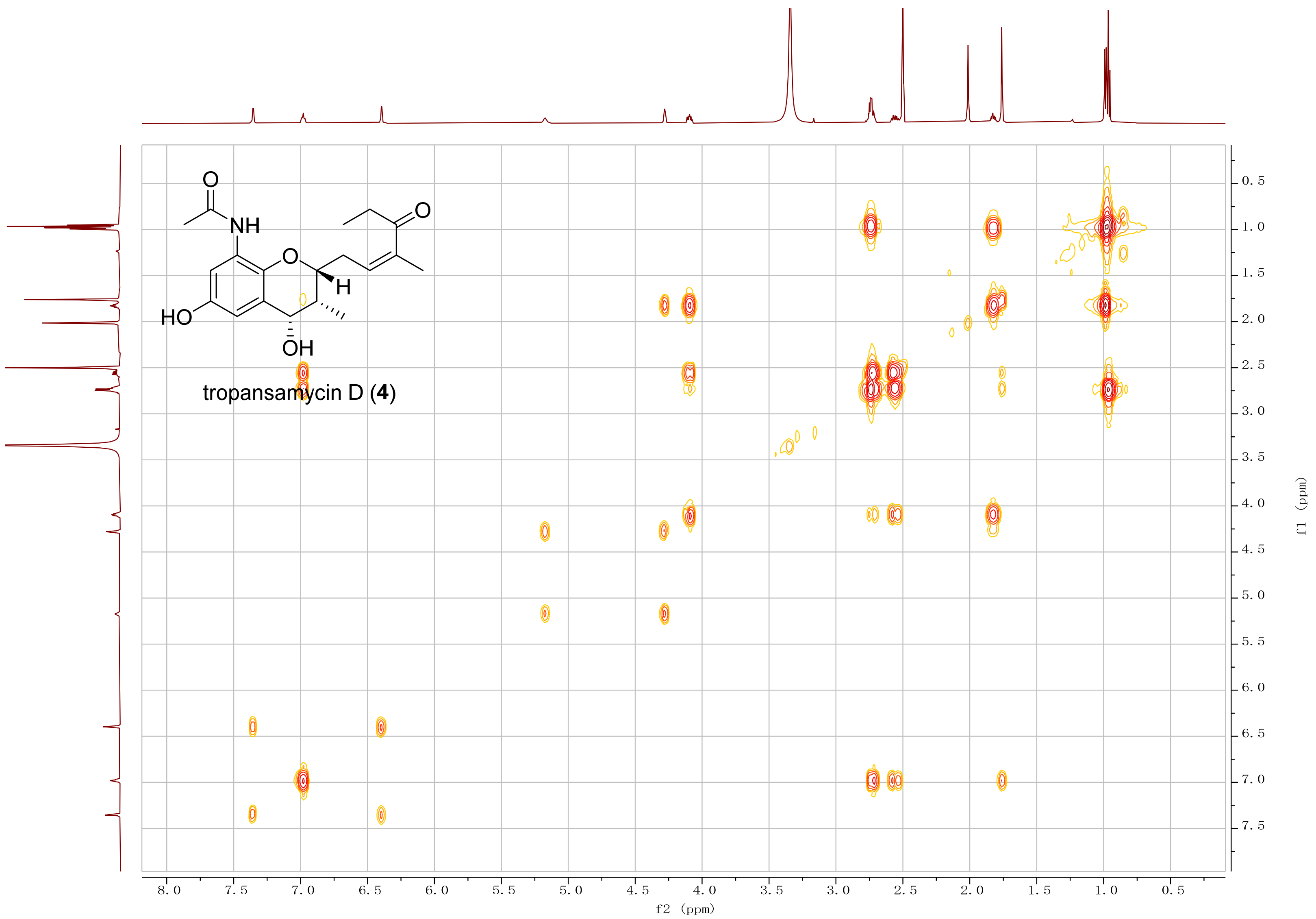

Figure S45. The ^{1}H-^{1}H COSY spectrum of **4** in DMSO-d_6.

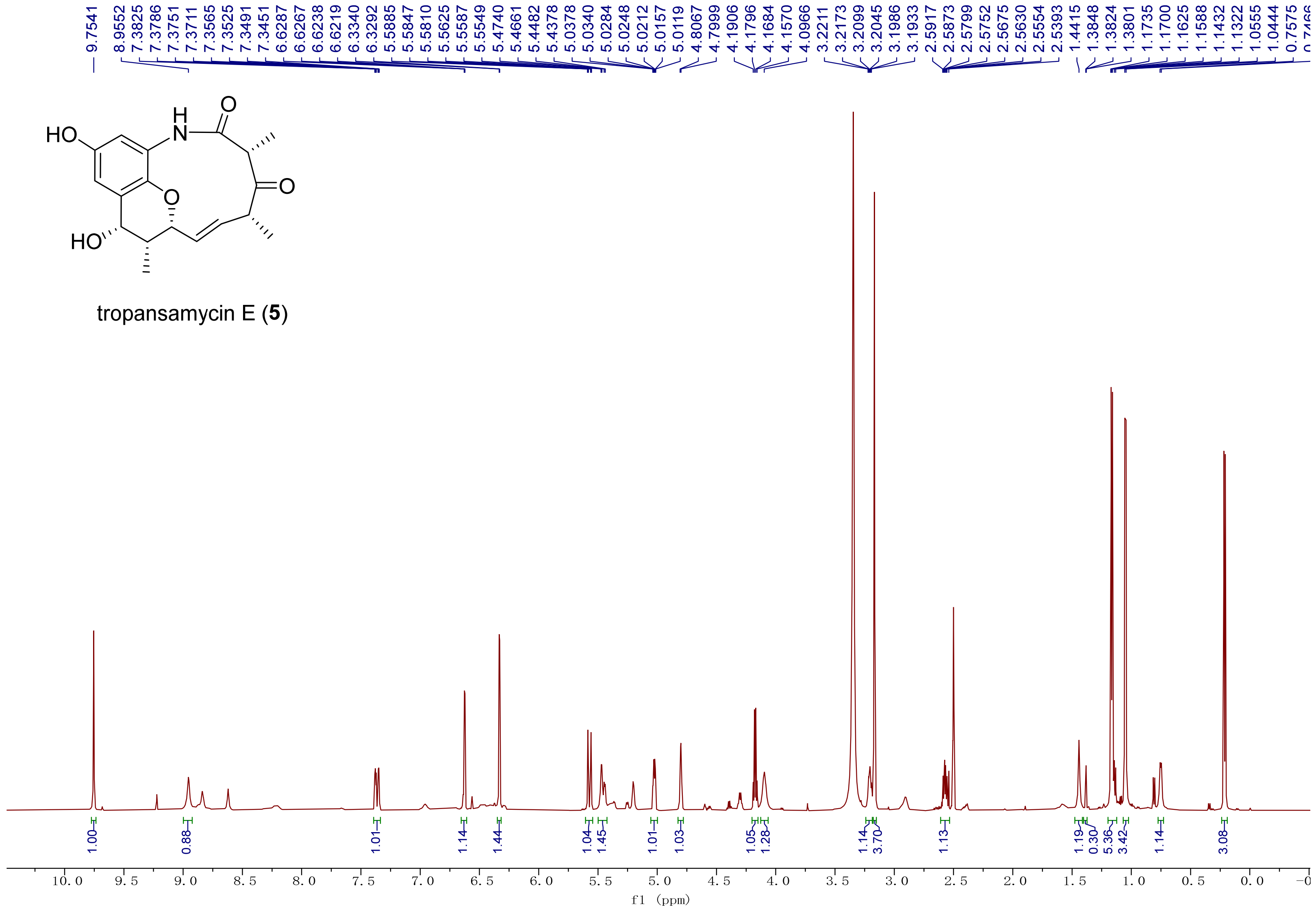

Figure S46. ^{1}H NMR spectrum of **5** in DMSO-d_6.

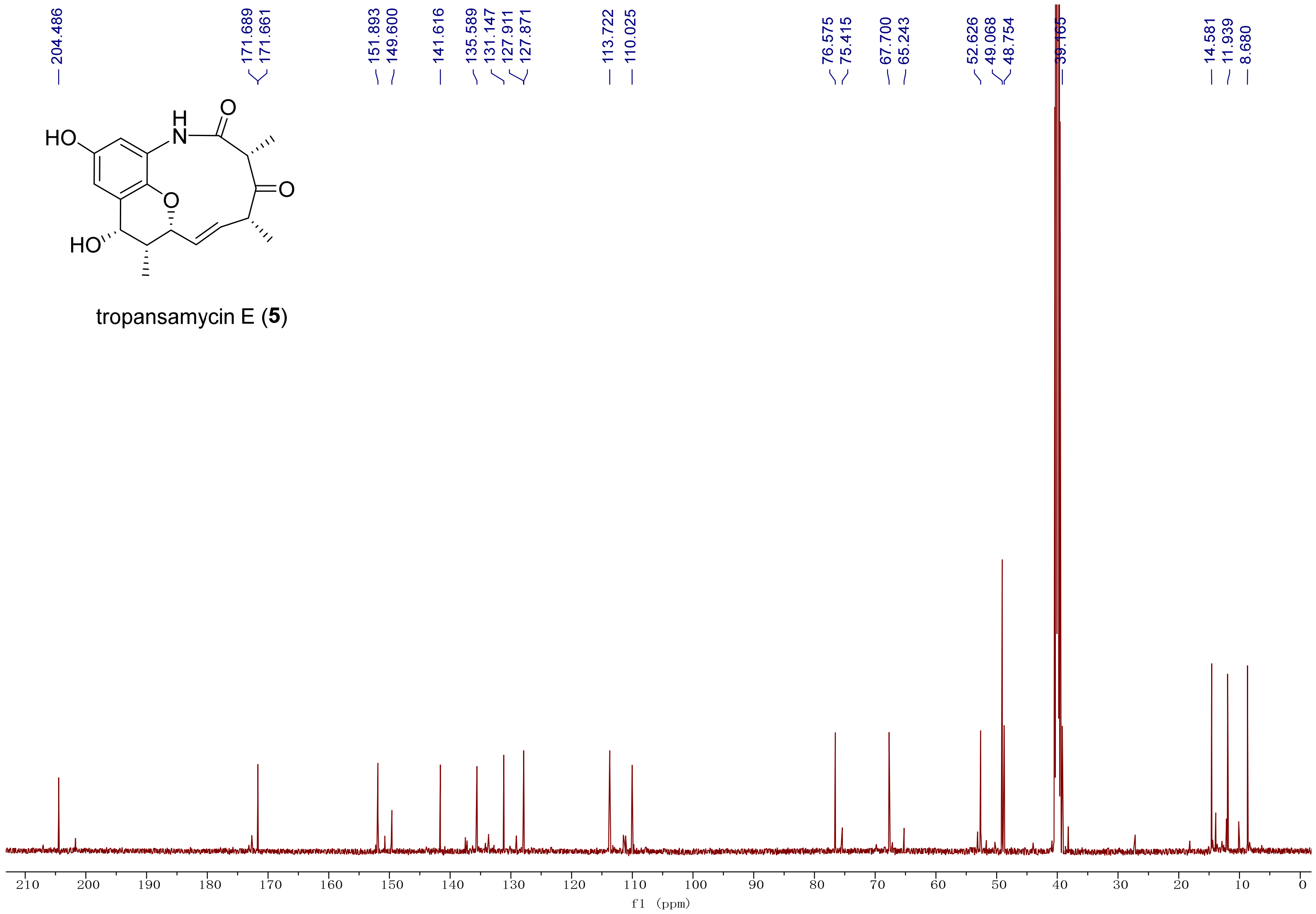

Figure S47. ^{13}C NMR spectrum of **5** in DMSO-d_6.

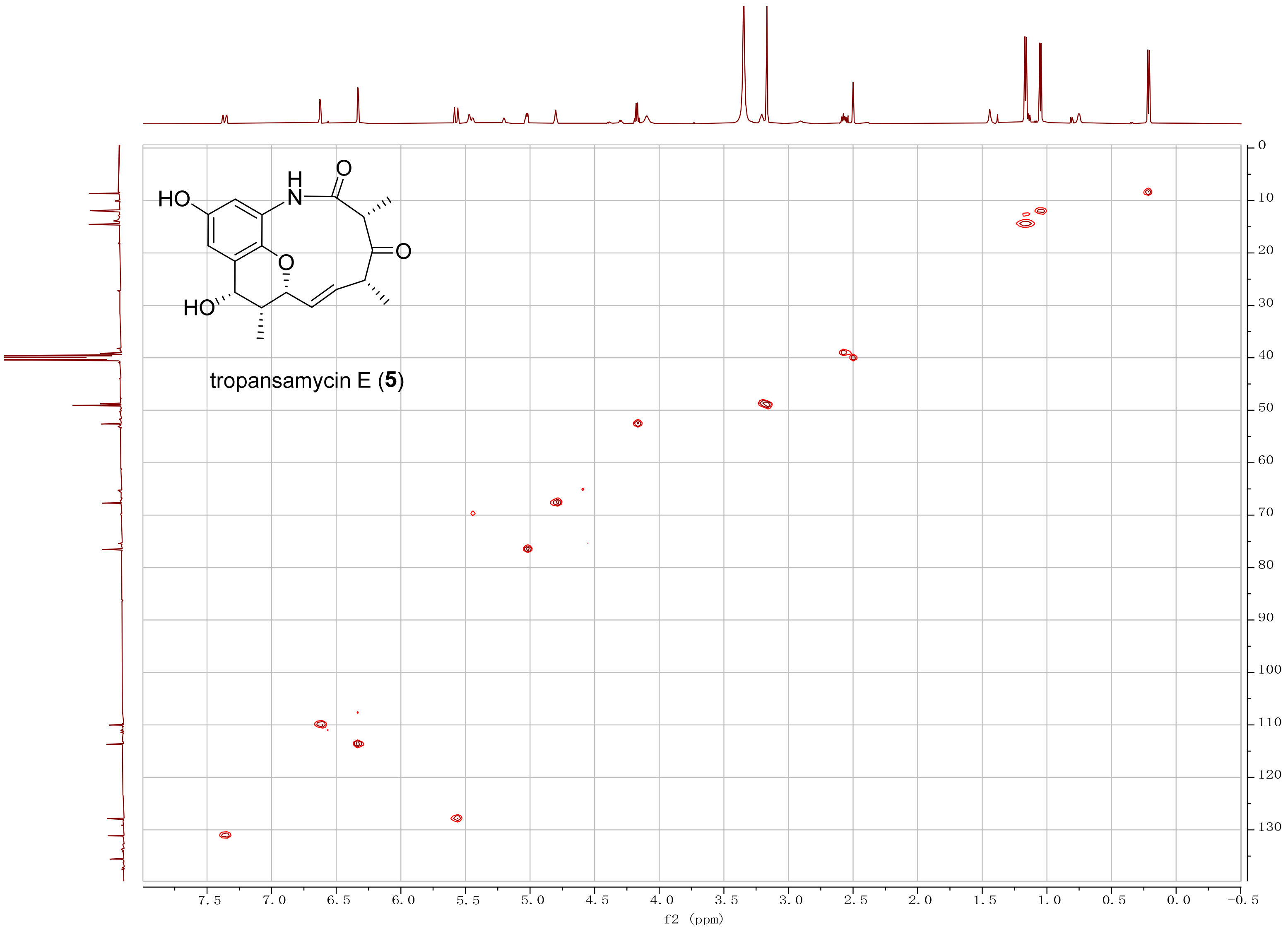

Figure S48. The HSQC spectrum of **5** in DMSO-d_6.

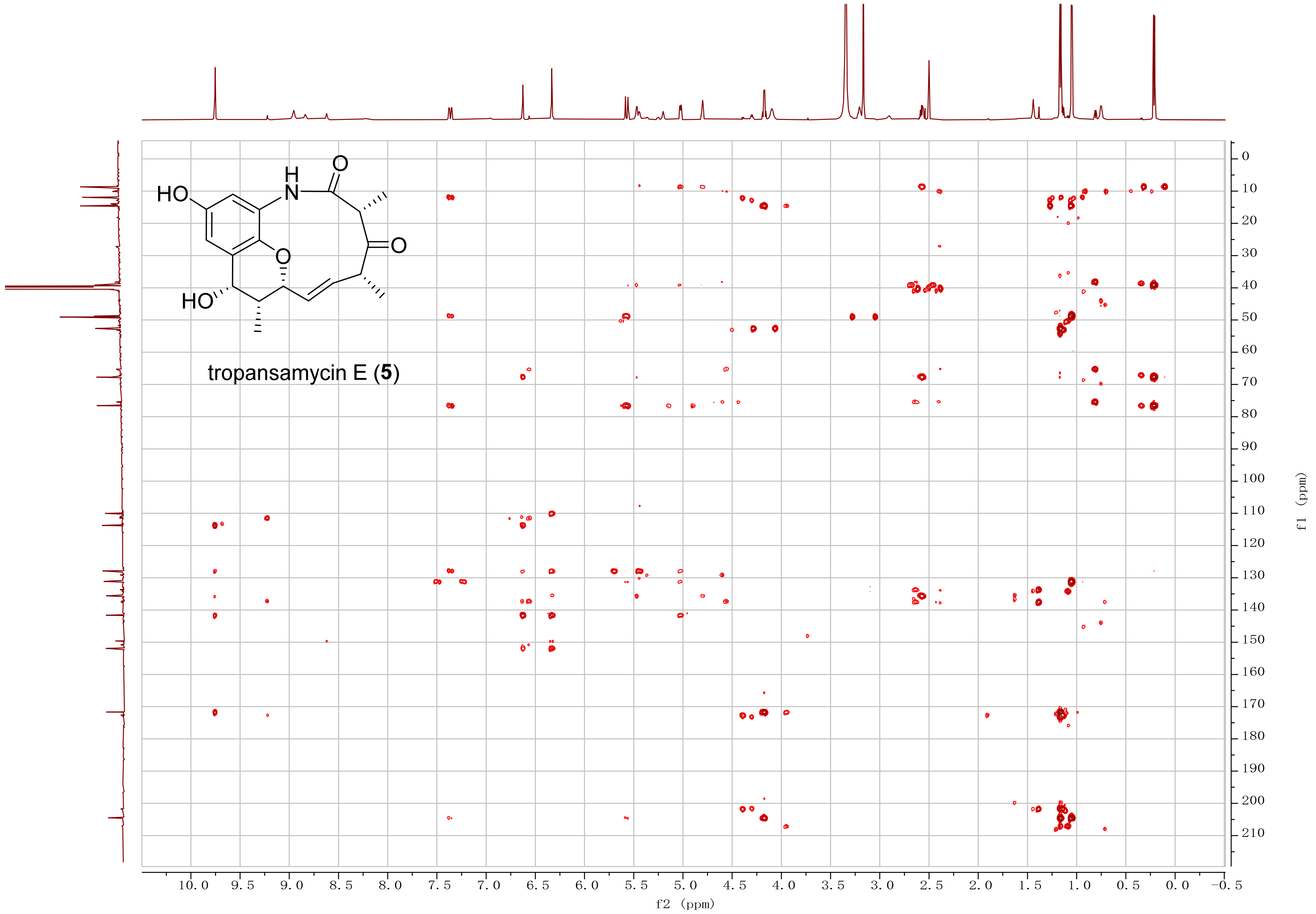

Figure S49. The HMBC spectrum of **5** in DMSO-d_6.

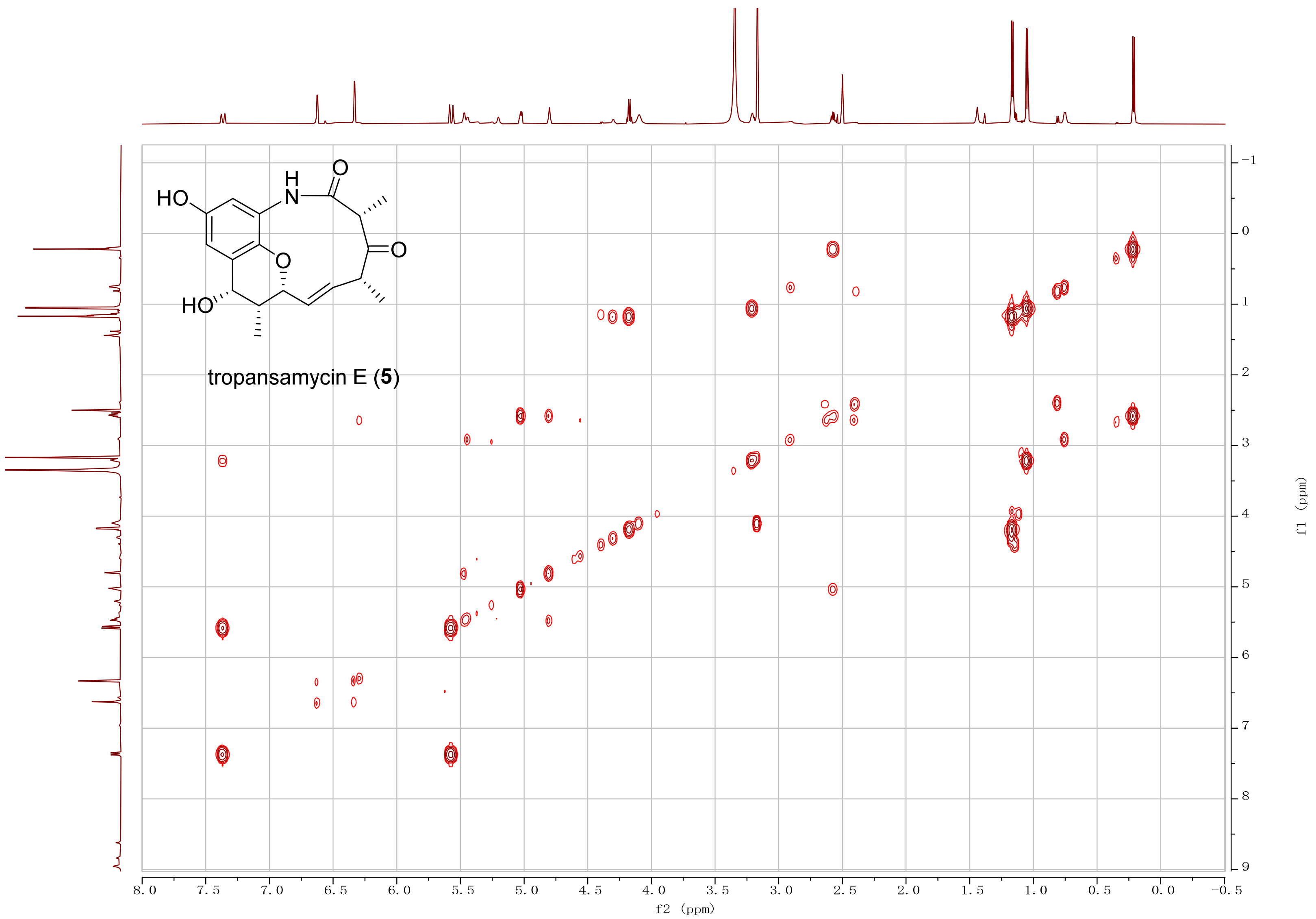

Figure S50. The ^{1}H-^{1}H COSY spectrum of **5** in DMSO-d_6.

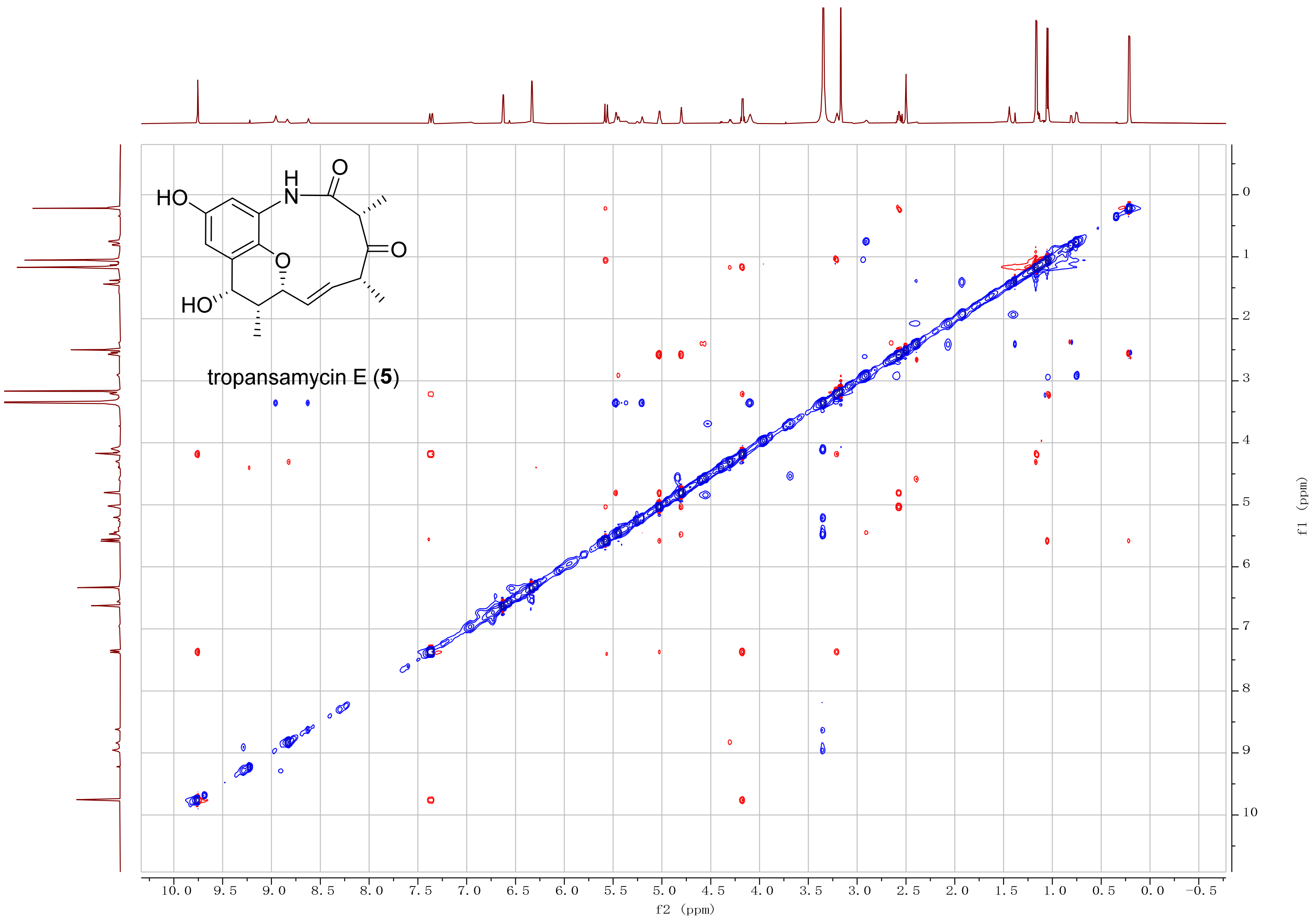

Figure S51. The ROESY spectrum of **5** in DMSO-d_6.

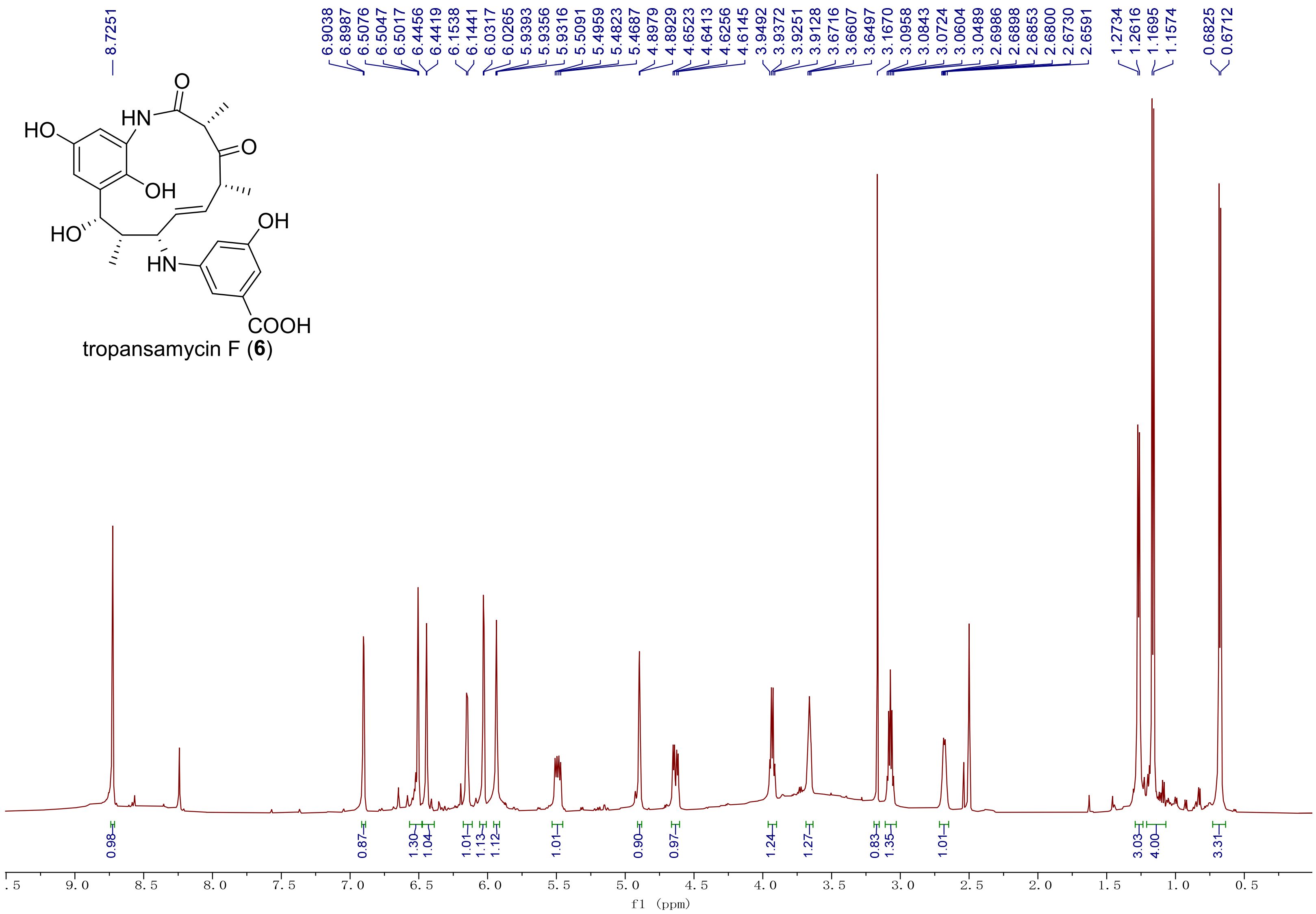

Figure S52. ^{1}H NMR spectrum of **6** in DMSO-d_6.

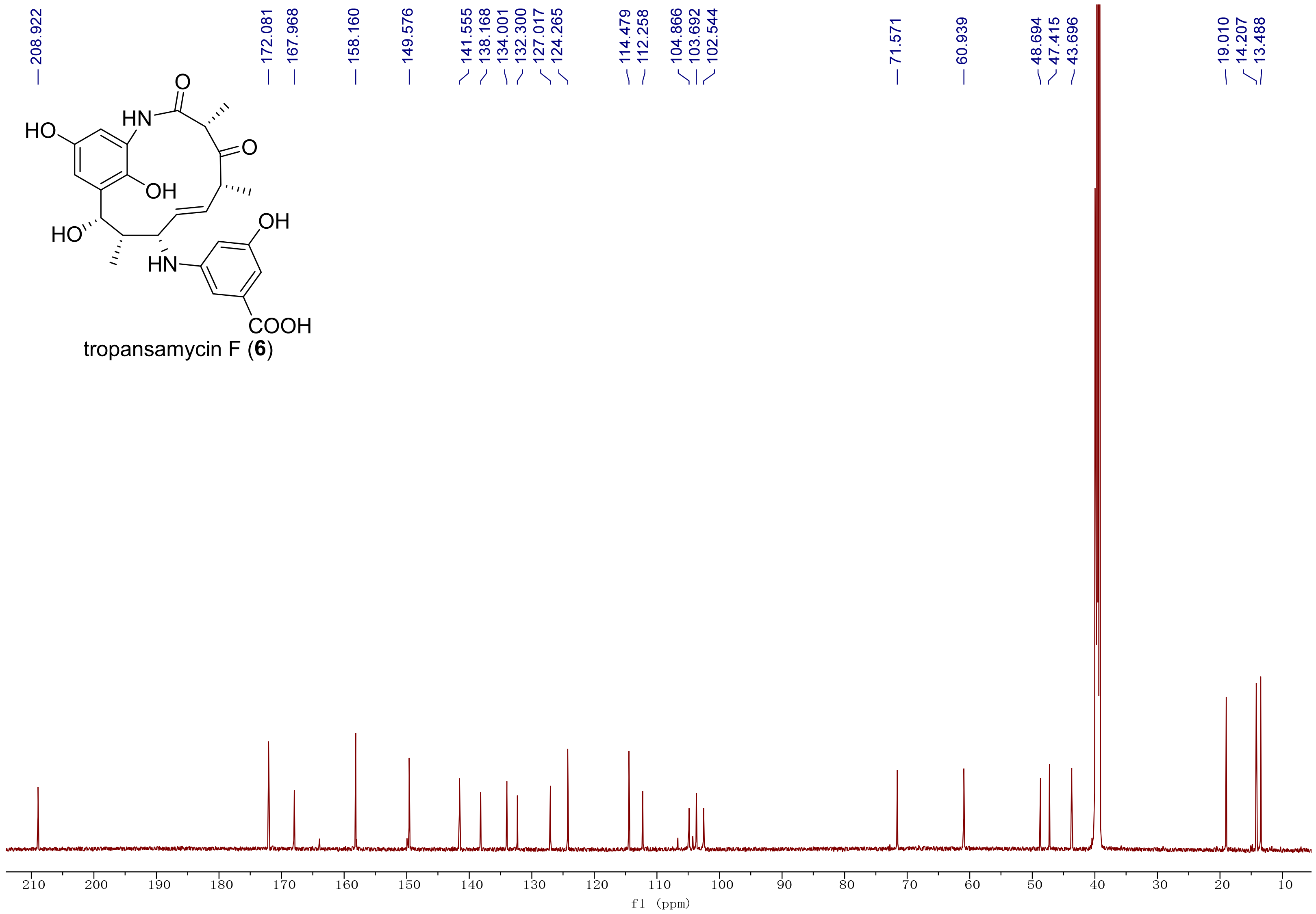

Figure S53. ^{13}C NMR spectrum of **6** in DMSO-d_6.

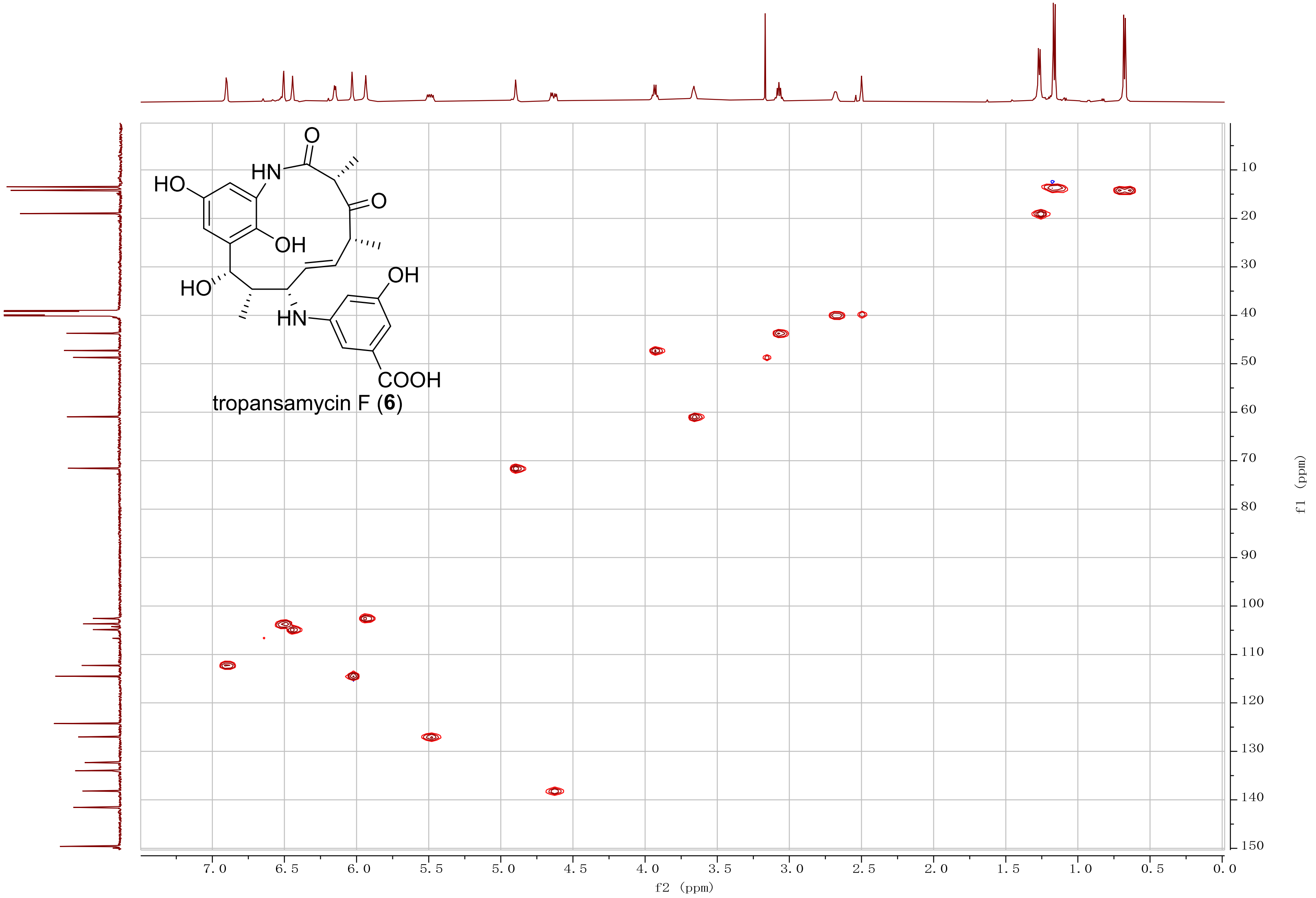

Figure S54. The HSQC spectrum of **6** in DMSO-d_6.

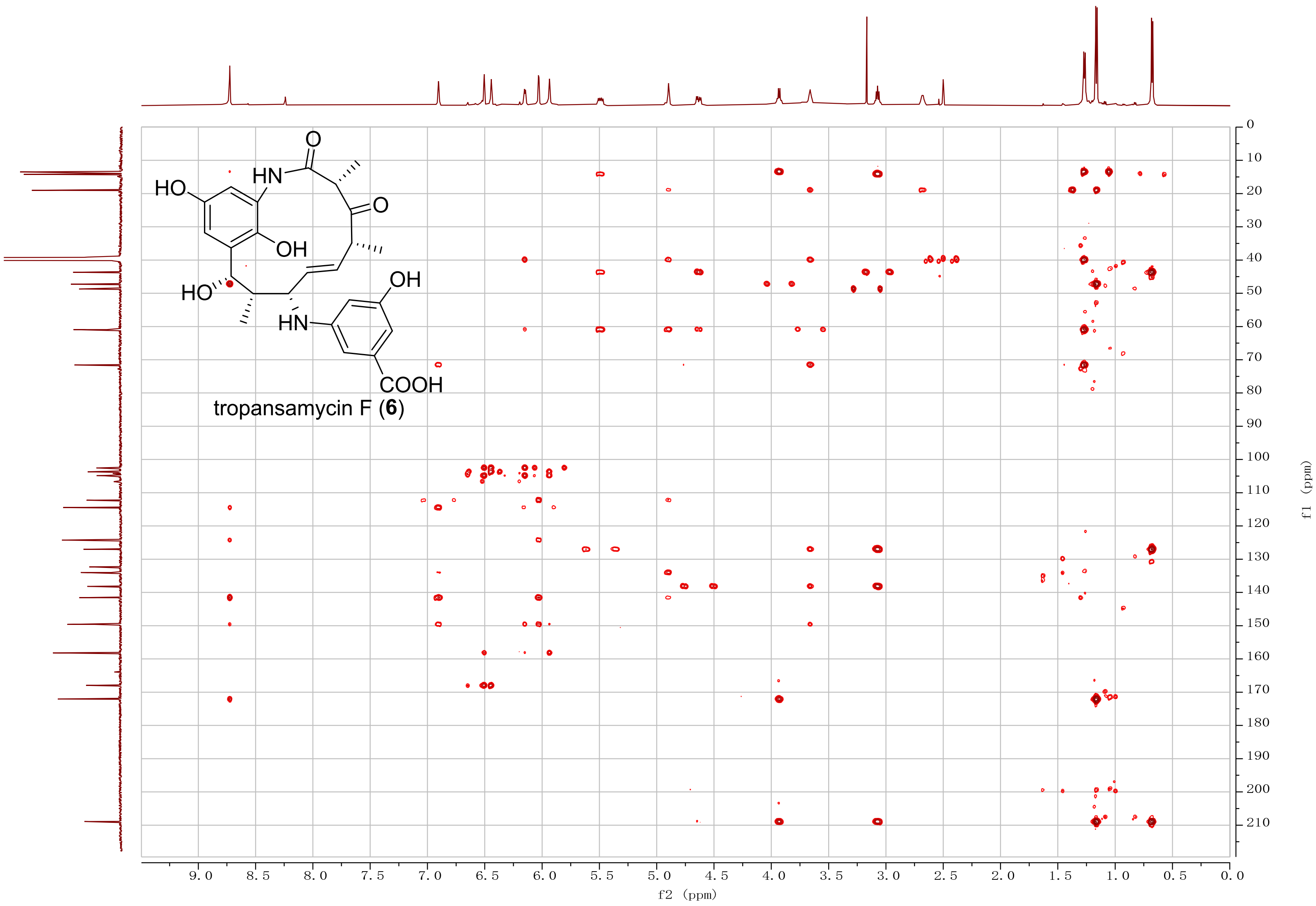

Figure S55. The HMBC spectrum of **6** in DMSO-d_6.

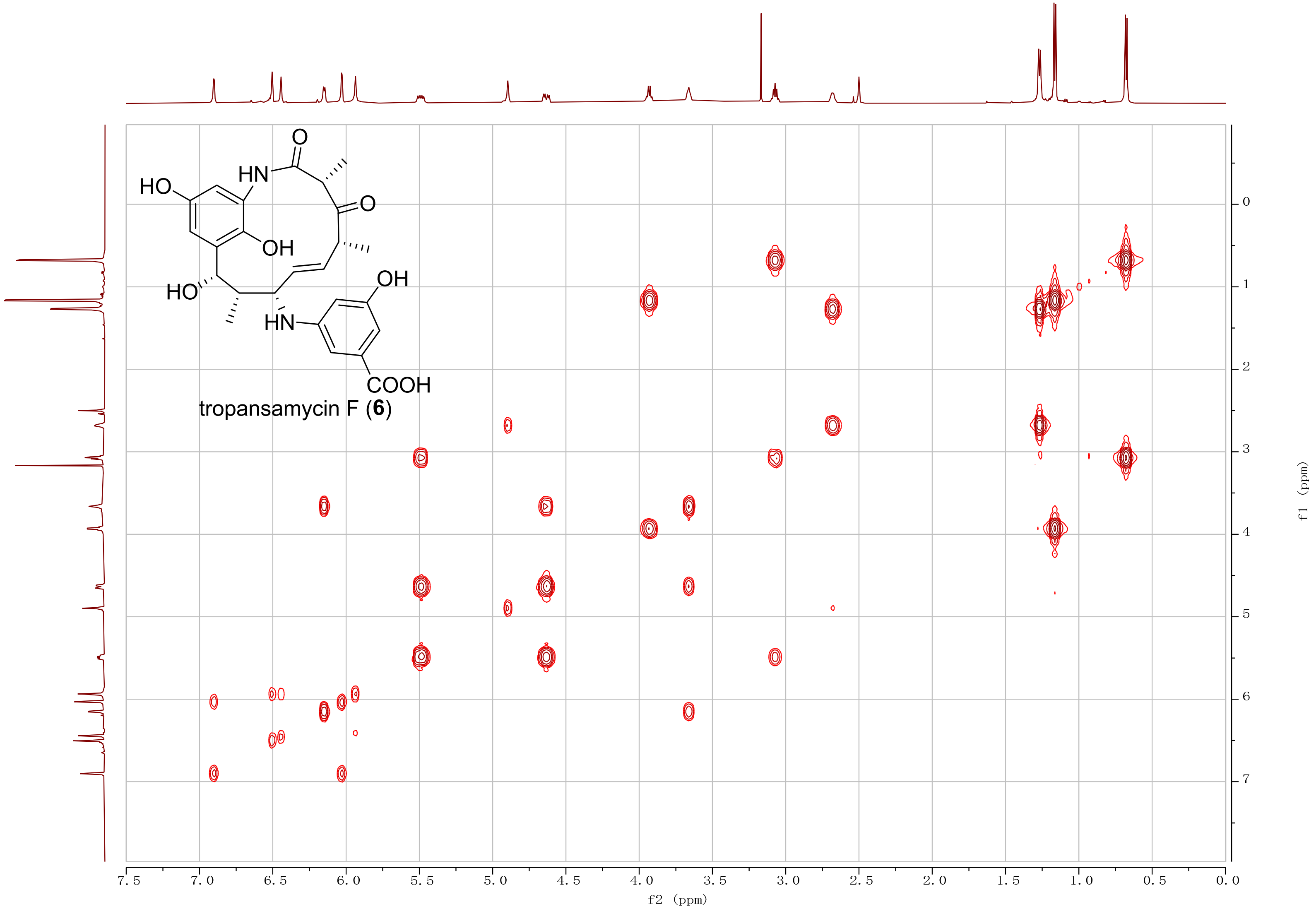

Figure S56. The ^{1}H-^{1}H COSY spectrum of **6** in DMSO-d_6.

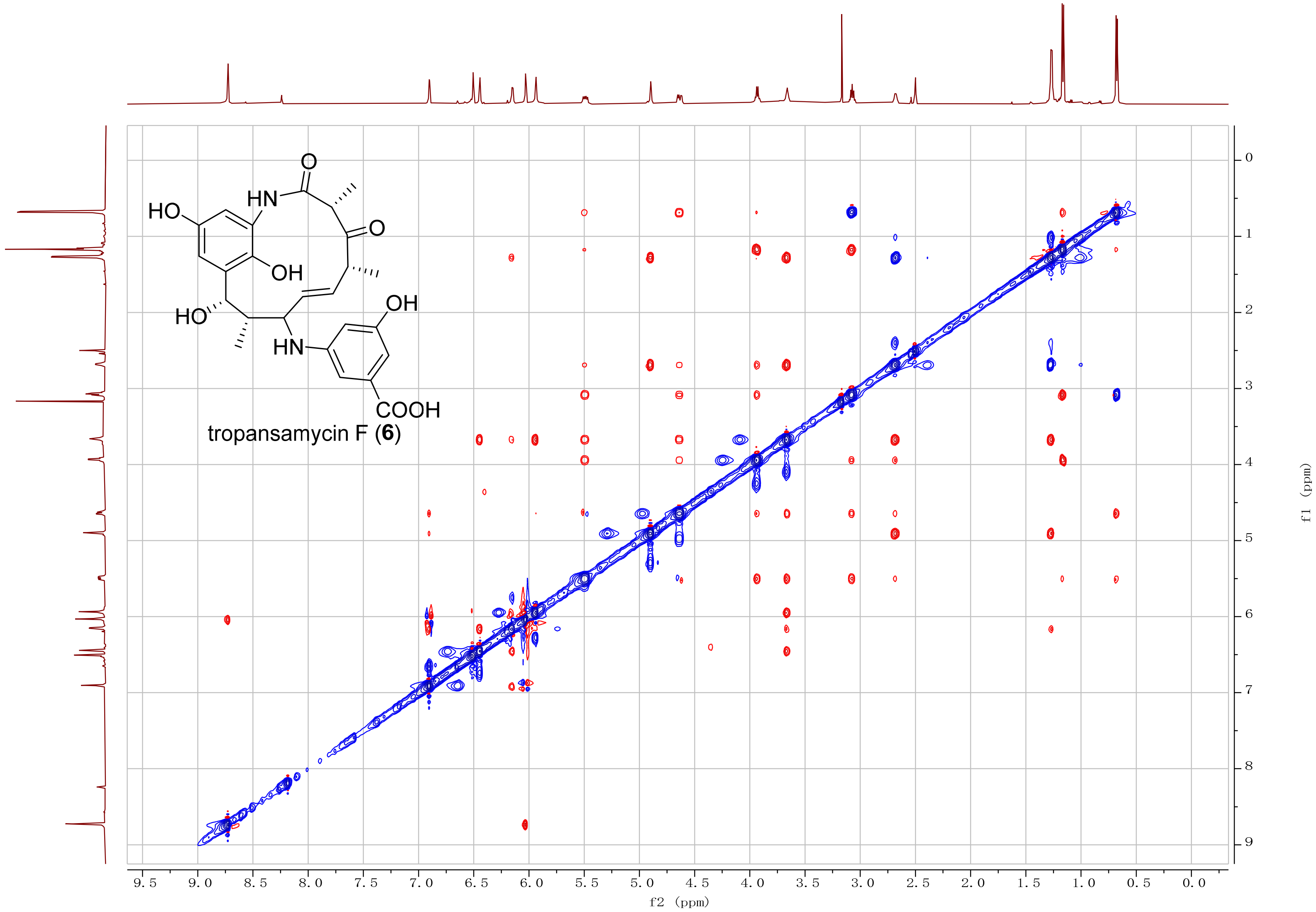

Figure S57. The ROESY spectrum of **6** in DMSO-d_6.

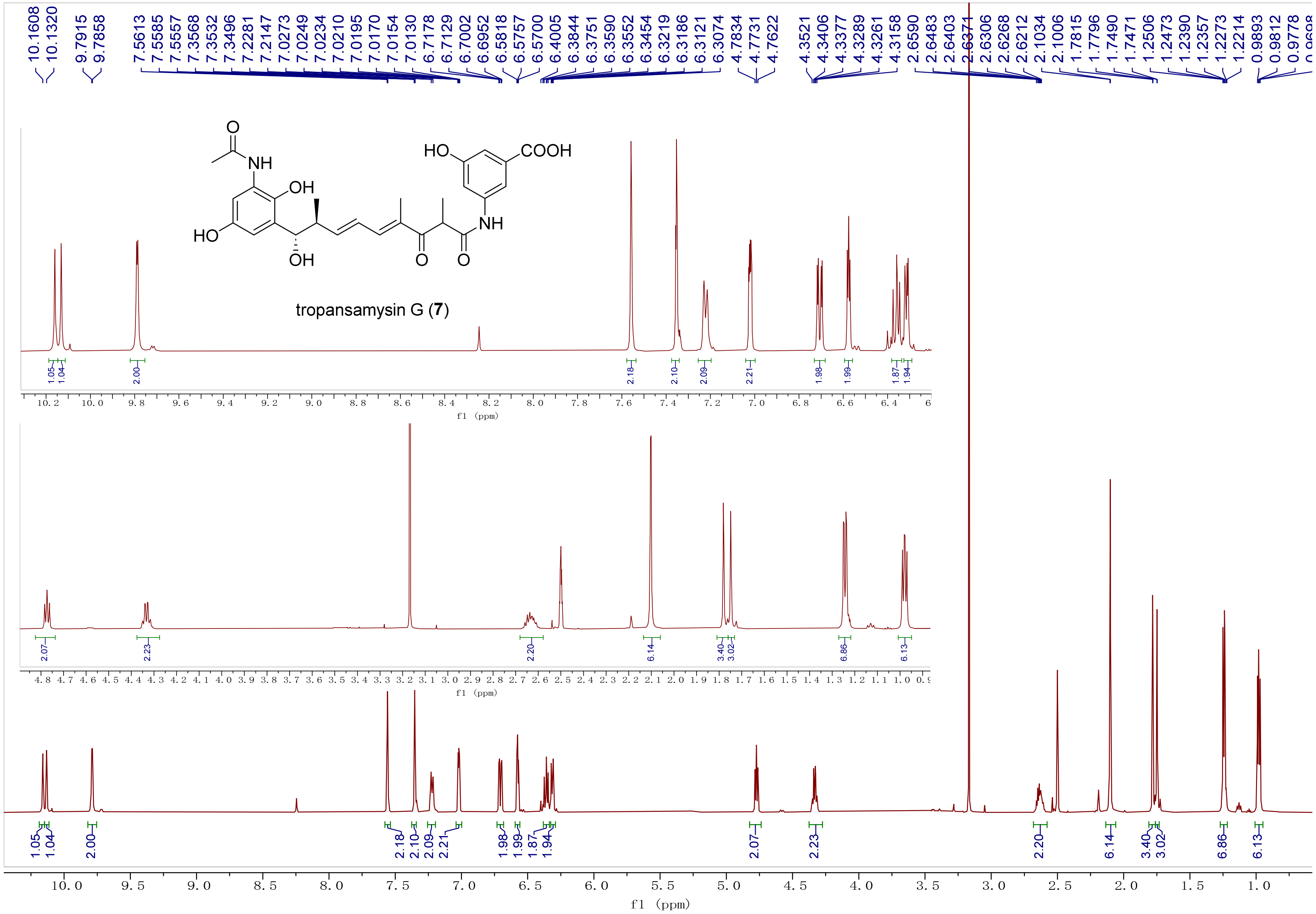

Figure S58. ^{1}H NMR spectrum of **7** in DMSO-d_6.

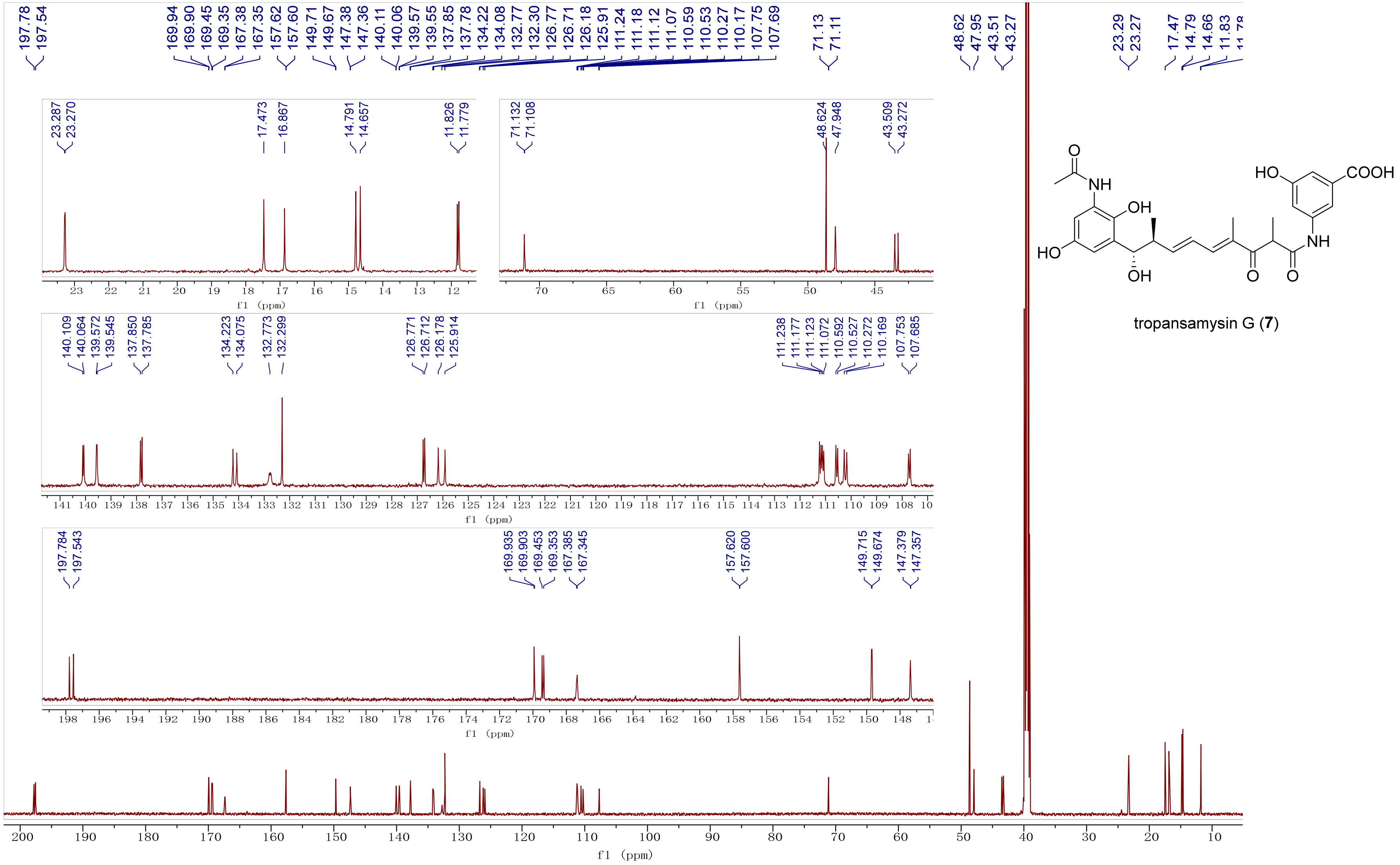

Figure S59. ^{13}C NMR spectrum of **7** in DMSO-d_6.

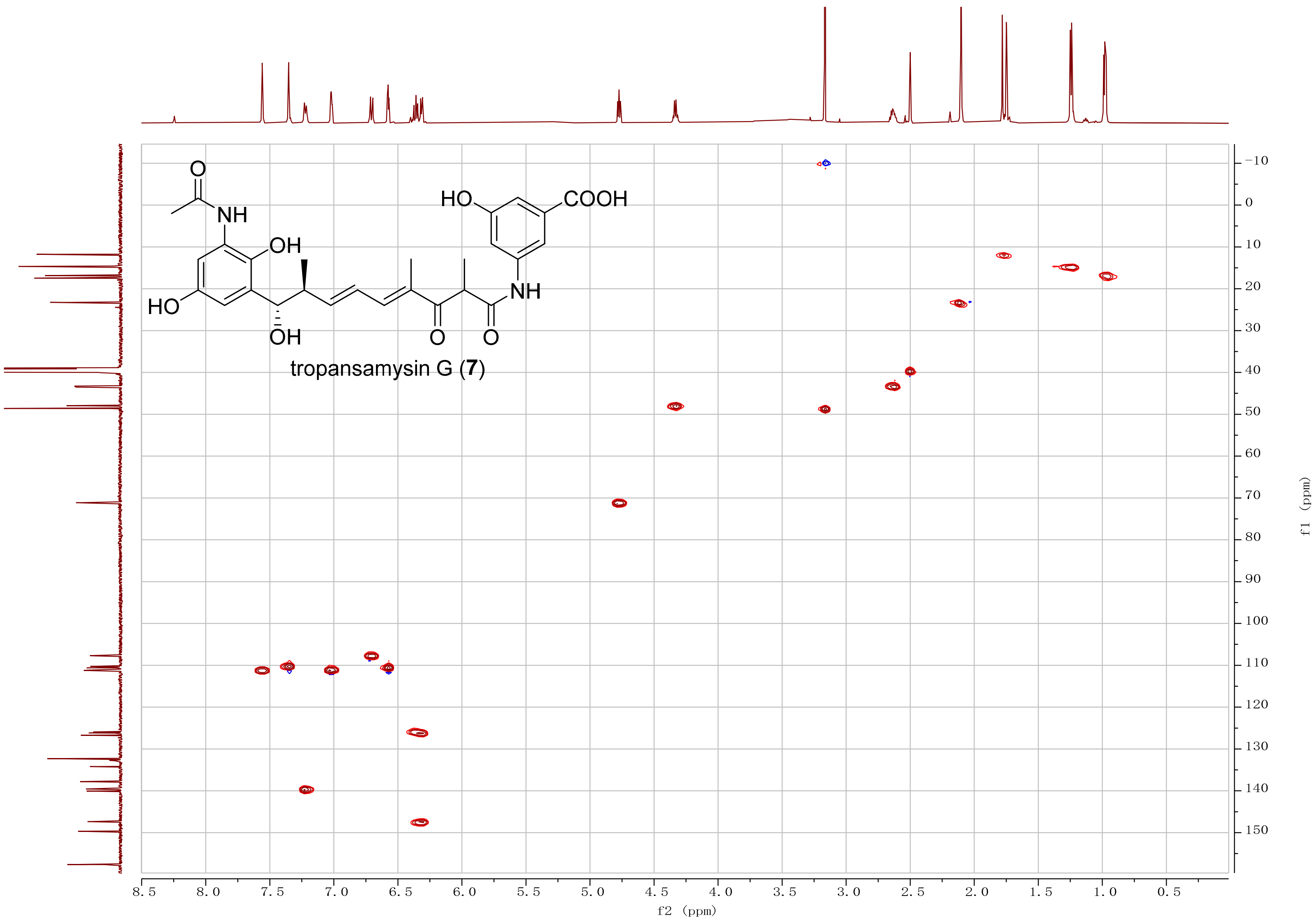

Figure S60. The HSQC spectrum of **7** in DMSO-d_6.

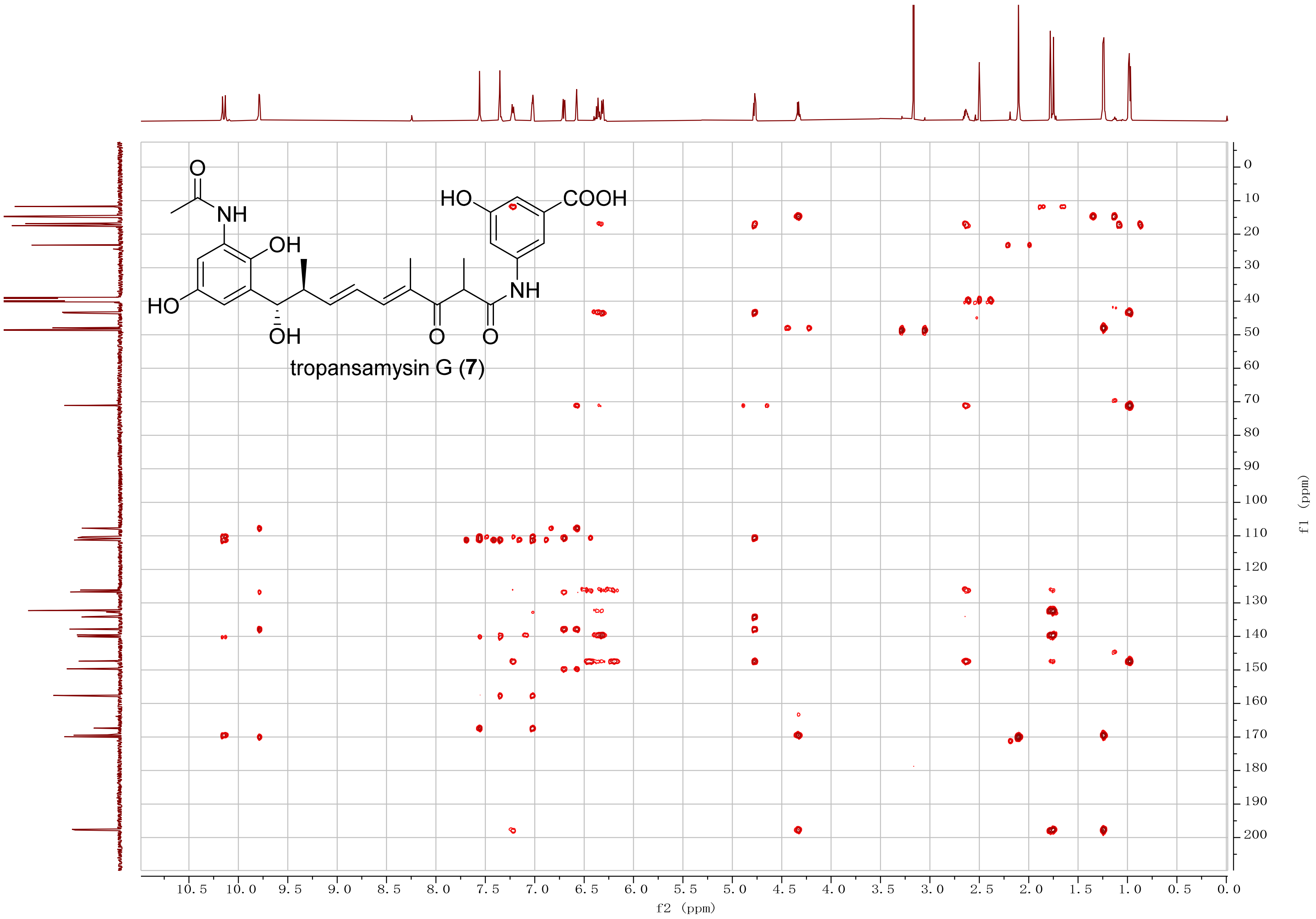

Figure S61. The HMBC spectrum of **7** in DMSO-d_6.

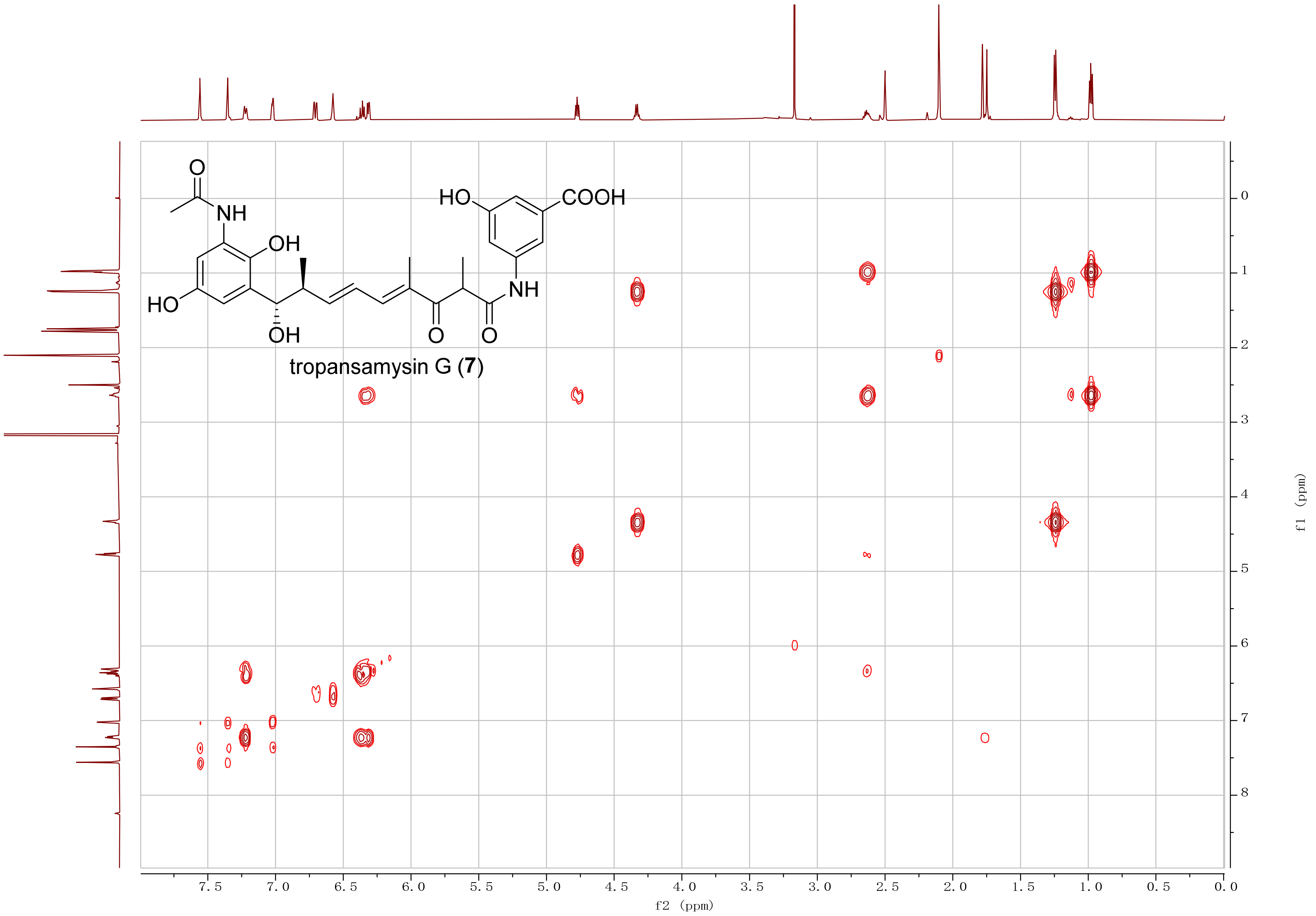

Figure S62. The ^{1}H-^{1}H COSY spectrum of **7** in DMSO-d_6.

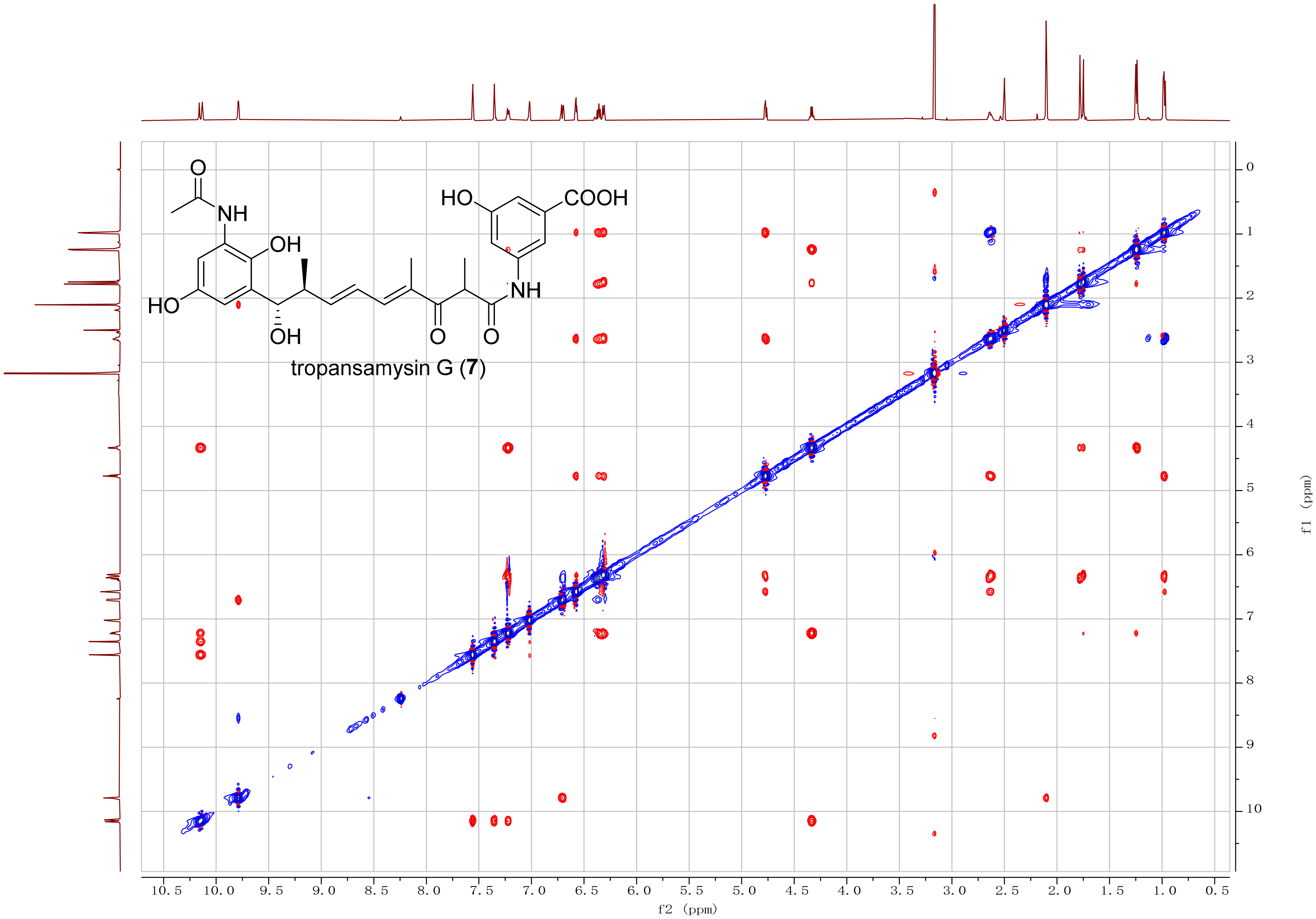

Figure S63. The ROESY spectrum of **7** in DMSO-d_6.

References

(1) Simon, R.; Priefer, U.; Pühler, A. A Broad Host Range Mobilization System for in vivo Genetic Engineering: Transposon Mutagenesis in Gram Negative Bacteria. *Bio/Technology* **1983**, *1* (9), 784–791.
(2) Jiao, Y.; Liu, Y.; Wang, H.; Zhu, D.; Shen, Y.; Li, Y. Expression of the Clifednamide Biosynthetic Pathway in *Streptomyces* Generates 27,28-*seco*-Derivatives. *J. Nat. Prod.* **2020**, *83* (9), 2803–2808.
(3) Bierman, M.; Logan, R.; O'Brien, K.; Seno, E. T.; Rao, R. N.; Schoner, B. E. Plasmid Cloning Vectors for the Conjugal Transfer of DNA from *Escherichia coli* to *Streptomyces* spp. *Gene* **1992**, *116* (1), 43–49.
